# Supplementary material for: Back to life: Techniques for developing high-quality 3D reconstructions of plants and animals from digitized specimens
Source: PLoS One. 2023 Mar 29;18(3):e0283027. doi: 10.1371/journal.pone.0283027 (PMC10058149; doi:10.1371/journal.pone.0283027)
Supplement: S1 File — (DOCX) [file pone.0283027.s001.docx]

**Supplemental Materials**

This is the supplementary material for *Back to life: Techniques for developing high-quality 3D reconstructions of plants and animals from digitized specimens*

**This includes:**

- Beginner tutorial: Fossil plant cupule (*Petriellaea triangulata* [Shi et al. 2021])
  - *Included files:* Cupule Cleanup Tutorial.docx
  - *3D object files*: Cupule before.stl, Cupule before.obj
  - *Necessary software*: Meshlab
  - *Skills developed*: Shrinkwrapping, simplification
- Intermediate tutorial: Reptile (*Sphenodon punctatus* [downloaded from Morphosource.org])
  - *Included files*: Sphenodon Cleanup Tutorial.docx
  - *3D object files*: Sphenodon with holes.stl, Sphenodon with holes.obj
  - *Necessary software*: Meshlab, Autodesk Maya, Autodesk Fusion 360
  - *Skills developed*: Rearticulation, repair of broken fossils, basic sculpting, shrinkwrapping.
- Advanced tutorial: Fossil crab (*Cretapsara athanata* [Luque et al. 2021)]
  - *Included files:* Crab Cleanup Tutorial.docx
  - *3D object files:* Crab before.stl, Crab before.obj
  - *Necessary software*: Meshlab, Autodesk Maya
  - *Skills developed*: Debris removal, structural repair, shrinkwrapping, rearticulation, smoothing and simplification

Tutorials and 3D Object Files are available at <https://doi.org/10.6084/m9.figshare.21266568>

Meshlab is an opensource software available at <https://www.meshlab.net/>

Autodesk Maya and Fusion 360 are available with a student and educators license at <https://www.autodesk.com/education/edu-software/overview>

**3D Mesh Cleanup Tutorial: Fossil Plant Cupule (Beginner)**

*Skills developed*: Basic mesh editing

3D meshes of fossil specimens extracted directly from 3D imaging data often need post-processing to improve usability for paleontological analyses. Here, we present a step-by-step workflow that was used to digitally process a cupule of the plant fossil *Petriellaea triangulata* (Shi et al. 2021). This tutorial uses the opensource mesh editing program Meshlab that can be downloaded at meshlab.net.


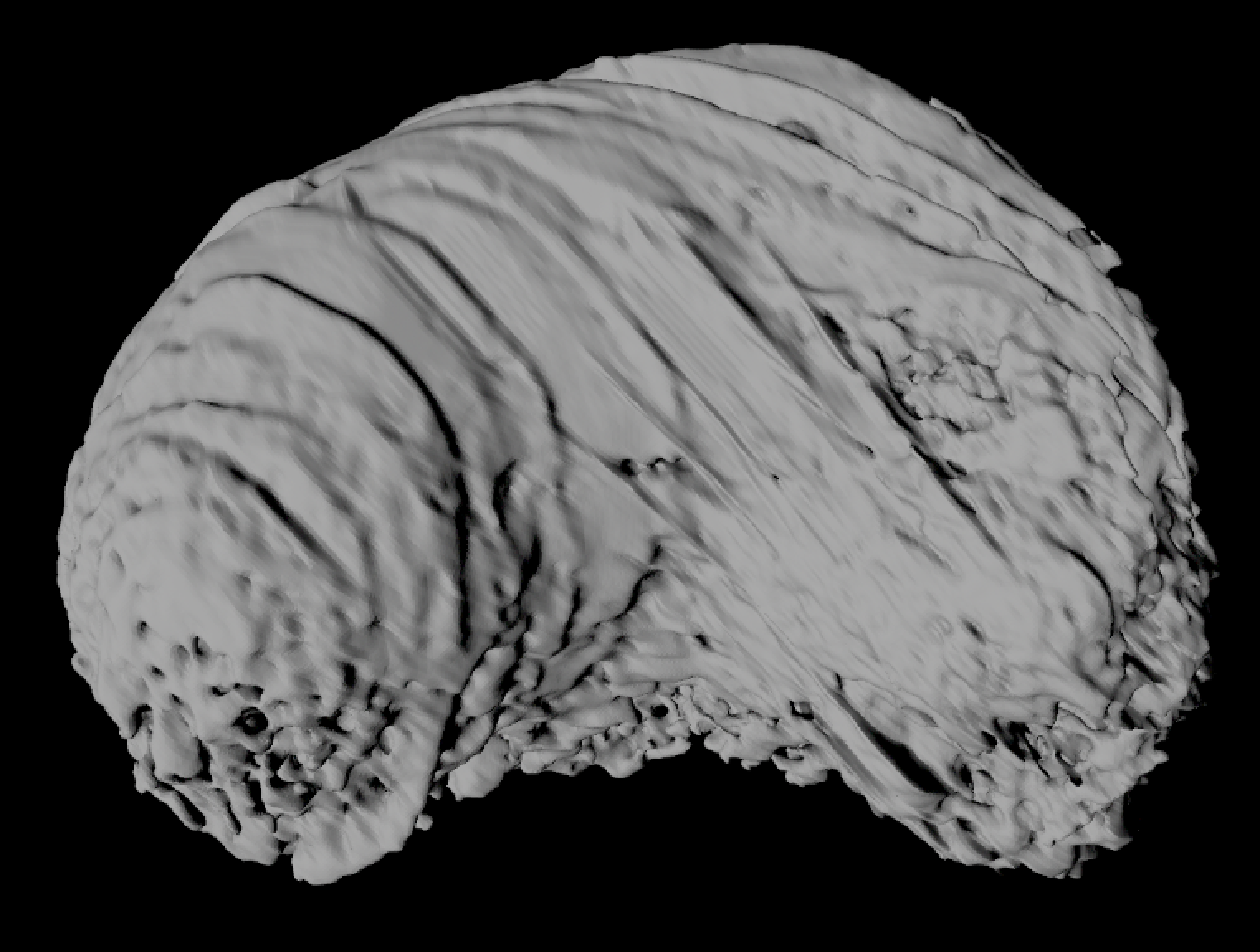


When the digitized fossil cupule was first exported as a 3D object file from the 3D processing software (Avizo Lite v. 9.2.0), a number of common issues were evident. The ridges, rough surfaces, and holes present are preservational artifacts and/or a consequence of the digital extraction process. This tutorial will walk through how to fix these issues in Meshlab (Cignoni et al. 2008). Note that this tutorial utilizes Meshlab 2021.10, though other versions will have similar layouts and functions.

Part 1: The Meshlab Interface


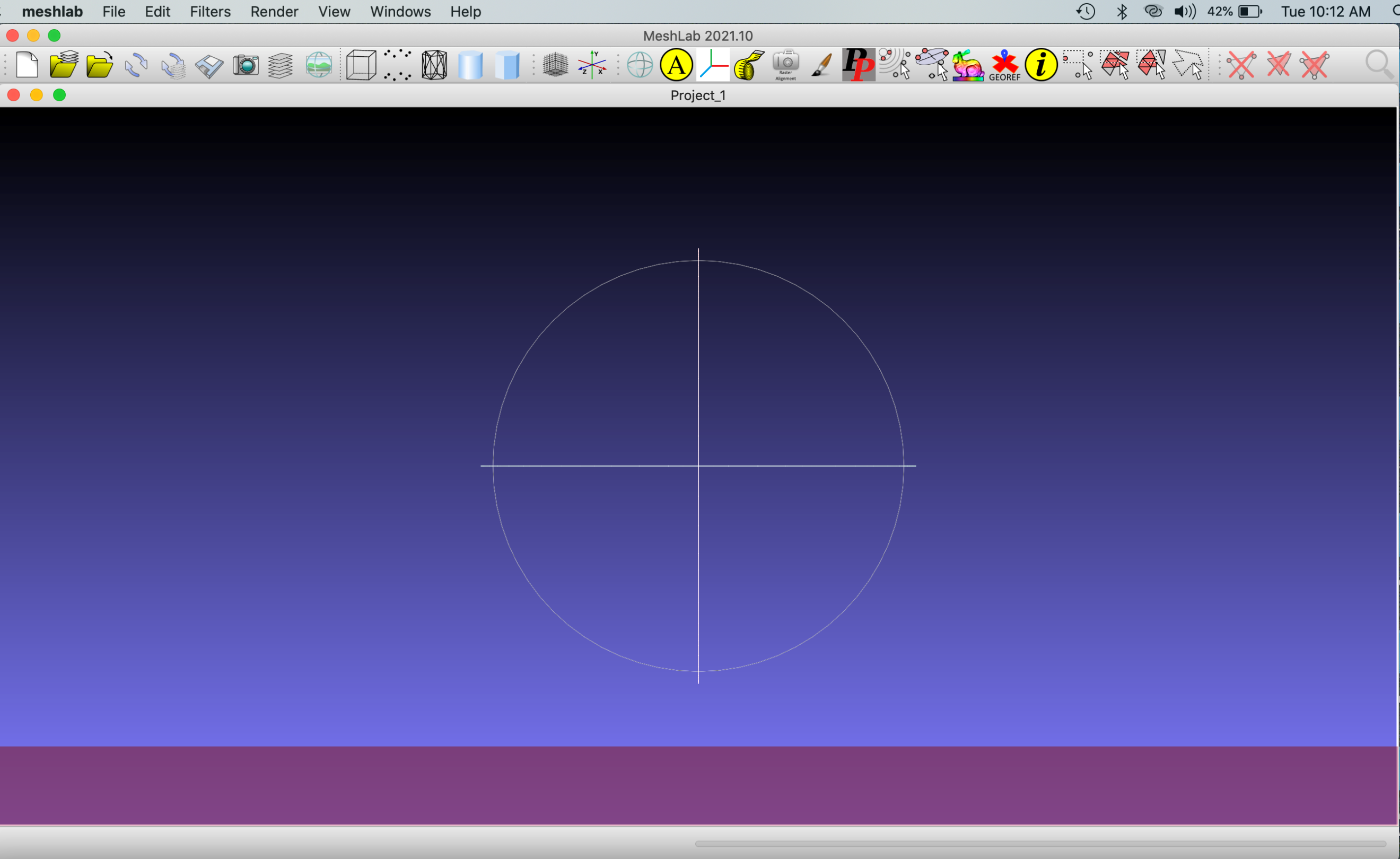


Open Meshlab and import “Cupule before.obj” into Meshlab via **File** > **Import Mesh**. Alternatively, drag the file into the project window (the large purple box with the crosshairs) if using Mac. **Keep the “unify duplicate vertices” box checked**. The 3D object file is made of a series of polygons. The polygons themselves are called the “faces,” and the points making up the corners are points called “vertices.” This is known as a mesh: a 3D object comprised of a series of faces and vertices. The 3D object file we imported is our fossil cupule represented digitally as a 3D mesh.


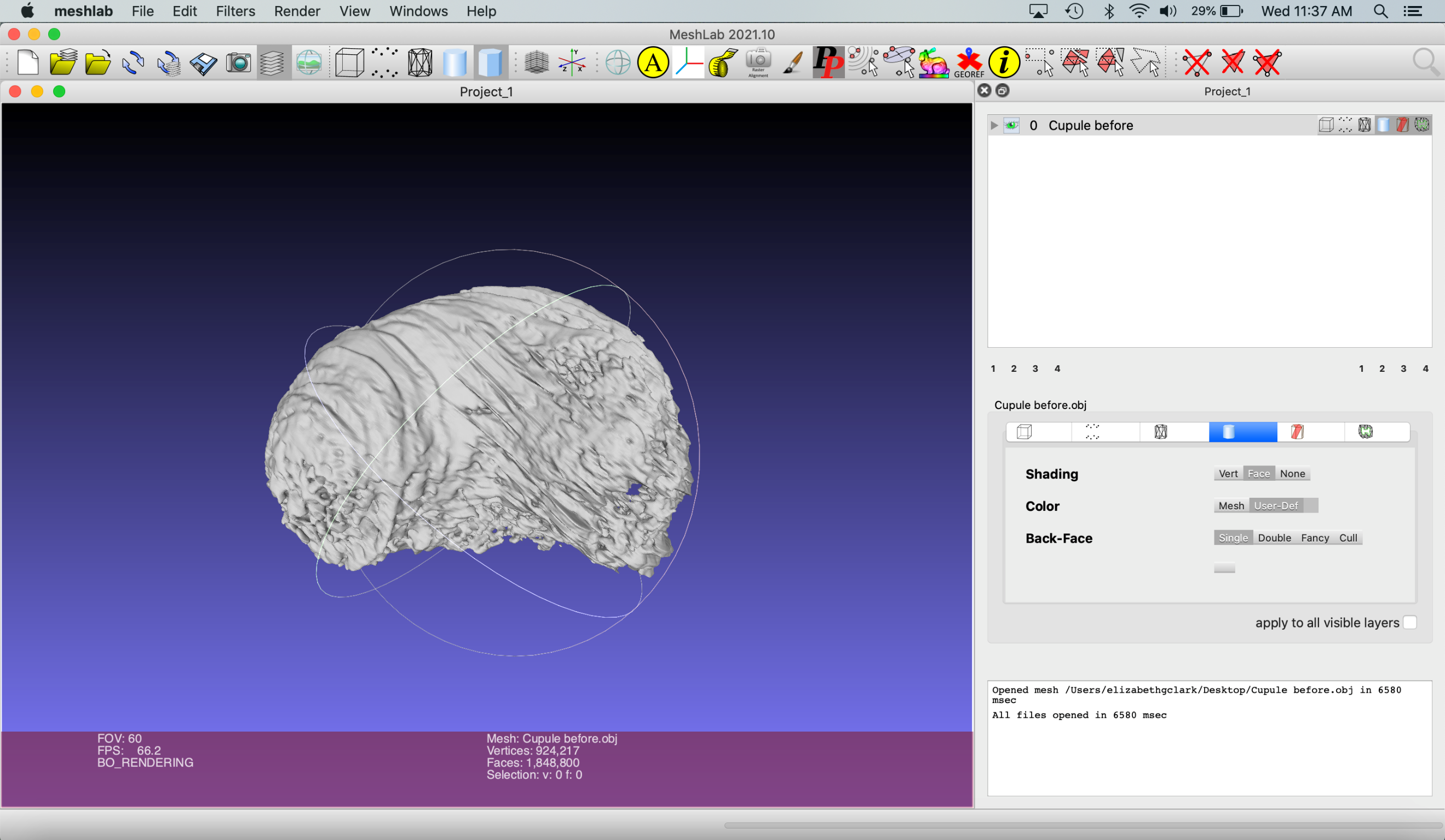


The mesh now appears in the project window. **You can rotate it by holding left click and dragging the cursor.** When the file imported, three new menus appeared on the right. The top menu lists the objects that are in the scene. The center menu lists attributes of the object. The bottom menu keeps a history of commands performed.

**Try repositioning the mesh.** On a Mac, this is done by holding the command key, holding the left mouse button, and dragging the mesh. On PC, hold ctrl and the left mouse button.


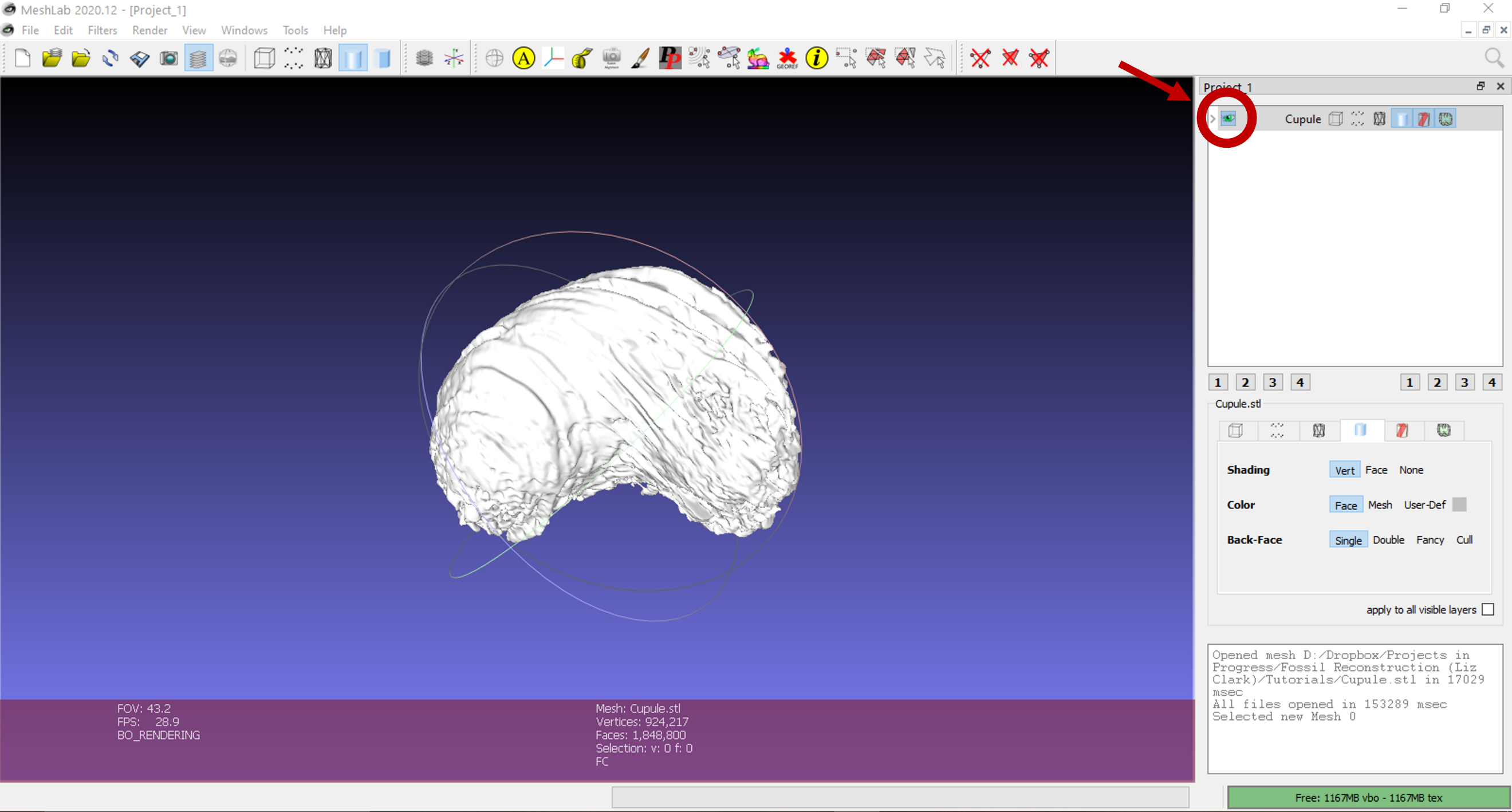


**Turn the visibility of the object on and off by clicking on the eye icon in the top right menu**.

**Zoom in by scrolling with the wheel of a mouse, swiping with two fingers (on a Mac trackpad), or holding down shift and left click (in both Mac and PC) while moving the cursor up and down.**

Part 2: Shrinkwrapping the Mesh

Create a new, simplified mesh over the surface of the cupule. This is known as “shrinkwrapping.” Shrinkwrapping the mesh smooths the surface and patches holes. It is performed by isolating the vertices that make up the external structure of the cupule and using them to make a new mesh layer. The first step is to hollow out – or remove the faces and vertices - from the inside of the mesh. The program will label which faces and vertices are inside the cupule and which are on the surface by clicking **Filters** > **Color Creation and Processing** > **Ambient Occlusion**. This filter colors the internal mesh parts dark and the internal mesh parts white.


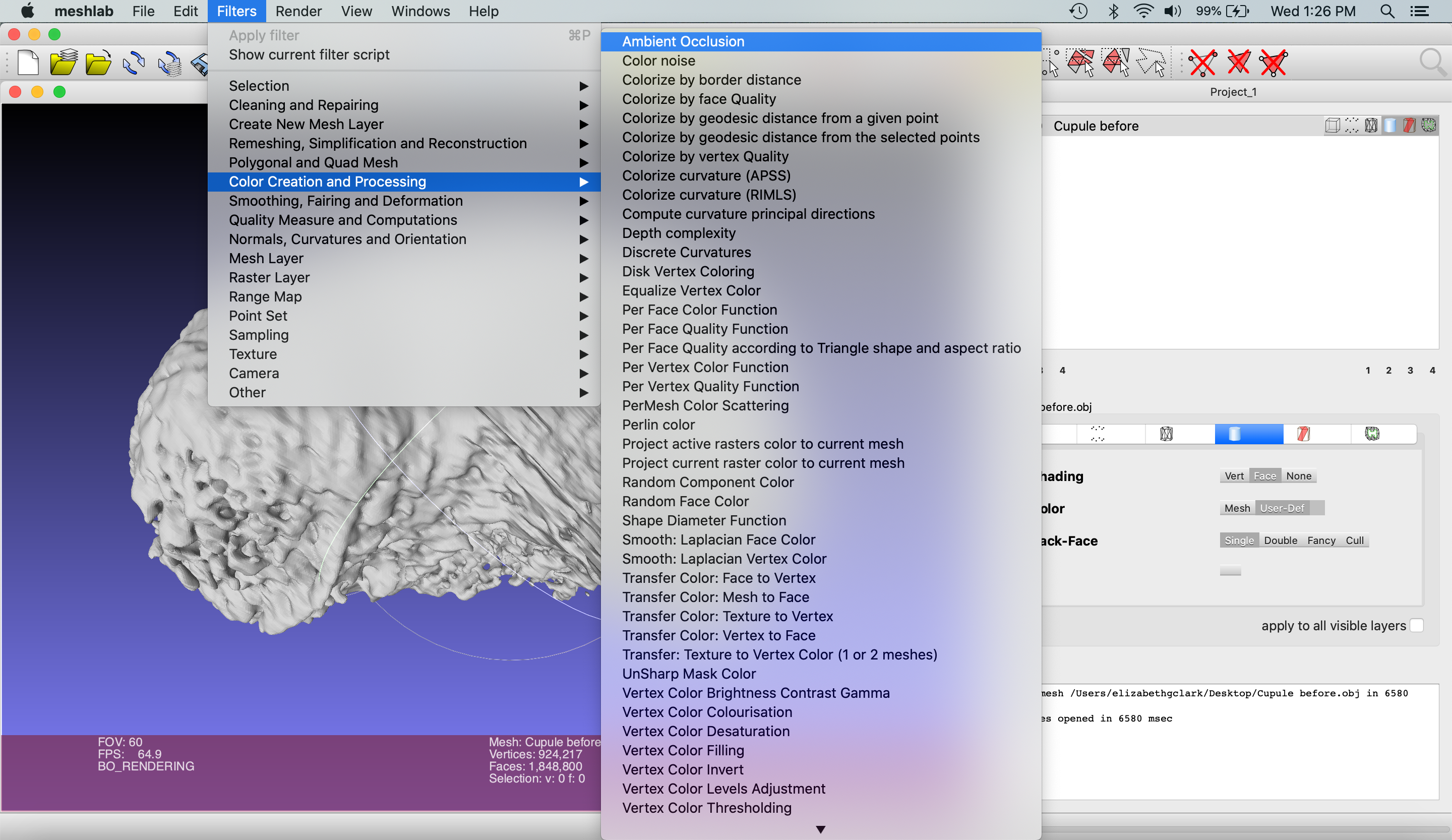


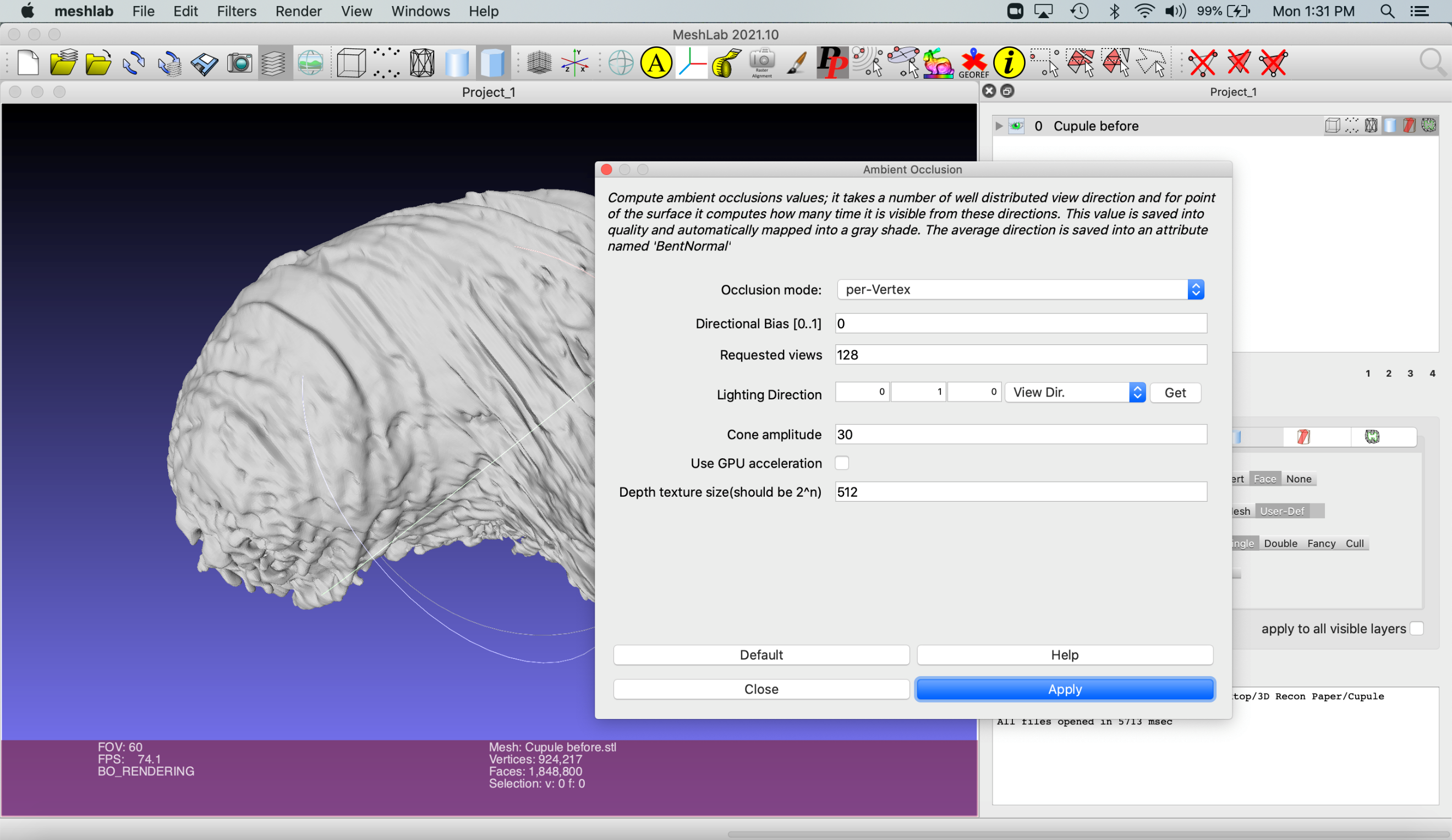


**Click** **Apply** on the pop-up menu. All other settings can remain as default.


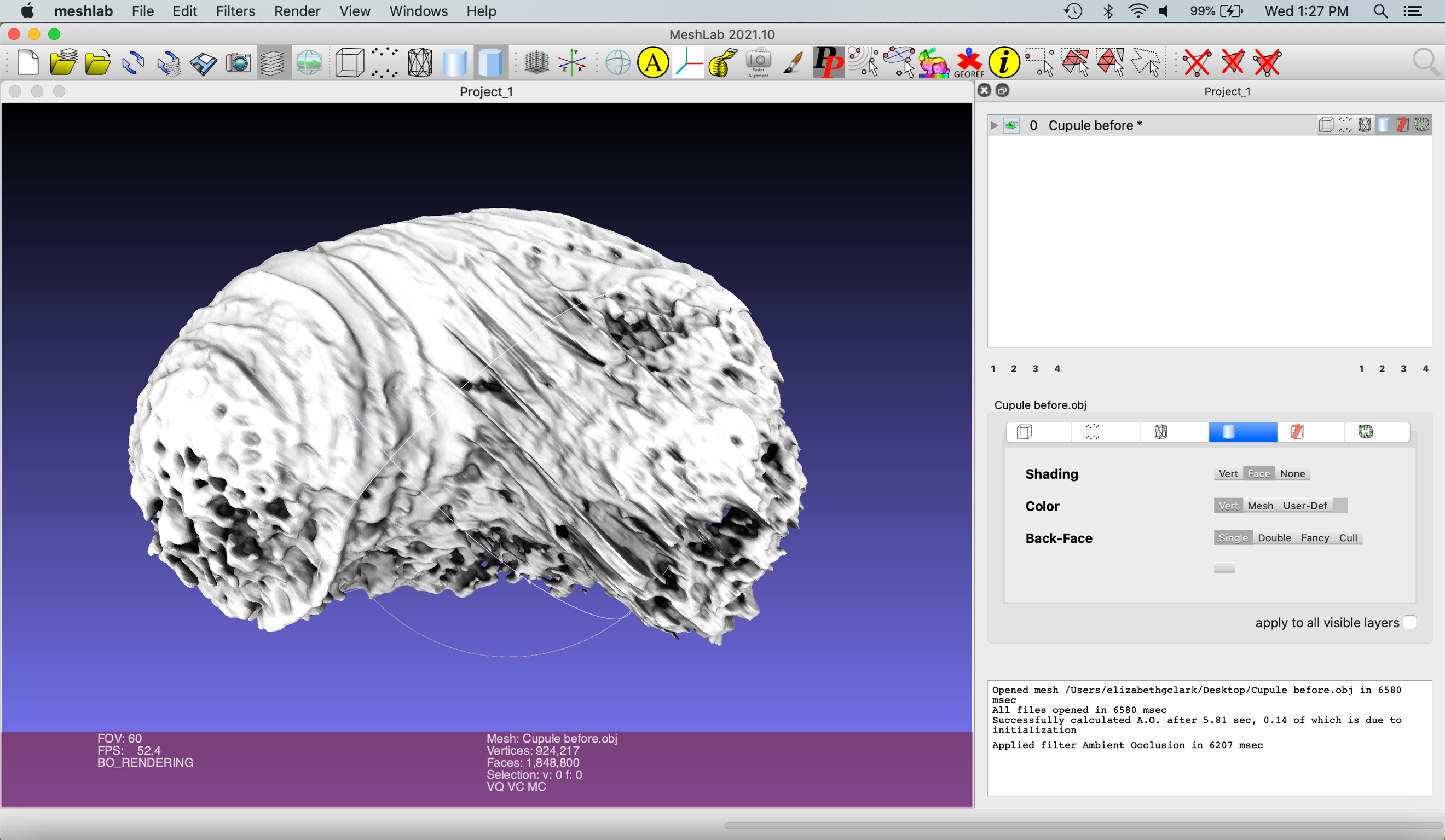


Next, select the dark mesh components by clicking **Filters** > **Selection** > **Select by Vertex Quality**.


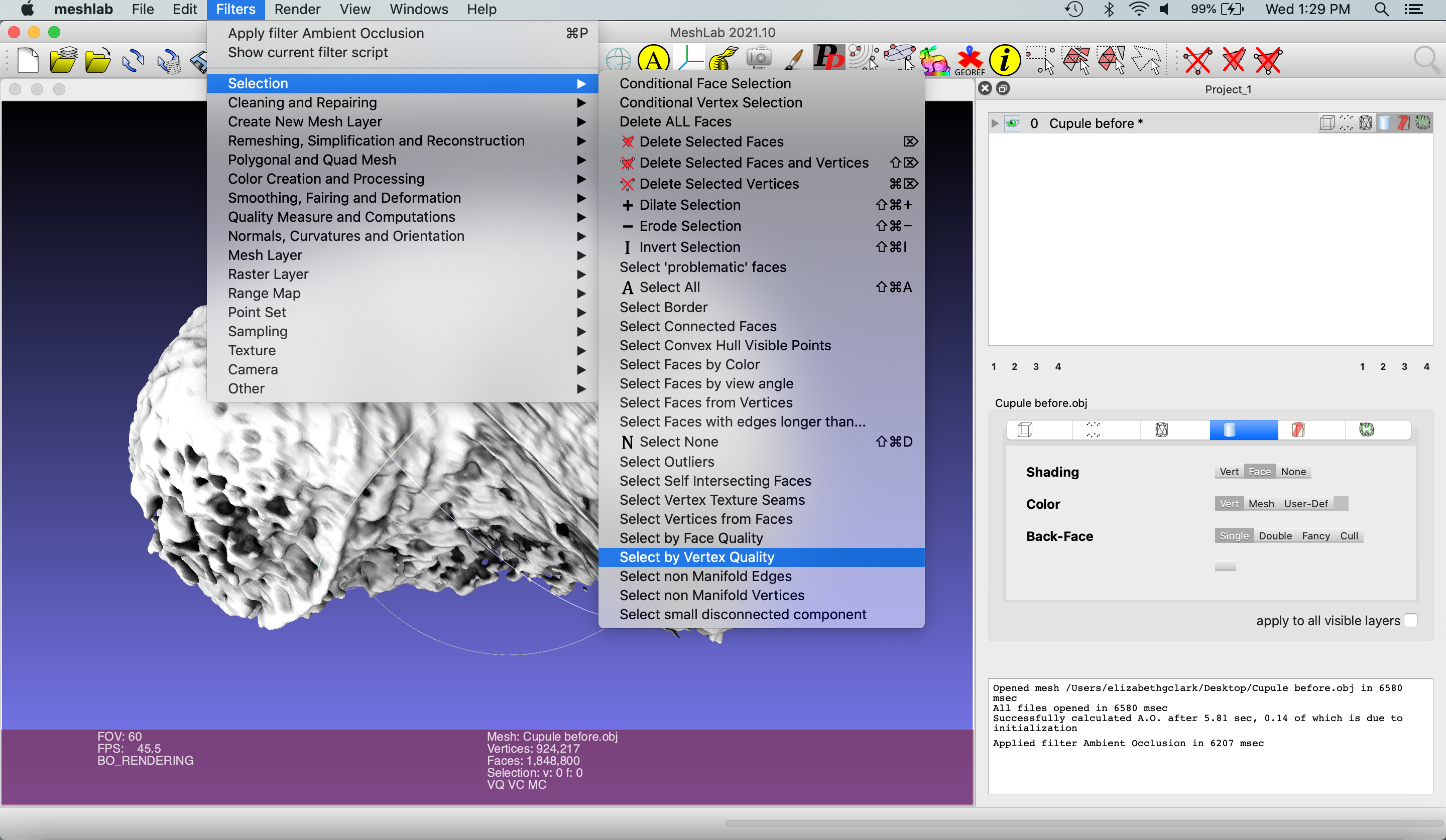


This selects mesh components with greyscale values within a set window. Select the darkest values, since these are the components located on the inside of the mesh. To do this, **set the “Min Quality” value to 0**. Next, **check the “Preview” box**, and a preview of the selected mesh components within that greyscale window will be highlighted in red.


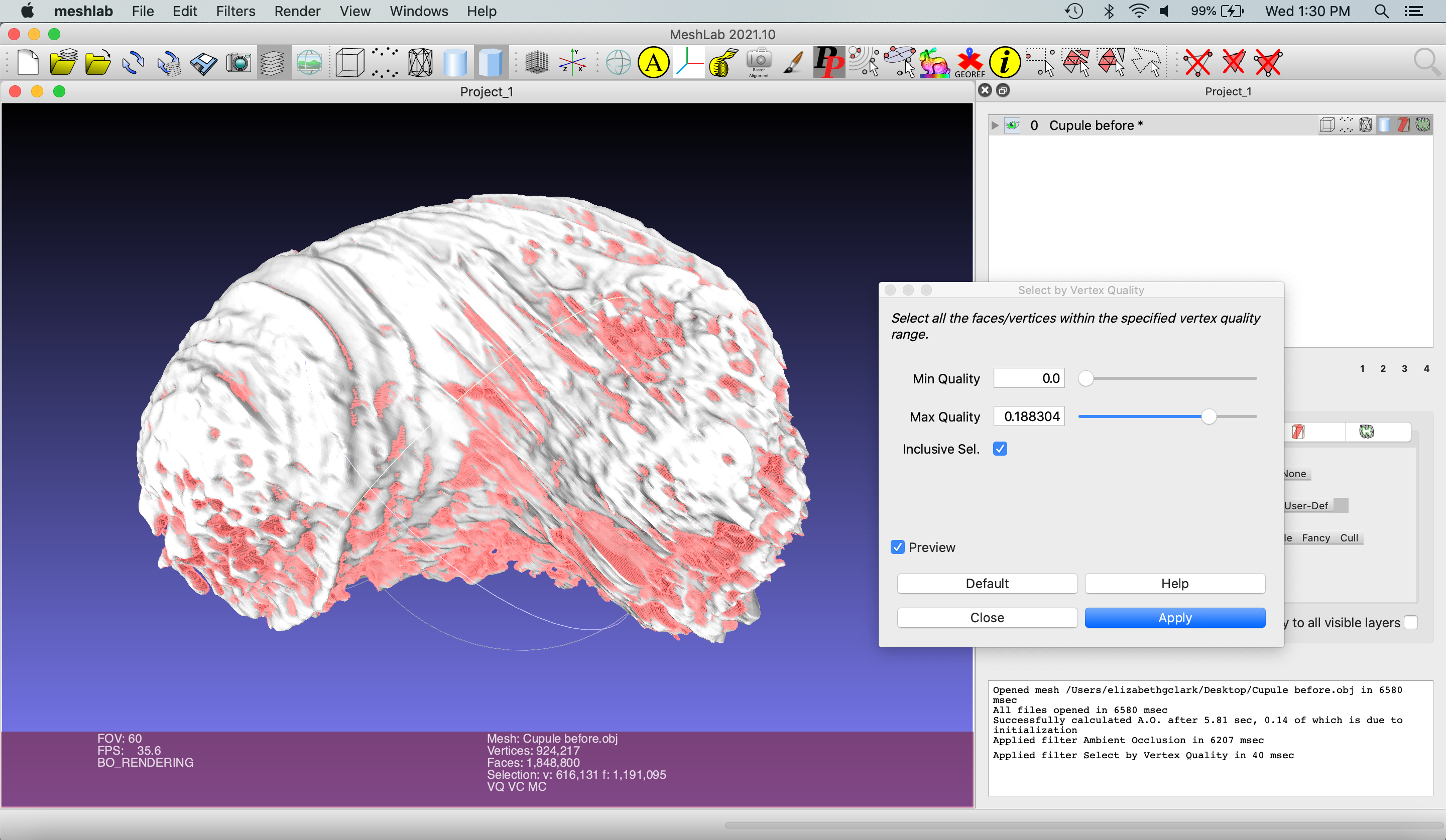


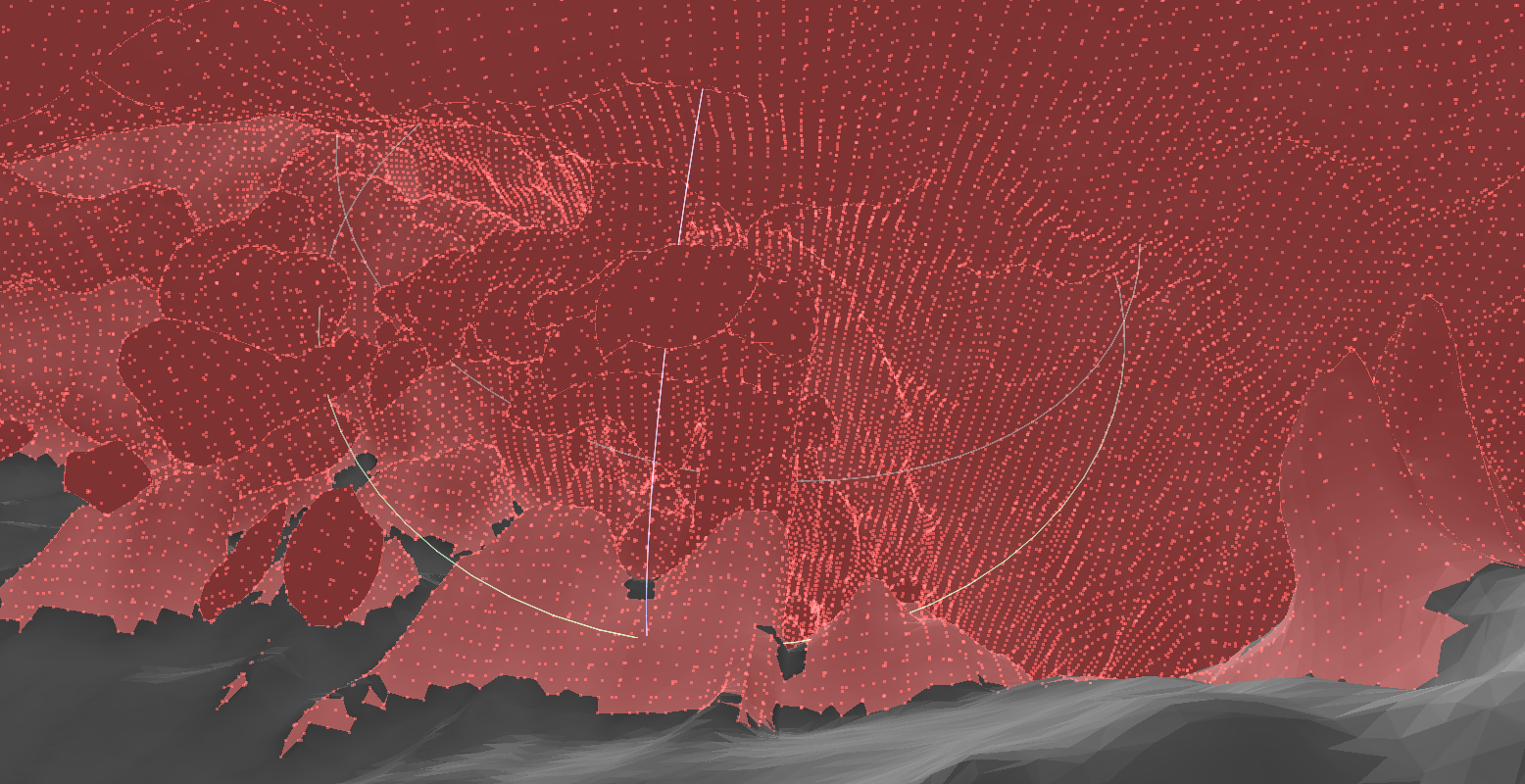

View of the inside of the cupule

Zoom in and explore the view of the previewed selection inside the object. Toggle the **“Max Quality”** slider until most of the inside components are selected. Then, zoom out to view the external surface of the object. The goal is to set Max Quality to select the lowest number of faces on the outside while selecting the highest number on the inside: Max Quality was set to 0.188304 in this example. Once you have achieved this balance, **hit “Apply.”**

Select **“Delete Selected Faces and Vertices”** on the right of the icon-based menu towards the top of the screen. This will delete the faces and vertices in red selected in the previous step, leaving a shell of the original mesh.


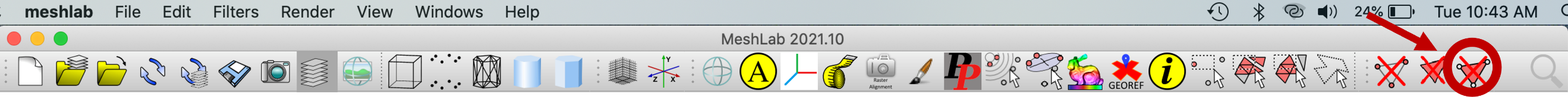


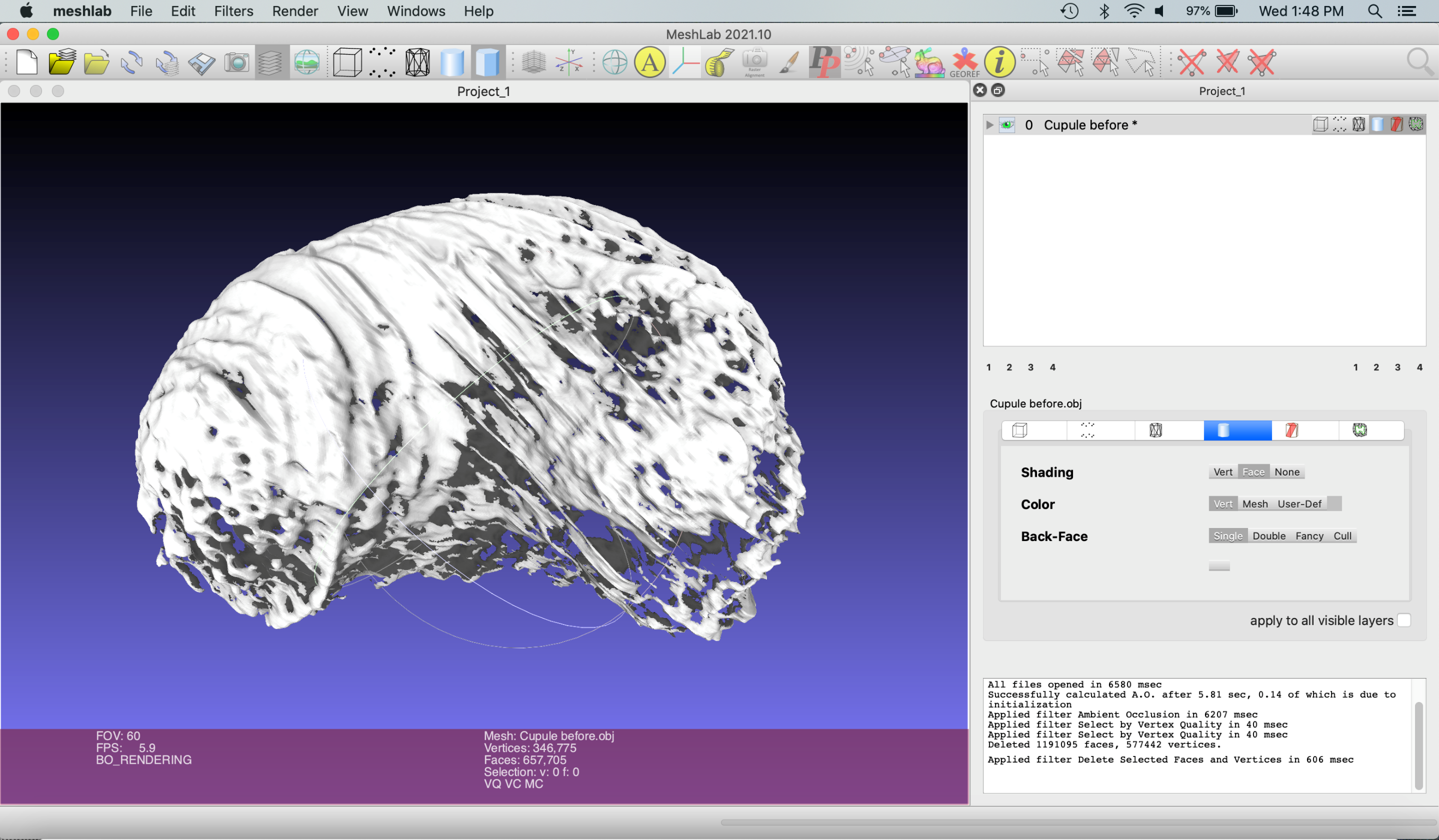


Meshlab does not have an undo function; periodically creating a duplicate of the current mesh is a good way to save a checkpoint. To do this, **Right click the mesh in the top menu > Duplicate Current Layer**.


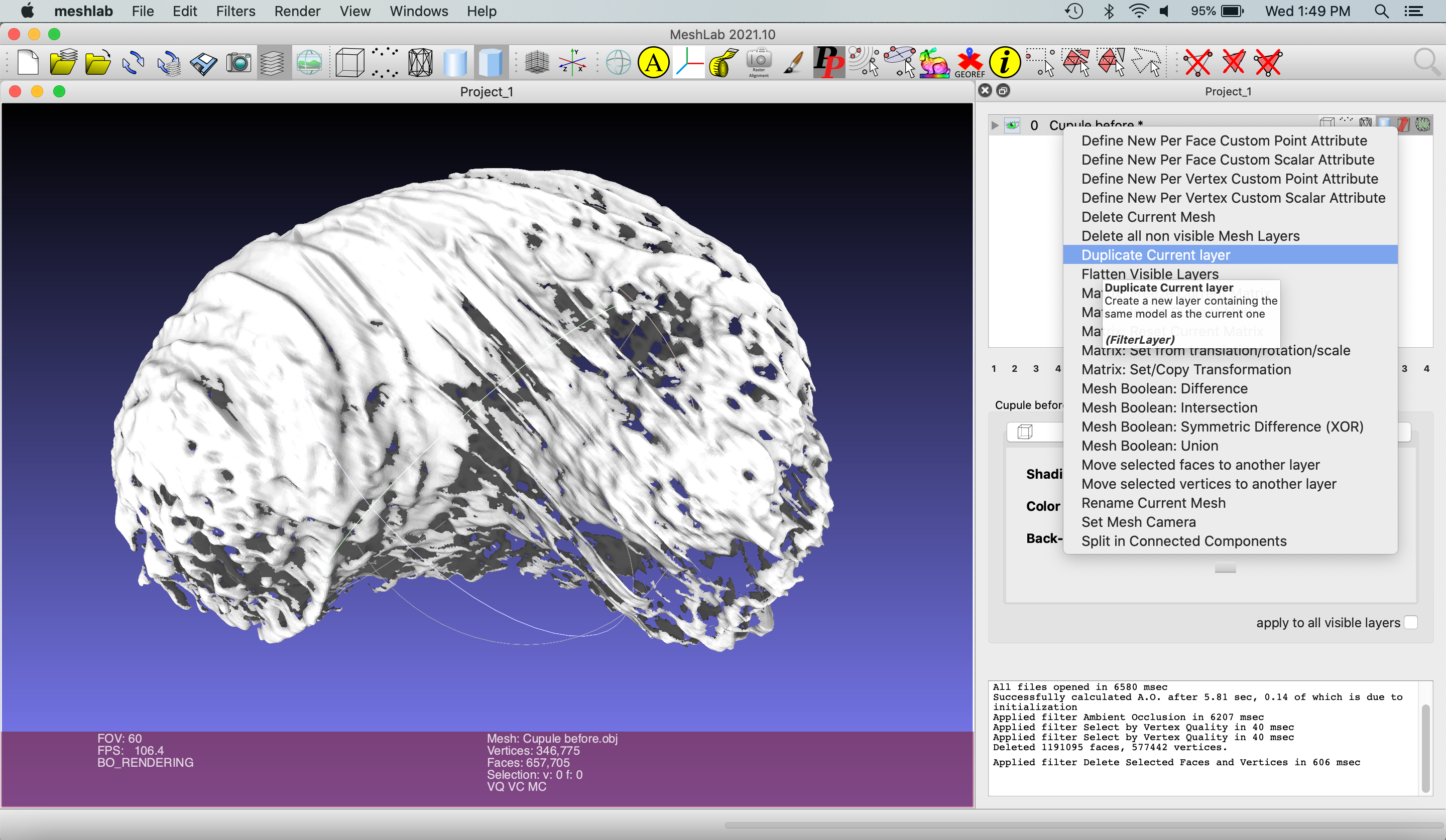


**Click the eye** in the top row to hide the original mesh. The eye will appear closed, indicating the layer is not visible. **Click the second row** to select the new mesh, since any commands performed will be done on the active selection.


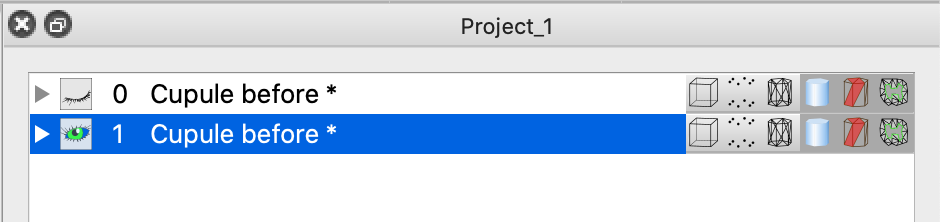


To remove all of the faces of the mesh**,** **select** **Filters** > **Selection** > **Select All** or **press command + shift + A** (Mac) or **ctrl + shift + A** (PC).


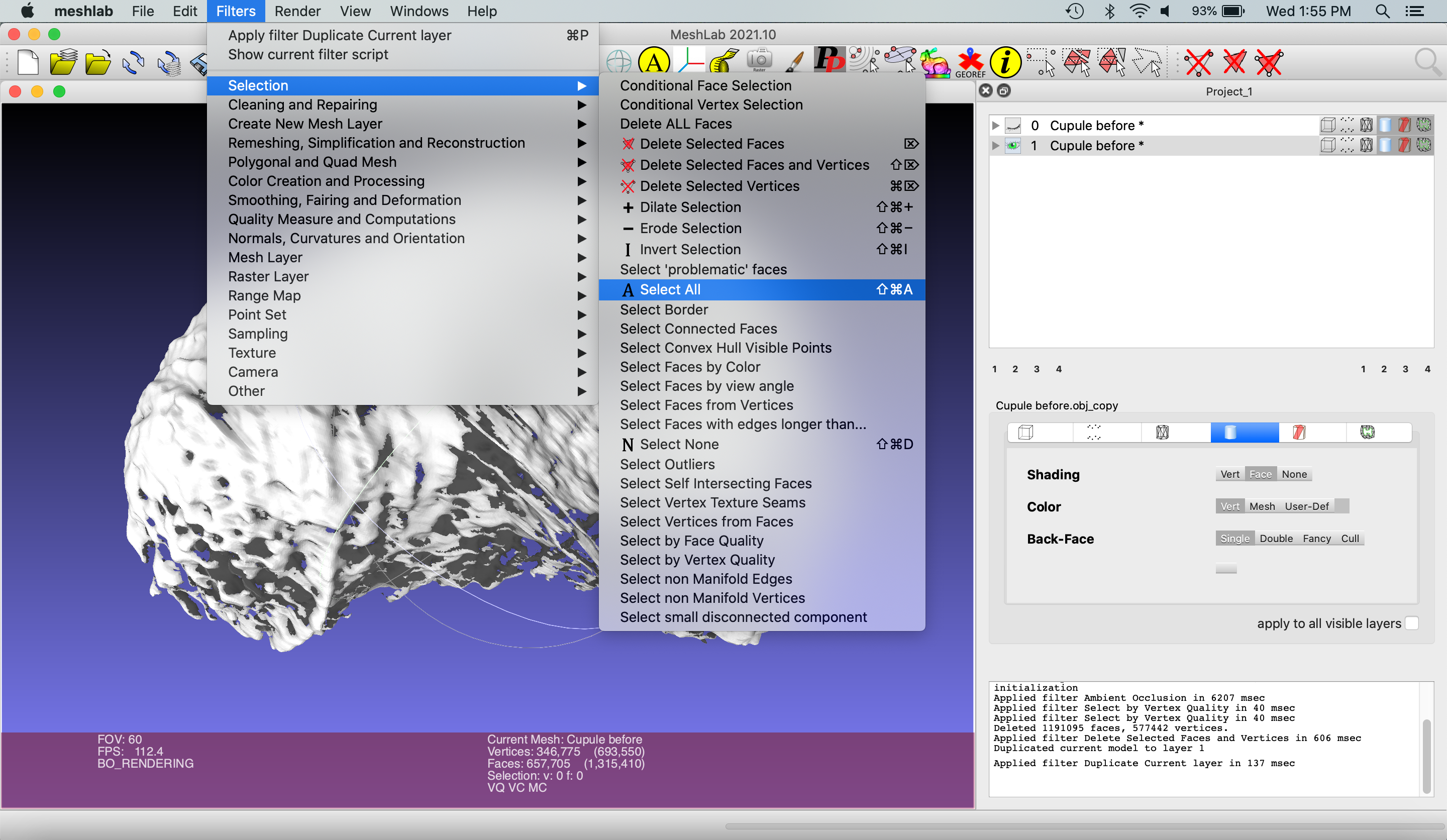


A pop-up menu will appear. Keep both boxed selected. Select **“Apply”**


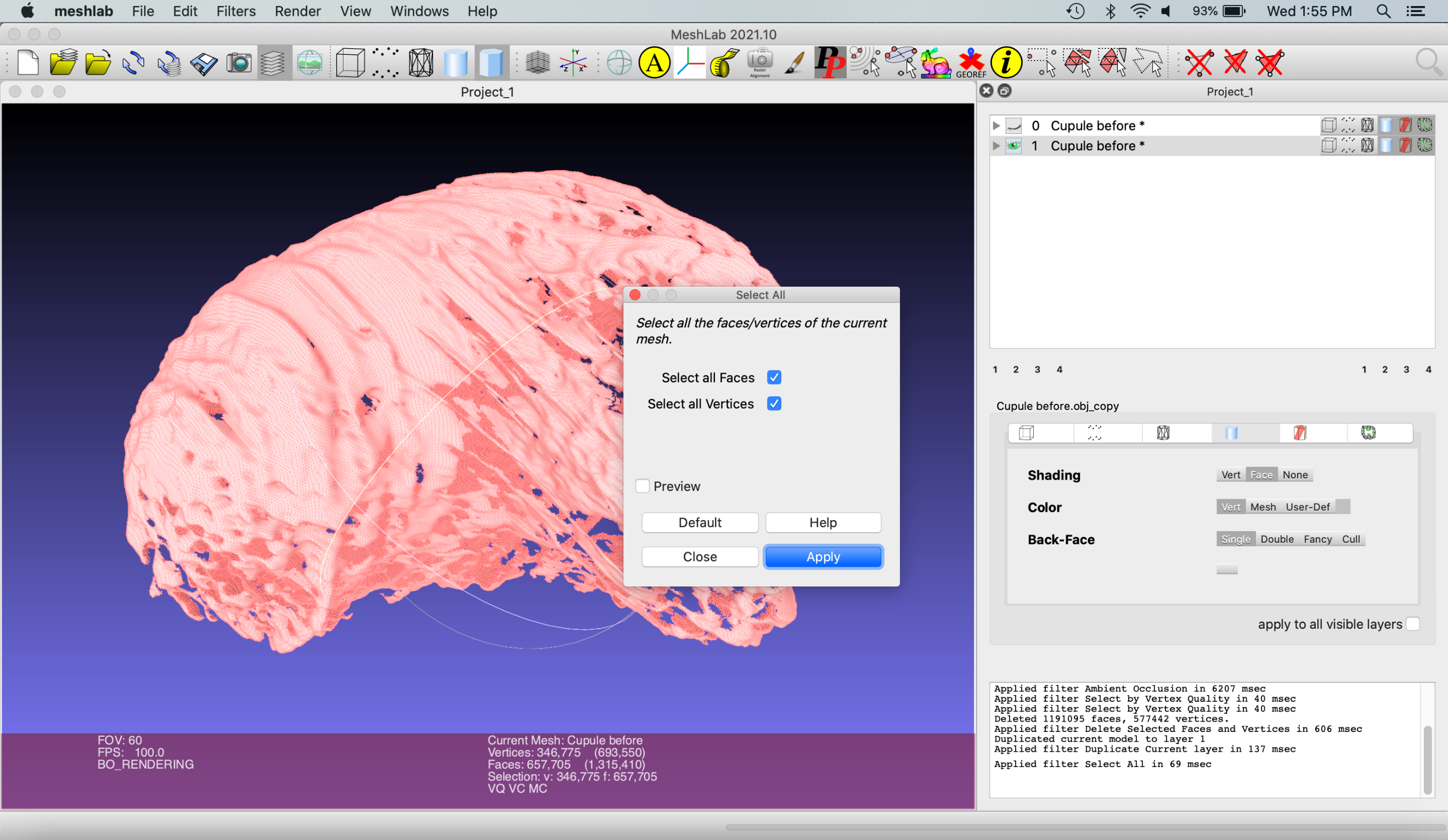


Delete all mesh faces by **selecting “delete faces” icon in the icon-based menu**:


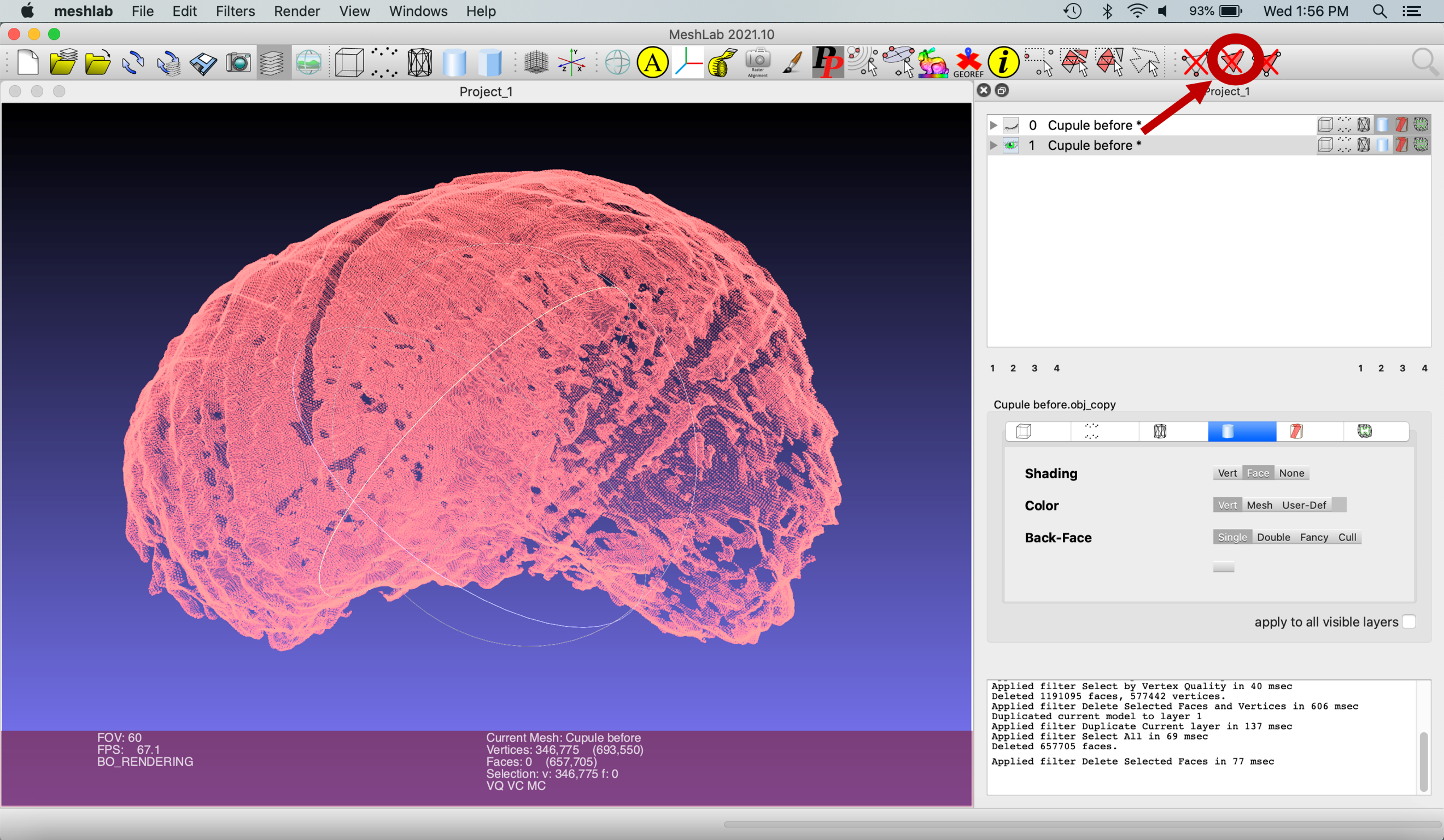


Next, the outer vertices of the cupule will be used to create a new mesh using a screened Poisson (Kazhdan and Hoppe, 2013). Go to **Filters** > **Remeshing, Simplification and Reconstruction** > **Surface Reconstruction: Screened Poisson**.


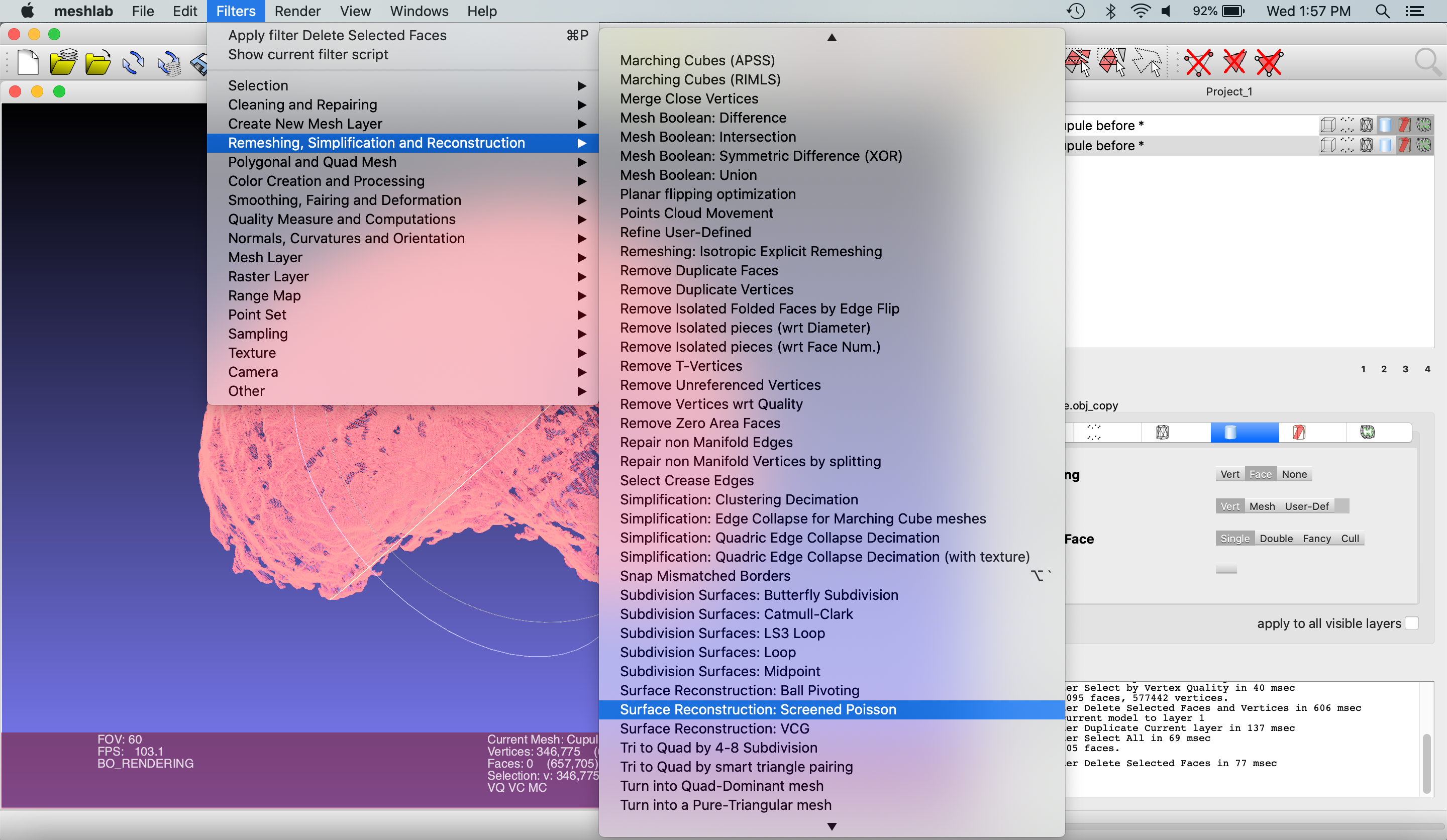


**Check the “pre-clean” box**, which removes any defects in the mesh that would keep the filter from working. **Click “Apply”** in the popup menu. A new layer named “Poisson mesh” will appear in the righthand menu. **Click the eye of your previous mesh to view the poisson mesh by itself**.


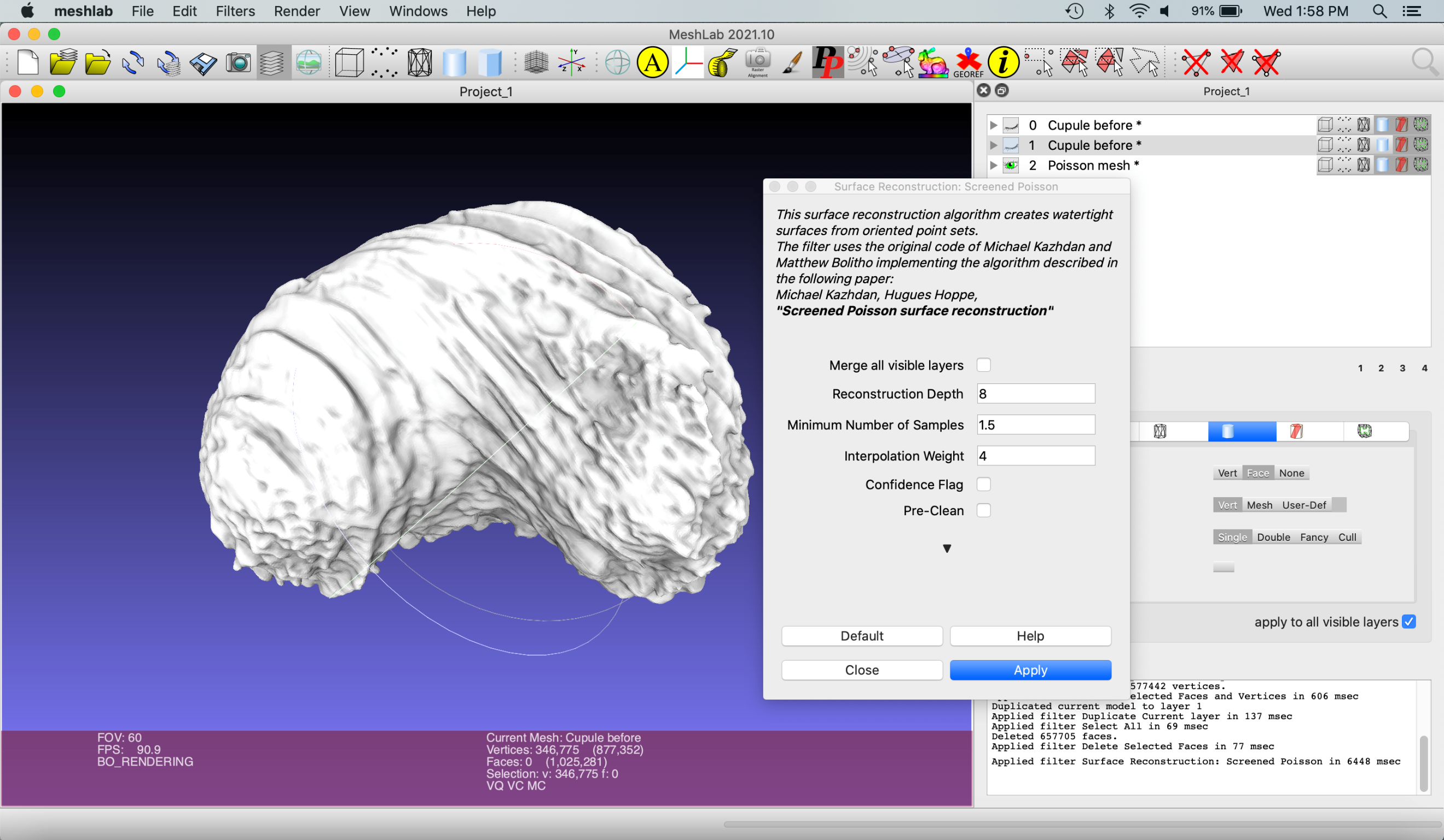


The “Reconstruction Depth” ranges from 1-12, with default value as 8. **To see what different reconstruction depths look like, re-select the second layer in the object list, “Cupule before,” and run the filter again with a reconstruction depth value of 4 and 12**. Toggle between viewing the new Poisson meshes on the right top menu by opening and closing the eye icon for each mesh. You will see that a higher reconstruction depth value preserves more detail, while a lower value applies more smoothing.


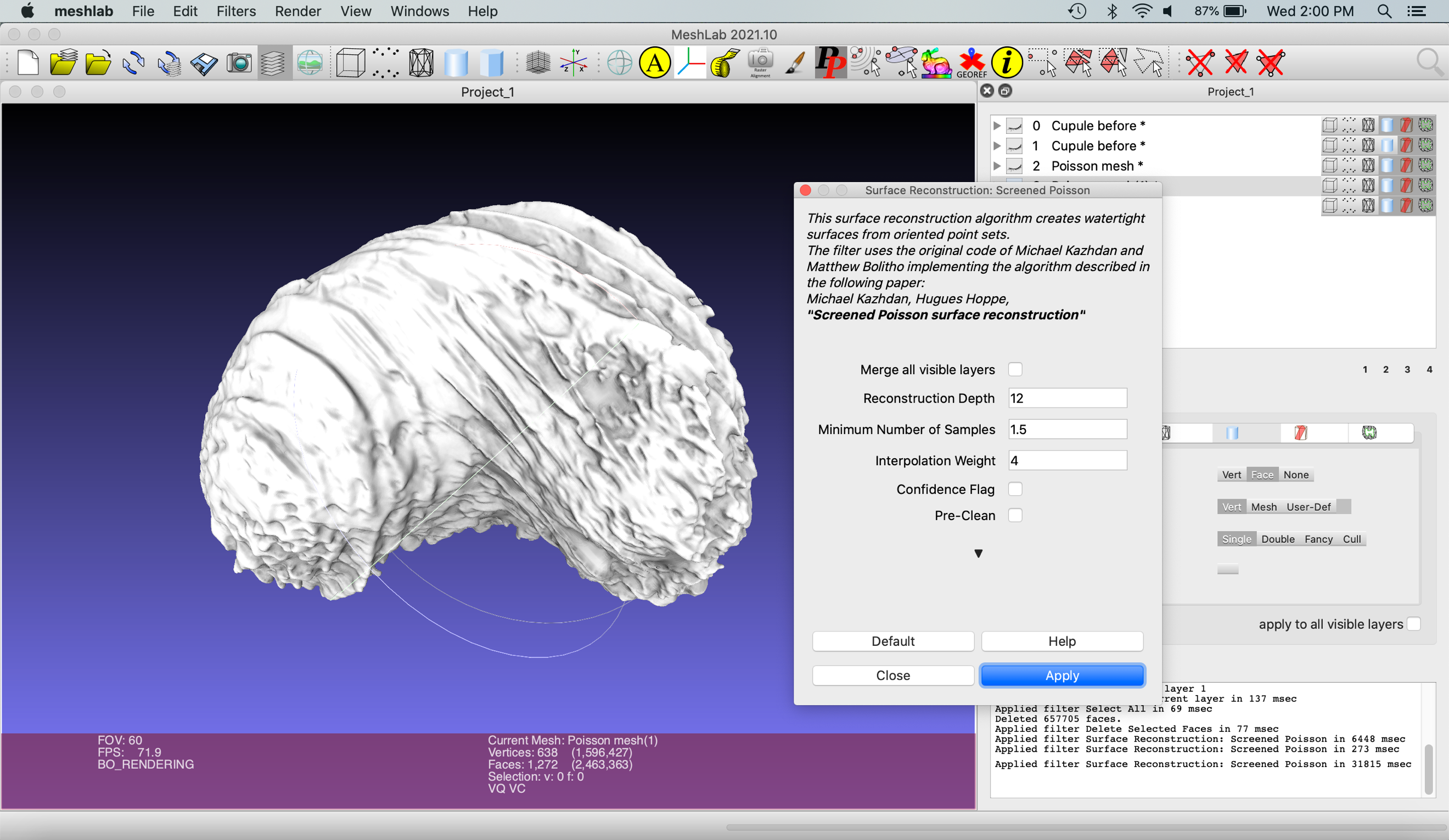

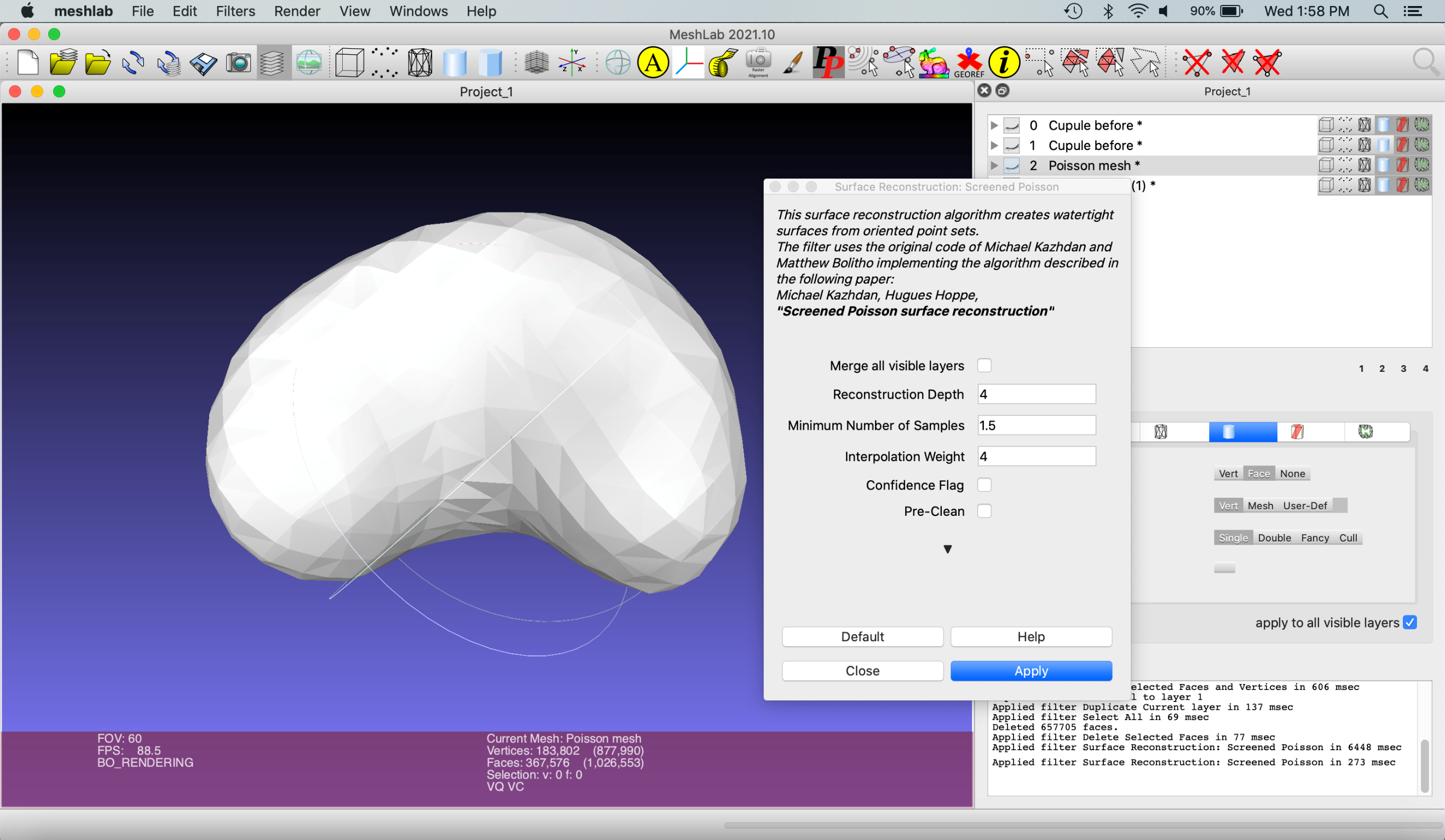


To rename a layer, **Right click the layer** > **Rename Current mesh** > **rename the mesh** > **Apply.** **Select the poisson mesh with the reconstruction depth of four and rename this mesh “Cupule remesh”**. It might seem counterintuitive to select the mesh with the lowest resolution. However, most of the ridges, bumps, and holes in the original mesh, captured by the poisson remeshes with reconstruction depth of 8 and 12, were preservational artifacts or consequences of CT segmentation. In certain cases, building a mesh that averages out these elements and captures the general shape of the object is appropriate.

The mesh is blocky due to the low number of polygons and relatively large size of each. There are a few ways to remedy this. First, apply another poisson remesh on top of the current poisson remesh. This time, set the Reconstruction Depth to 12. (**Filters** > **Remeshing, Simplification and Reconstruction** > **Surface Reconstruction: Screened Poisson** > **Reconstruction Depth = 12** > **Apply**).


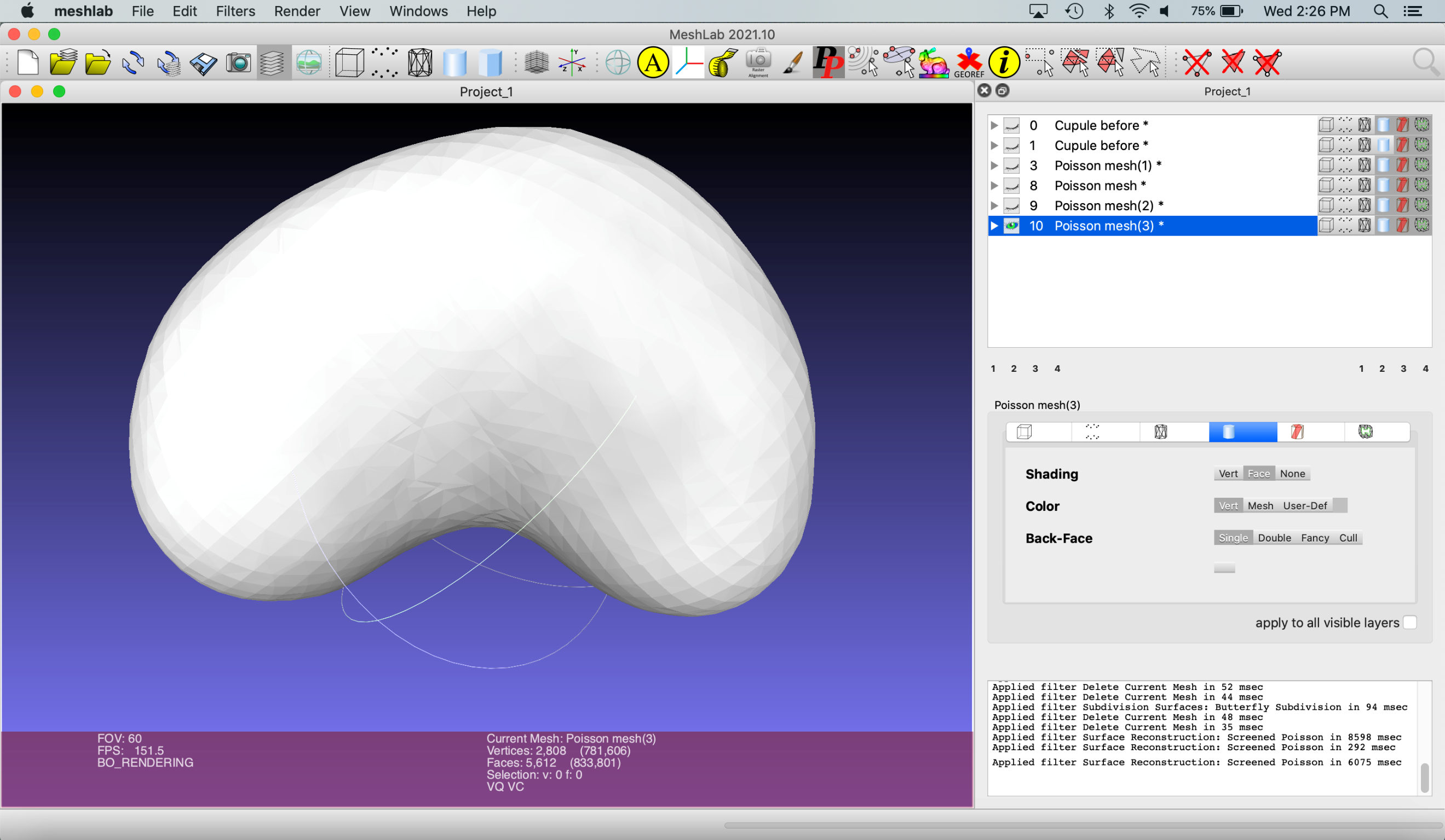


Next, select **Filters** > **Remeshing, Simplification and Reconstruction** > **Subdivision Surfaces: Butterfly Subdivision**. This filter divides the polygons to increase the number comprising the mesh. Each application makes the surface appear smoother, but also exponentially increases the file size. As such, the filter should be applied the minimum number of times to create a smooth surface. In this example, the filter was applied twice.


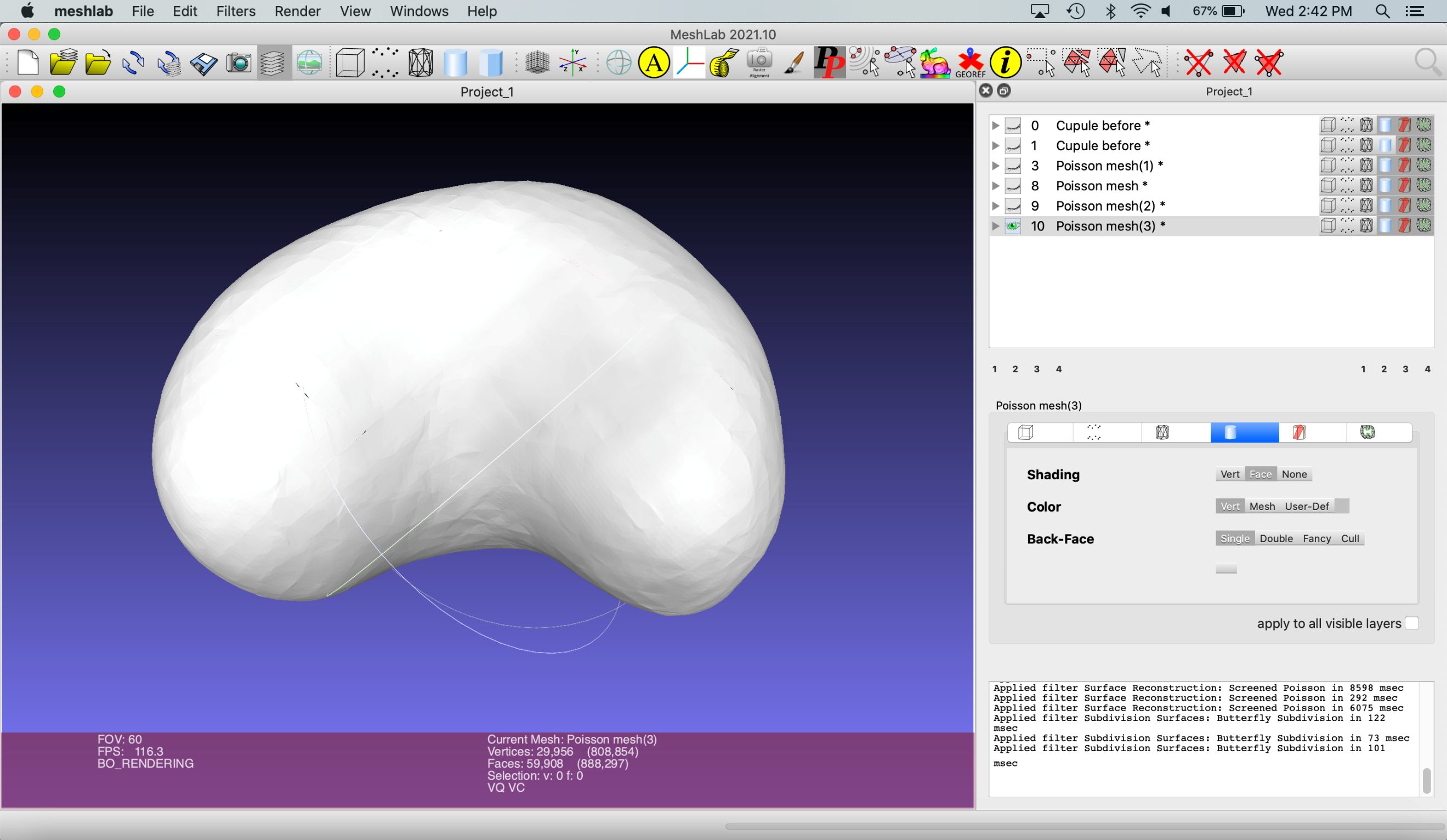


Once the reconstruction is complete, export the new mesh by selecting **File** > **Export Mesh**. Select .stl as the file type.


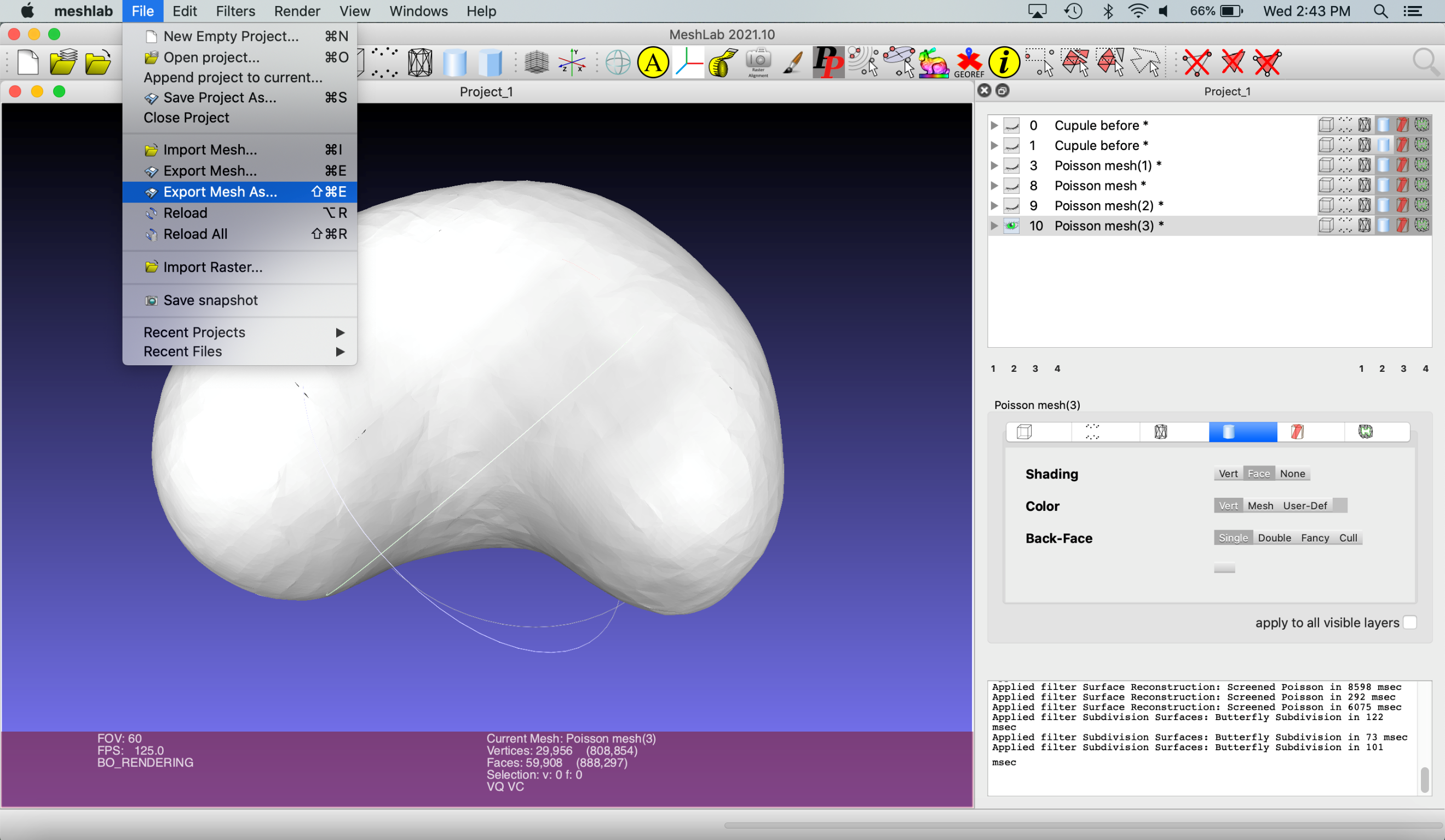


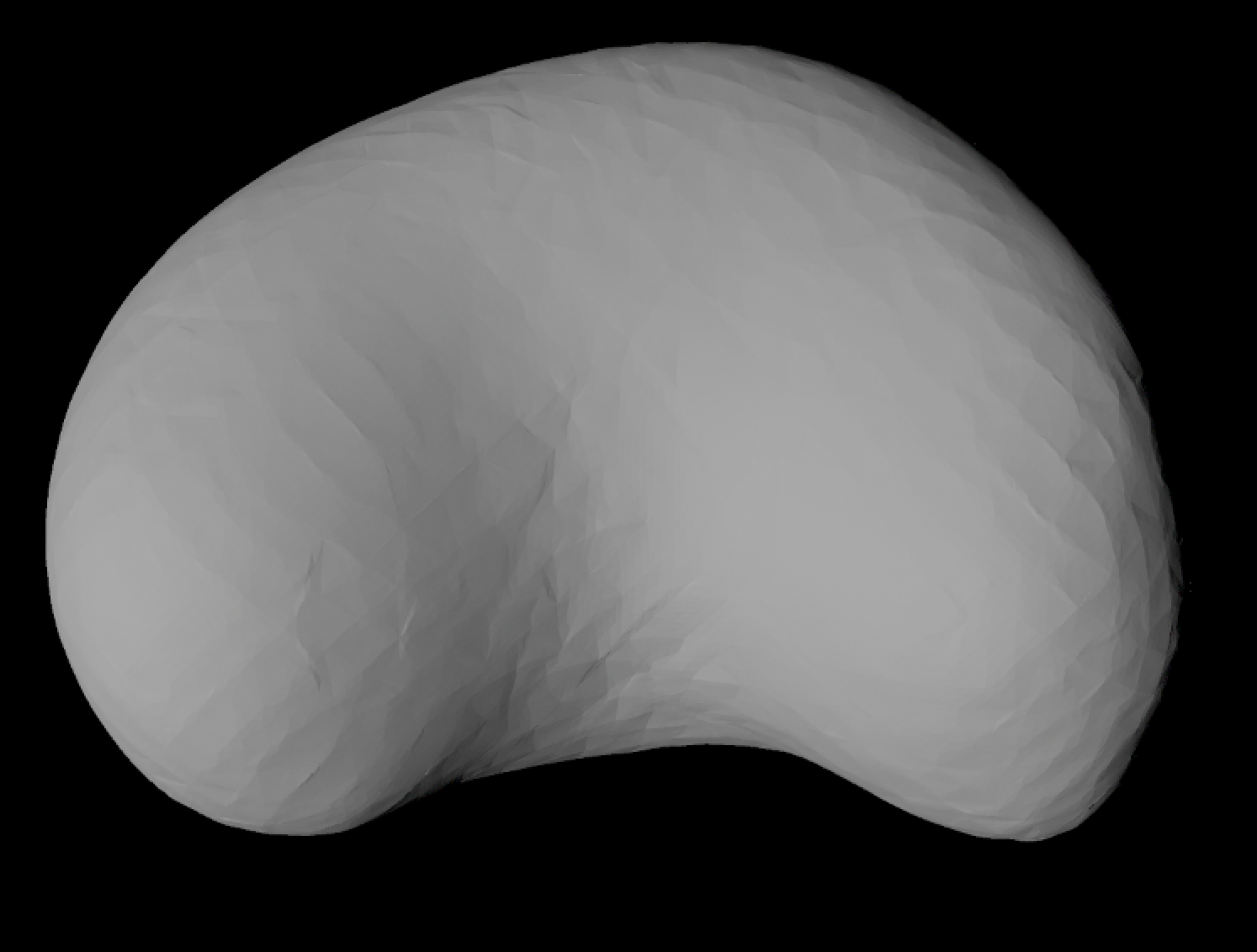


**References**

Cignoni, P., Callieri, M., Corsini, M., Dellepiane, M., Ganovelli, F., Ranzuglia, G. 2008. MeshLab: an Open-Source Mesh Processing Tool. Sixth Eurographics Italian Chapter Conference, page 129-136, 2008

Kazhdan, M. and Hoppe, H. 2013. Screened Poisson surface reconstruction. ACM Trans. Graphics, 32(3).

Shi, G., Herrera, F., Herendeen, P. S., Clark, E. G., Crane, P. R. 2021. Mesozoic cupules and the origin of the angiosperm second integument. *Nature* 594, 223-226.

**3D Mesh Cleanup Tutorial: Sphenodon (Intermediate)**

*Skills developed:* Basic mesh sculpting

Even some of the nicest fossil specimens may be affected by missing elements or broken features. Because these issues may be widespread in fossil specimens, this tutorial utilizes an extant specimen (*Sphenodon punctatus*, UF 11978, Florida Museum of Natural History) with only two issues for simplicity within the tutorial. In this example, a 3D mesh of *Sphenodon punctatus* was edited to create a hole in the dorsal portion of the skull and a missing post-orbital bar. These missing portions of the skull represent common taphonomic issues that affect vertebrate fossils as well as common breaks that occur in skeletonized extant specimens. The original mesh of *Sphenodon punctatus* was downloaded from Morphosource.org and is used under Creative Commons license: BY-NC Attribution-Noncommercial 4.0 International.


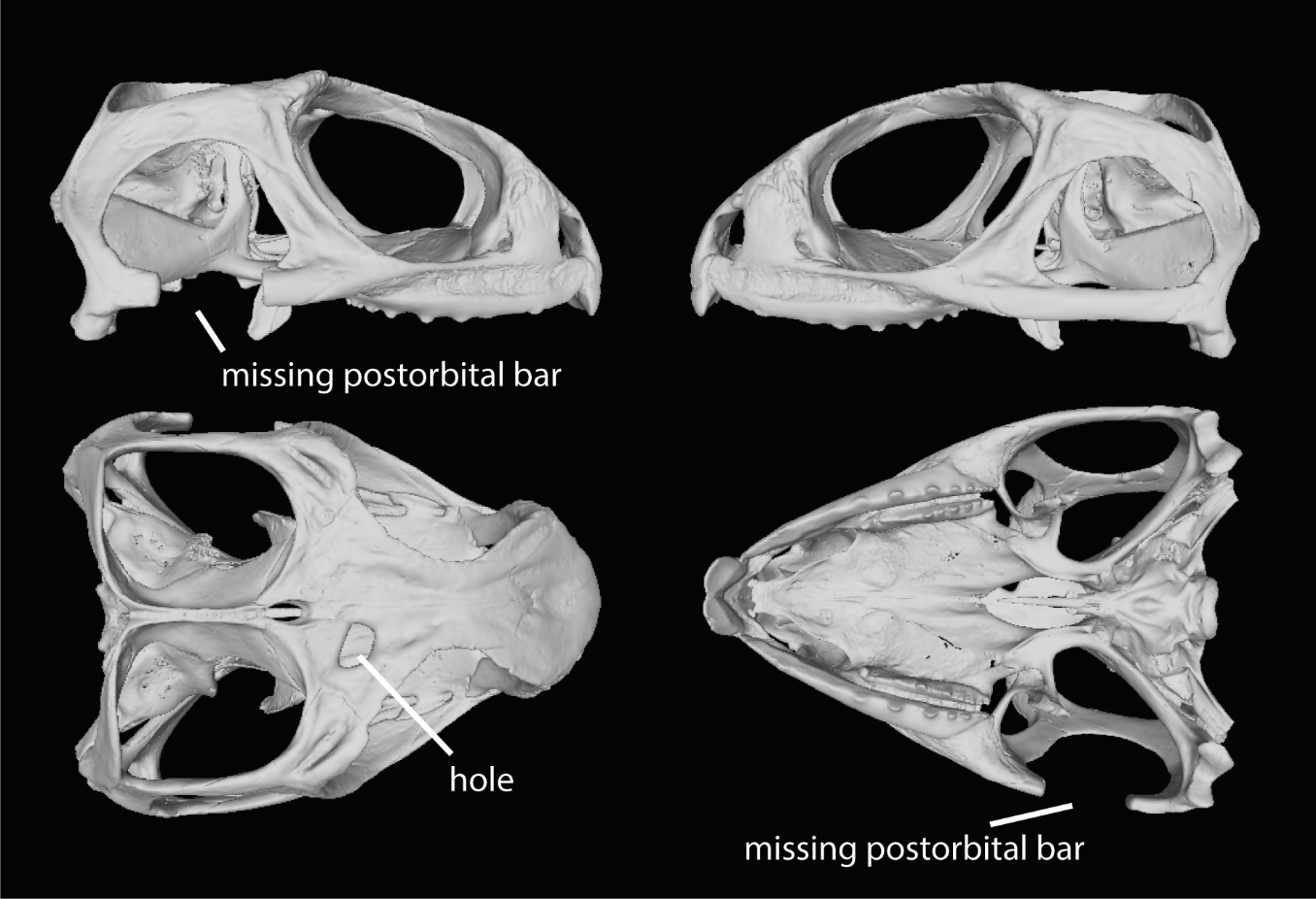


This tutorial will take place in two parts. The first is designed to teach basic mesh manipulation in Autodesk Maya by making simple shapes. The second is designed to teach fossil repair by sculpting a patch for the dorsally located hole in the *Sphenodon* skull and a new post orbital bar. Note that this tutorial utilizes Autodesk Maya 2020, though other versions will have similar layouts and functions. It is highly recommended to use a mouse to work through the tutorial, because a laptop trackpad may not have all the capabilities necessary to carry out Maya’s functions.

Part 1: Making and sculpting simple shapes in Autodesk Maya 2020

**Open Maya**. A new scene in Maya will appear similarly to the image below.


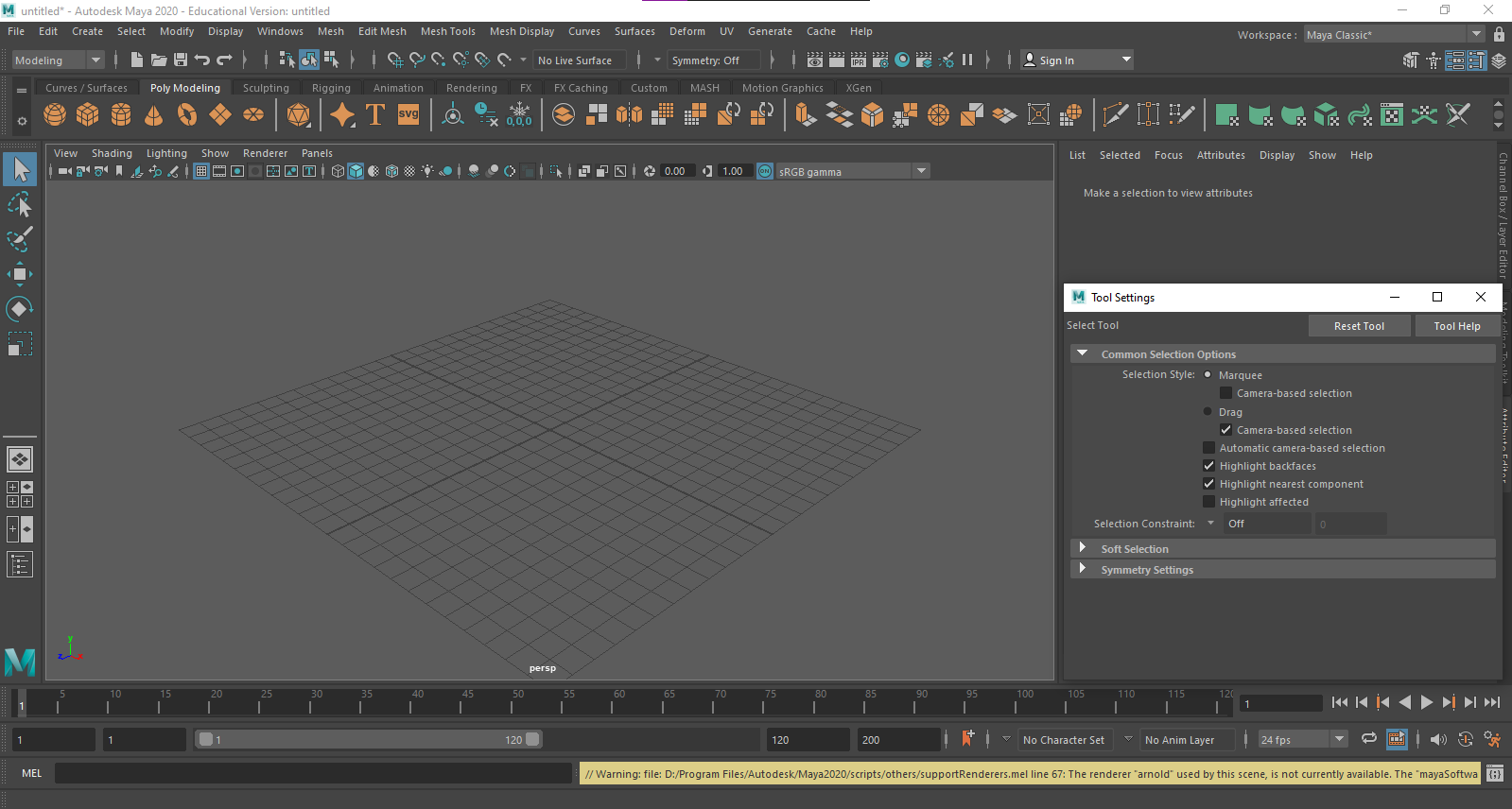


To begin making a simple shape, **select the cube from the “Poly Modeling”” tab**. When you click on the cube icon, a cube will appear in the center of the scene. You can also create a polygon by going to **Create > Polygon Primitive and selecting the cube.**


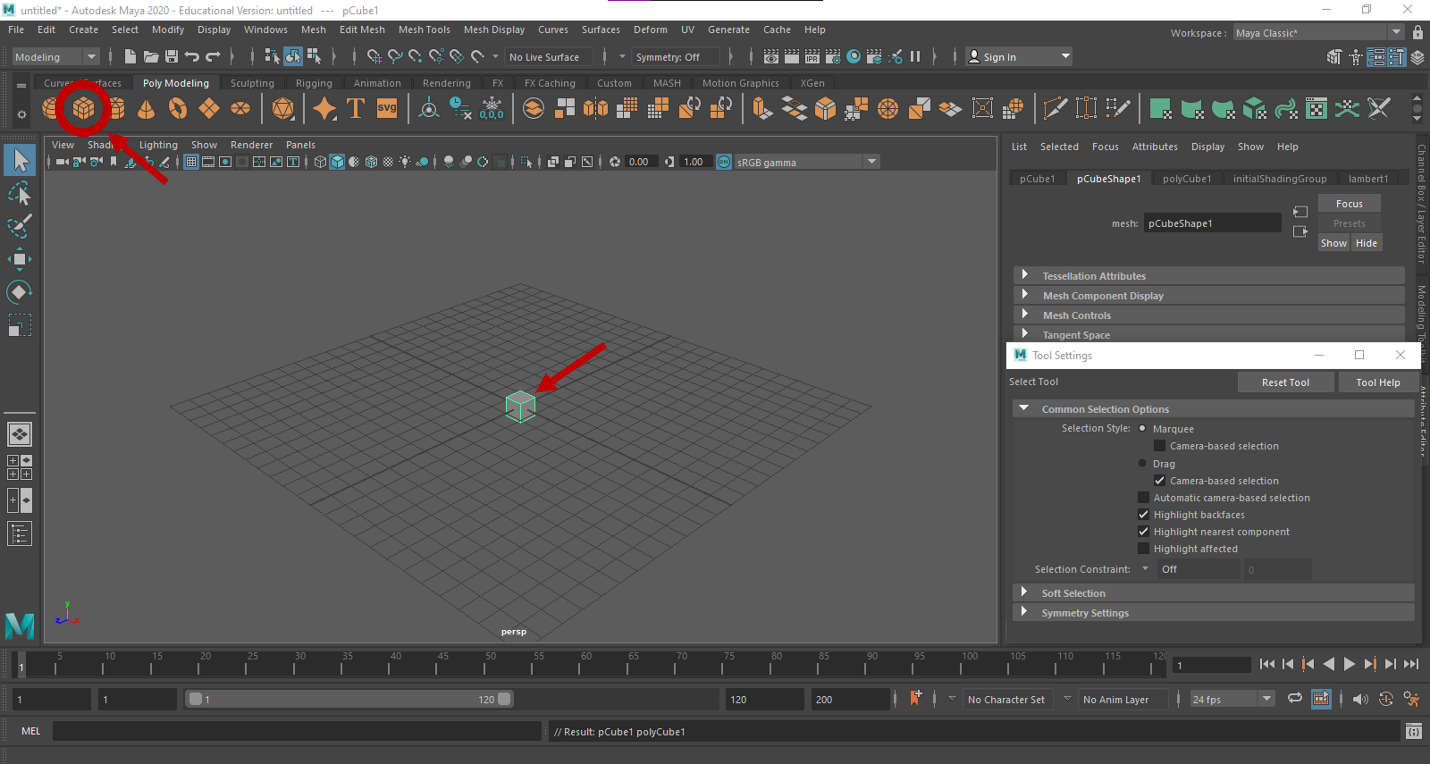


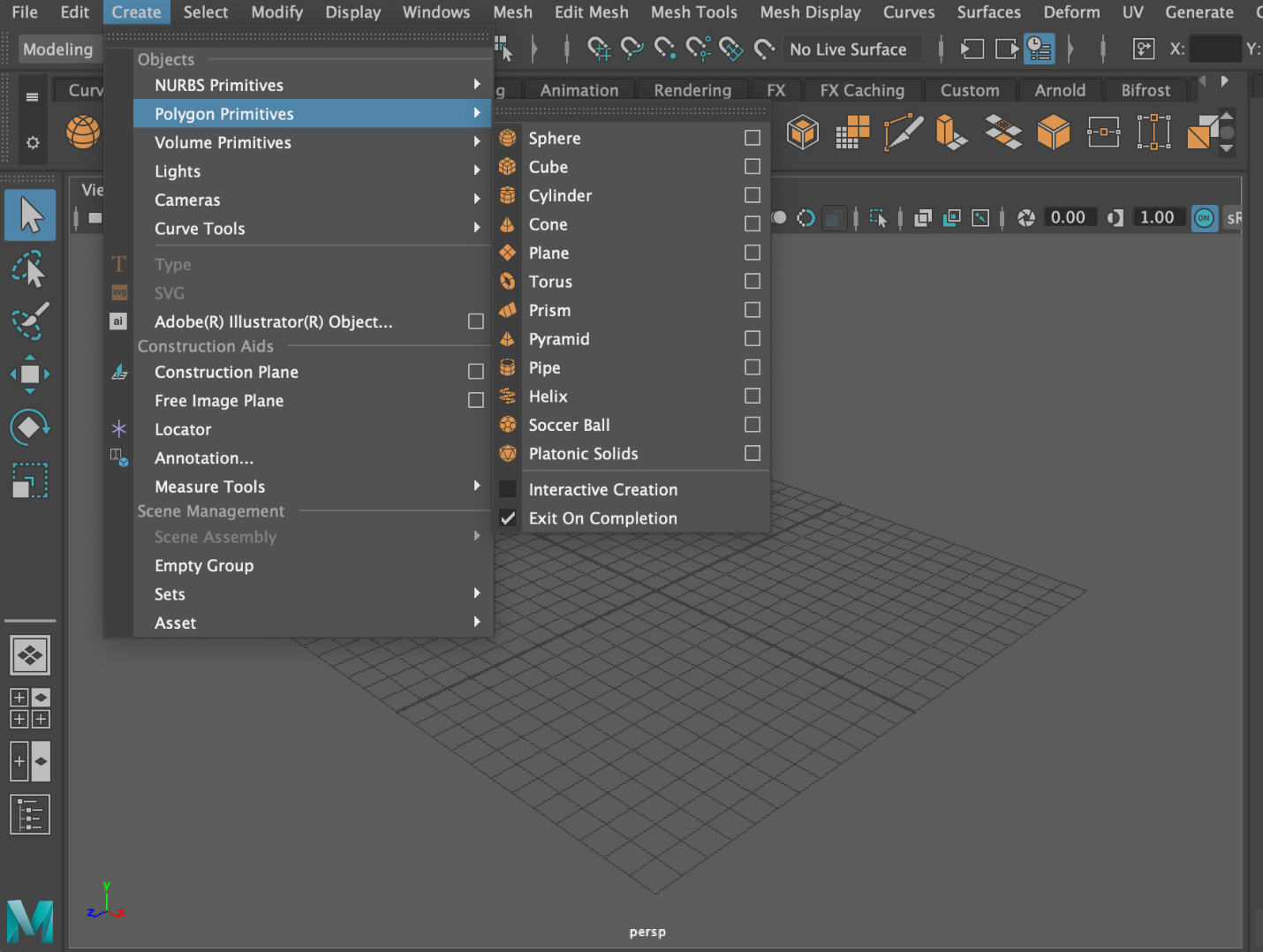


To zoom in on the scene, **scroll using the center scrolling button on the mouse** or **Alt + Right Mouse Button**. You can move around the scene using **Alt + Left Mouse Button** (tumbling, or circular movement around the scene) and **Alt + Center Scrolling Mouse Button** (maintains angle while panning view).

Integral to the sculping process are the translate, rotate, and scaling tools. Selecting any of these tools will toggle new icons to appear within the cube that will allow you to move, rotate, and resize the cube in the x, y, and z planes.


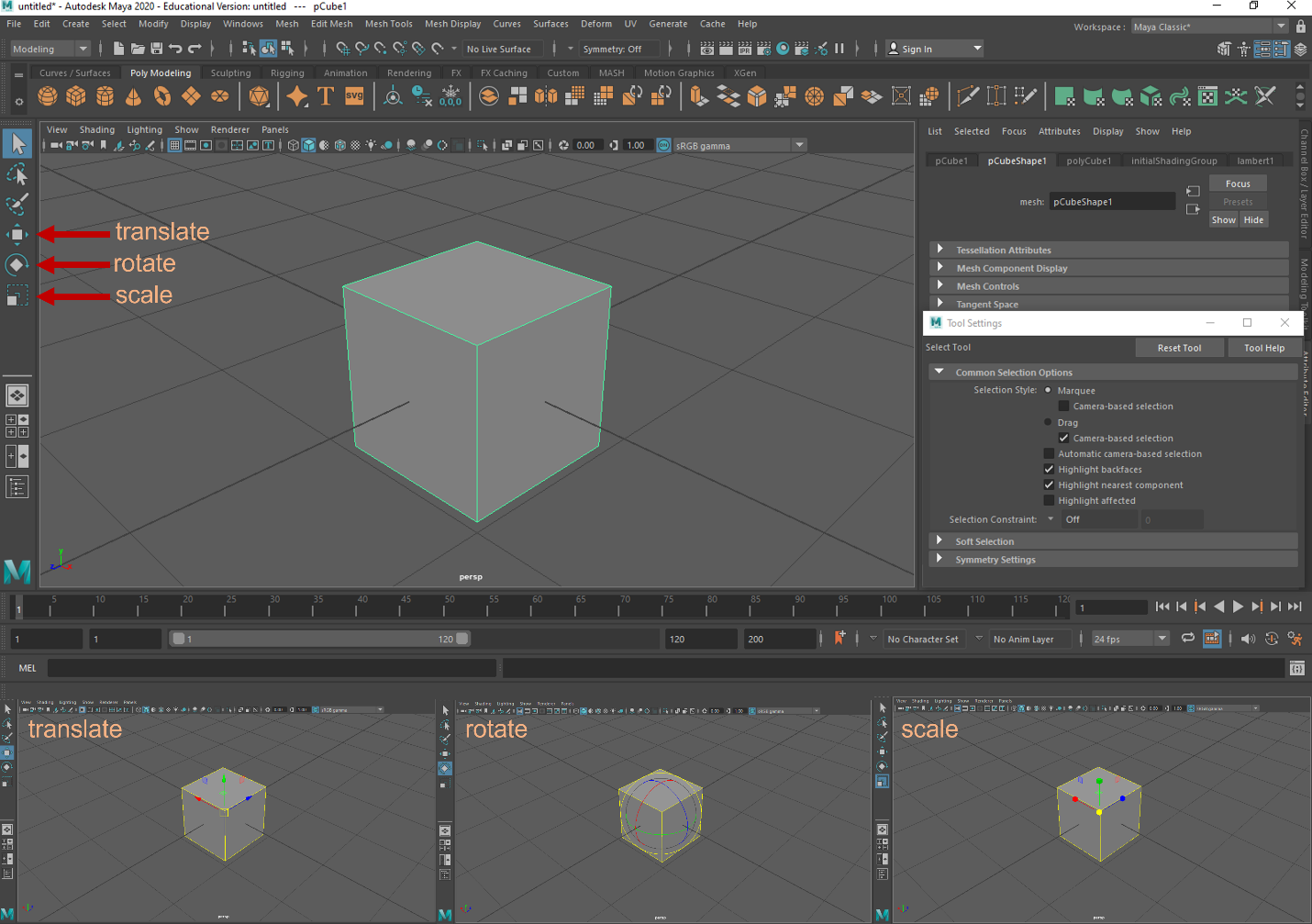


**Take a few minutes to practice these tools and change the size, shape, and position of the cube to look like the image below.**


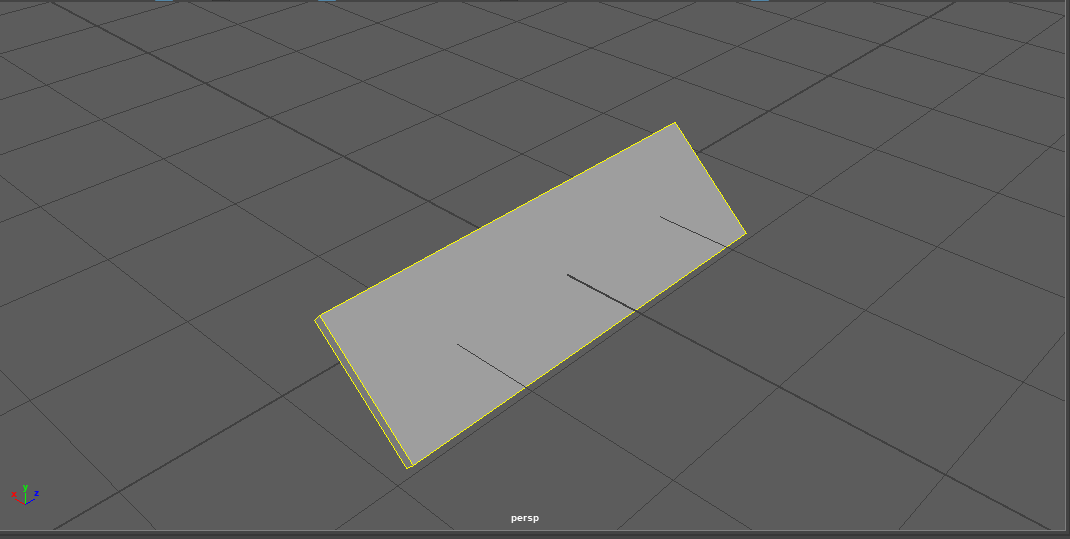


Once you have achieved this shape, you can delete it by **selecting Delete or Backspace.**

Now that you have interacted with the scaling tools, we will practice simple sculpting. Create a new cube again by **selecting the cube from the “Poly Modeling” tab**. Then, scale the cube so that it is thinner in one axis and equidistant in the other two axes, as in the image below.


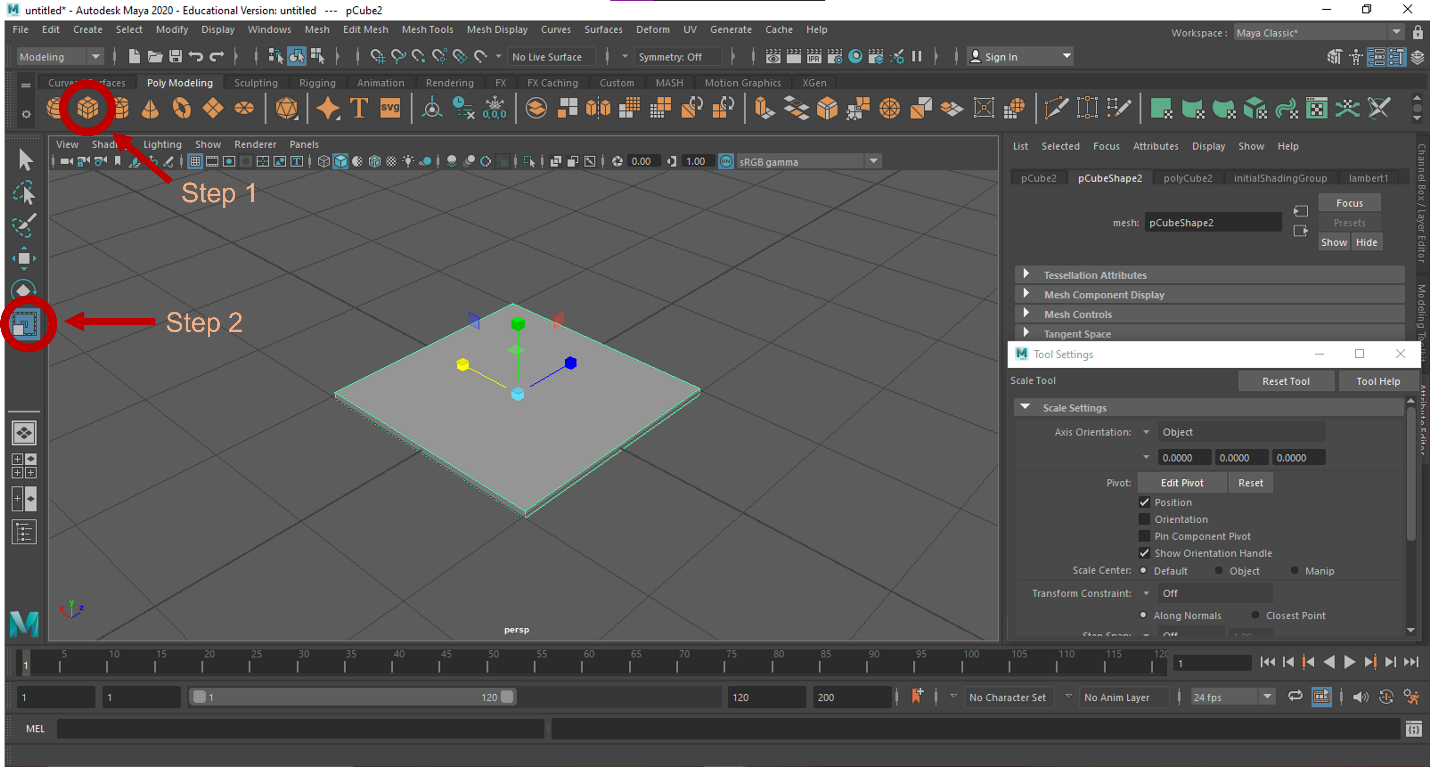


To make a simple shape, such as the cube, more malleable for digital sculpting, subdivisions can be added to the cube. **With the cube selected, navigate to the polyCube tab of the Attribute Editor**. **Add subdivisions to the Width, Height, and Depth by changing the value of each to 10**. Notice where these subdivisions appear on the cube as you edit each.


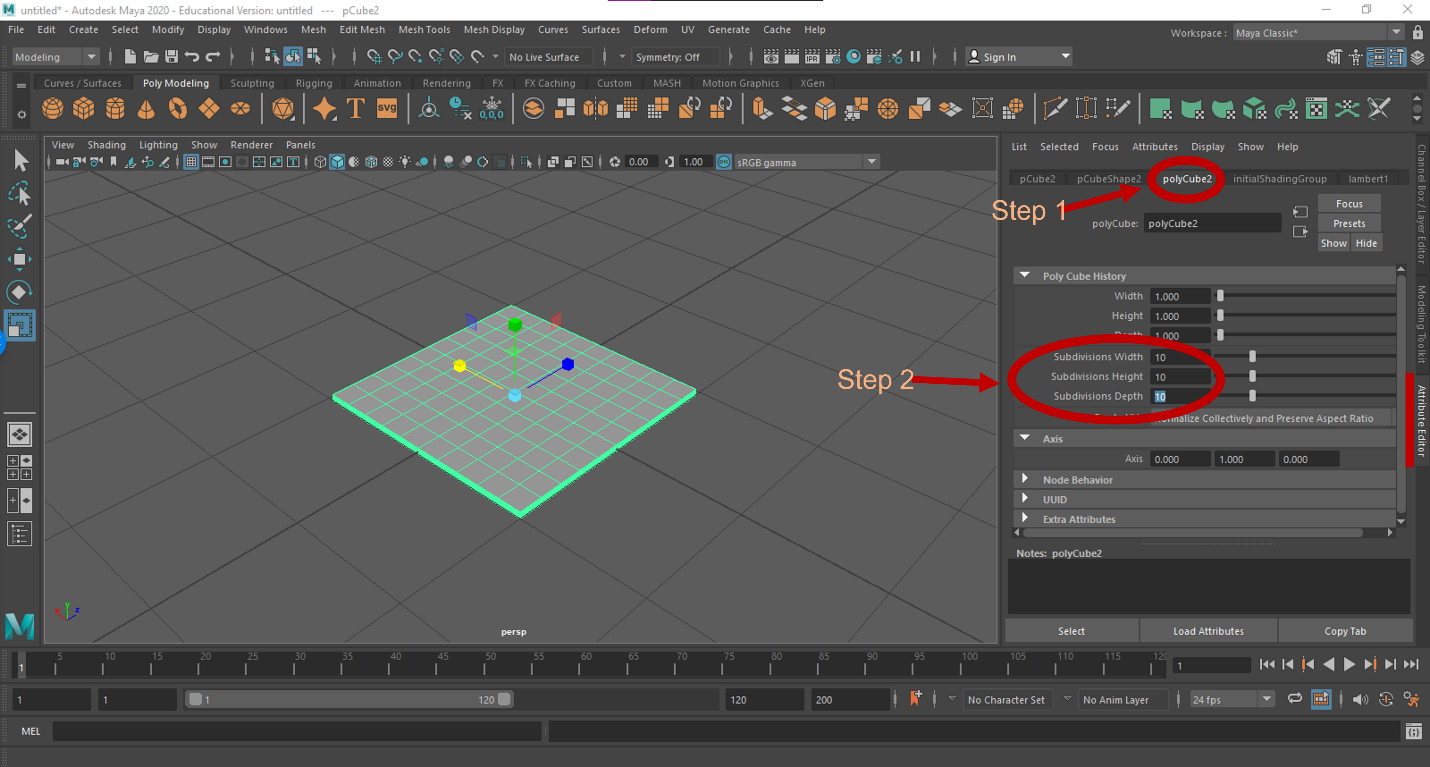


In order to manipulate the new faces created by these subdivisions, **hold the right mouse button, and then select Edge from the new pop-up menu that appears**.


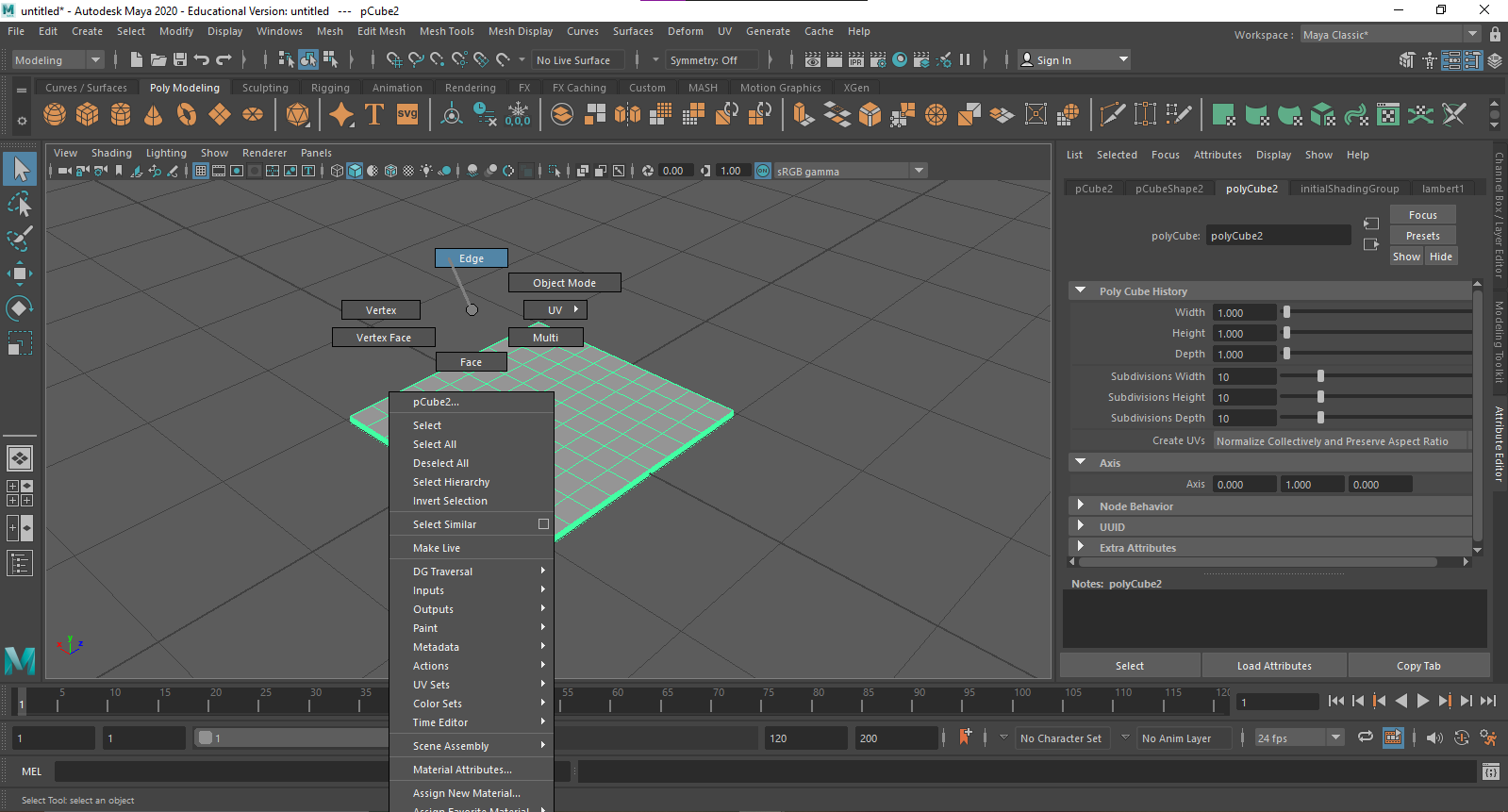


Now that you are in Edge Mode, **hover over the cube**. You will notice that a radius of edges is highlighted. As you sculpt objects in Maya, you may want to decrease or increase the size of the radius to affect fewer or more edges. **To do this hold B + Left Mouse Button**.


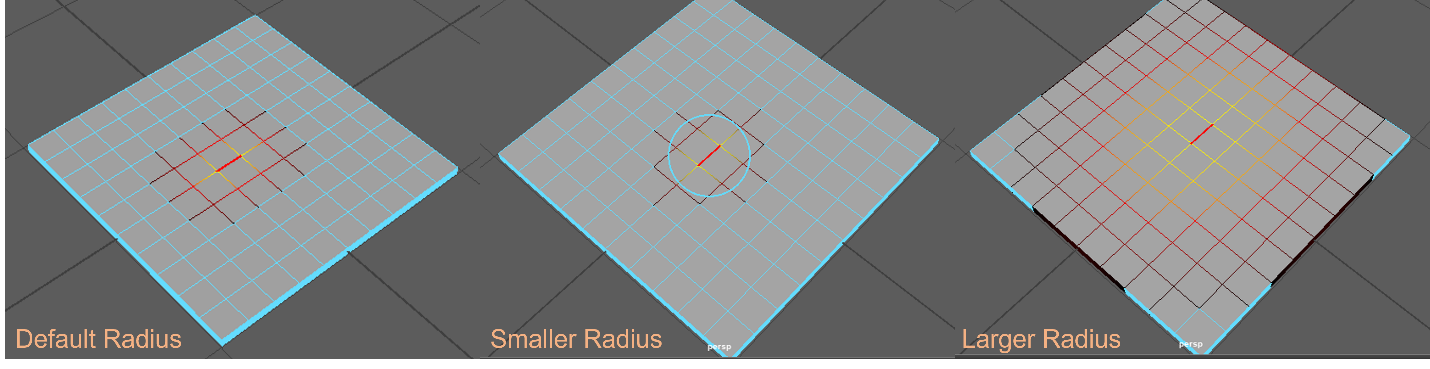


Now that you have played with these tools a bit, let us begin sculpting the cube mesh into a new shape. **Select an edge towards the center of the cube**. To manipulate this edge and the affected radius, the translate, rotate, and scaling tools can be used. **Select the translate tool, and then click and drag one of the axis arrows** to see how it affects the mesh. To practice, **try selecting different portions of the mesh and manipulate the mesh with the translate, rotate, and scaling tools.** You can also select an entire row of edges by **double clicking on a single edge within that row.**


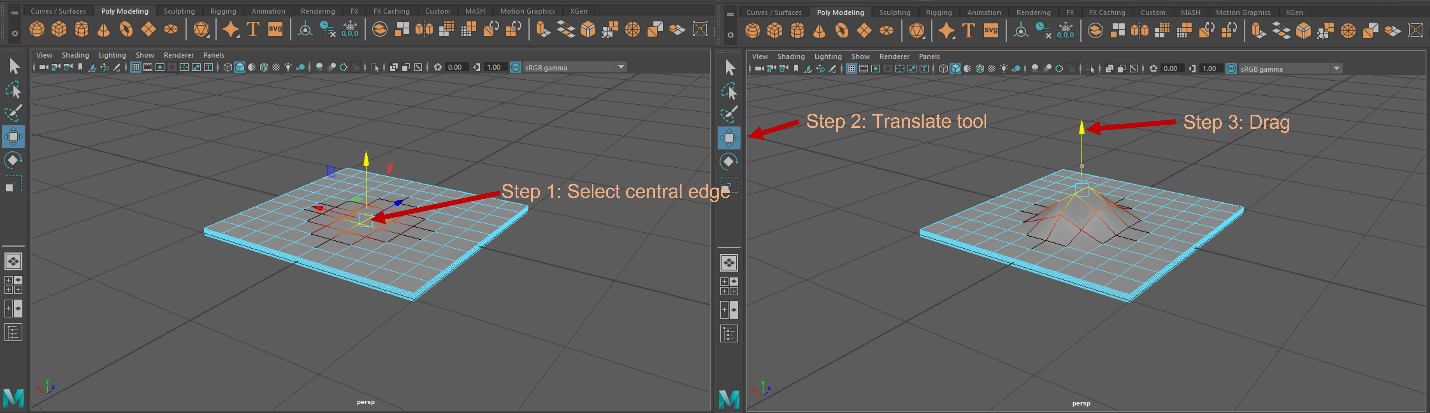


We suggest practicing mesh manipulation on a simple cube before progressing to Part 2 (fossil/bone reconstruction). Try transforming the cube into different shapes such as a heart, clover, or tube if you would like extra practice.

Part 2: Reconstruction using simple shapes (i.e., Simple-Patch Method)

Import “Sphenodon with holes.stl” by **selecting File > Import.**


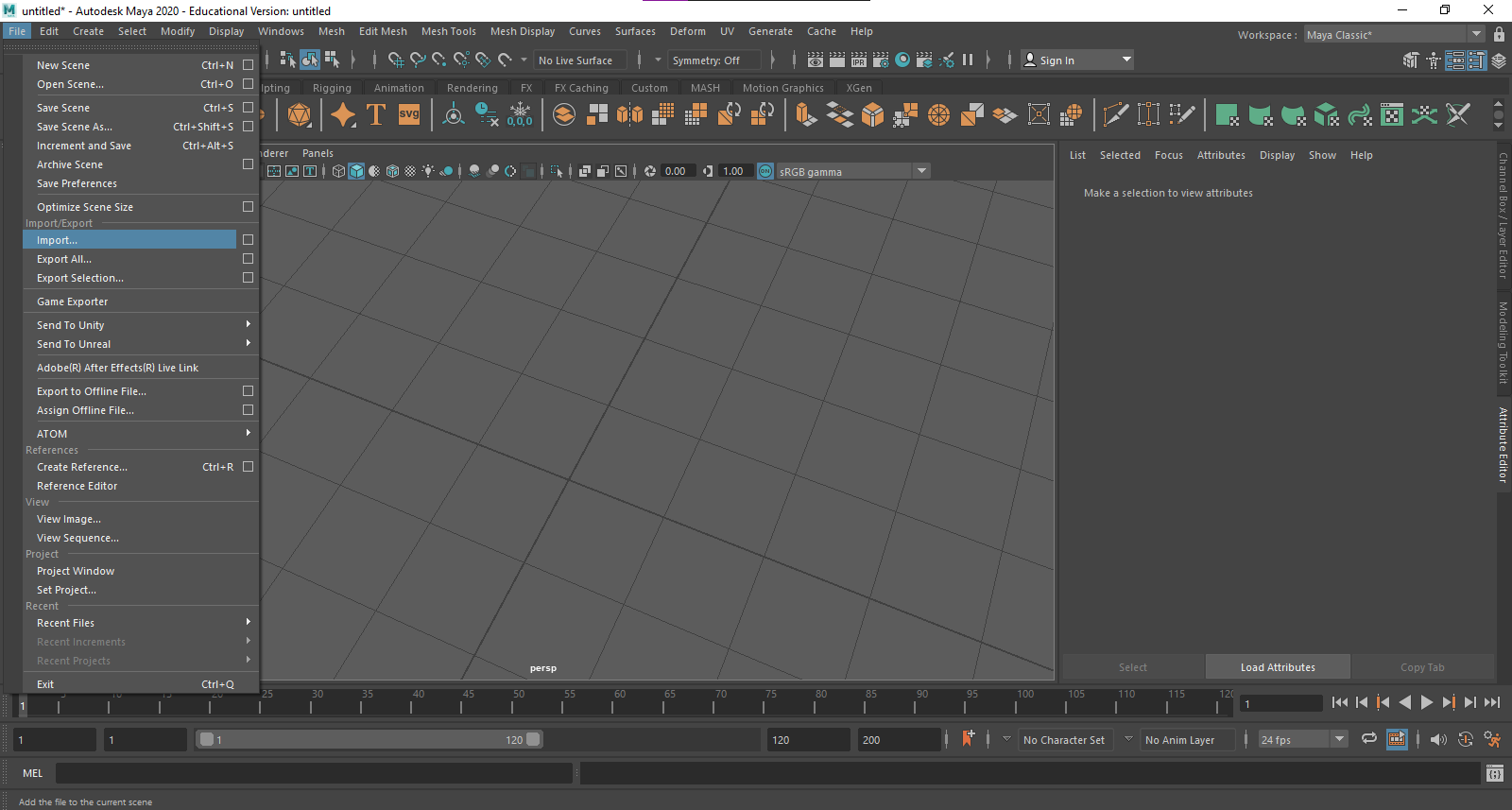


The mesh of the skull is somewhat larger than the reference grid in the Maya scene. If you find the grid cumbersome, the grid can be toggled on and off by **selecting the grid icon**. Note that whether or not the grid is visible, new shapes will always appear in the center of the grid (“the origin”).


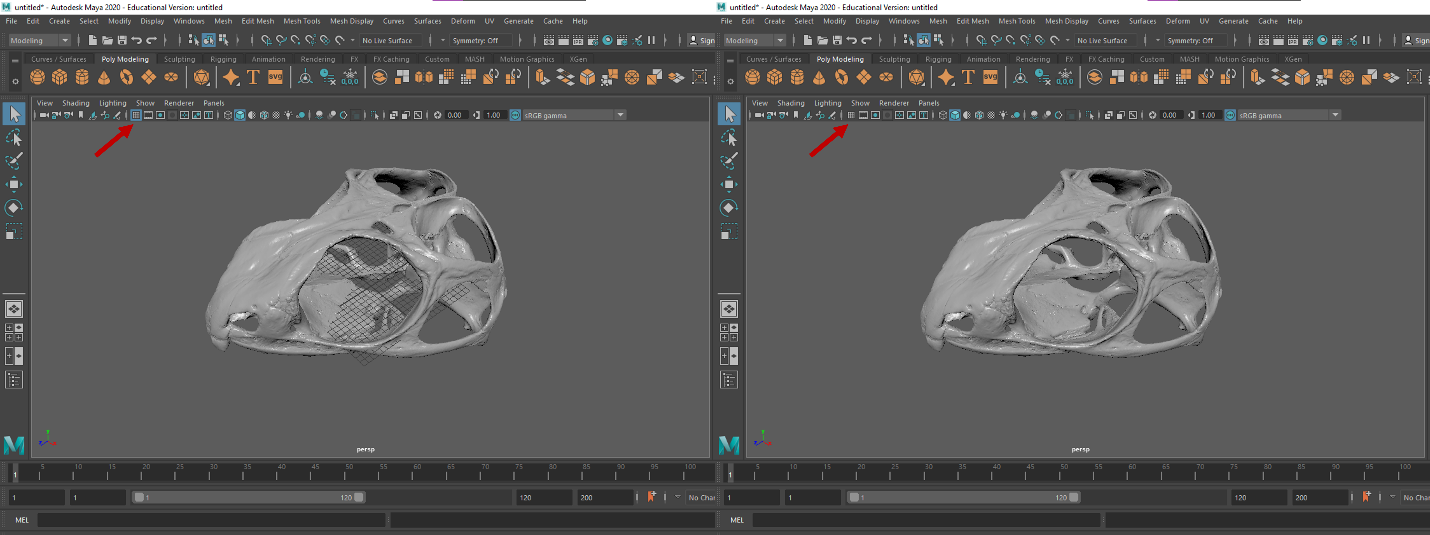


**Select the cube icon from the Poly Modeling tab**. Notice the cube appears in the center of the scene, within the *Sphenodon* skull.
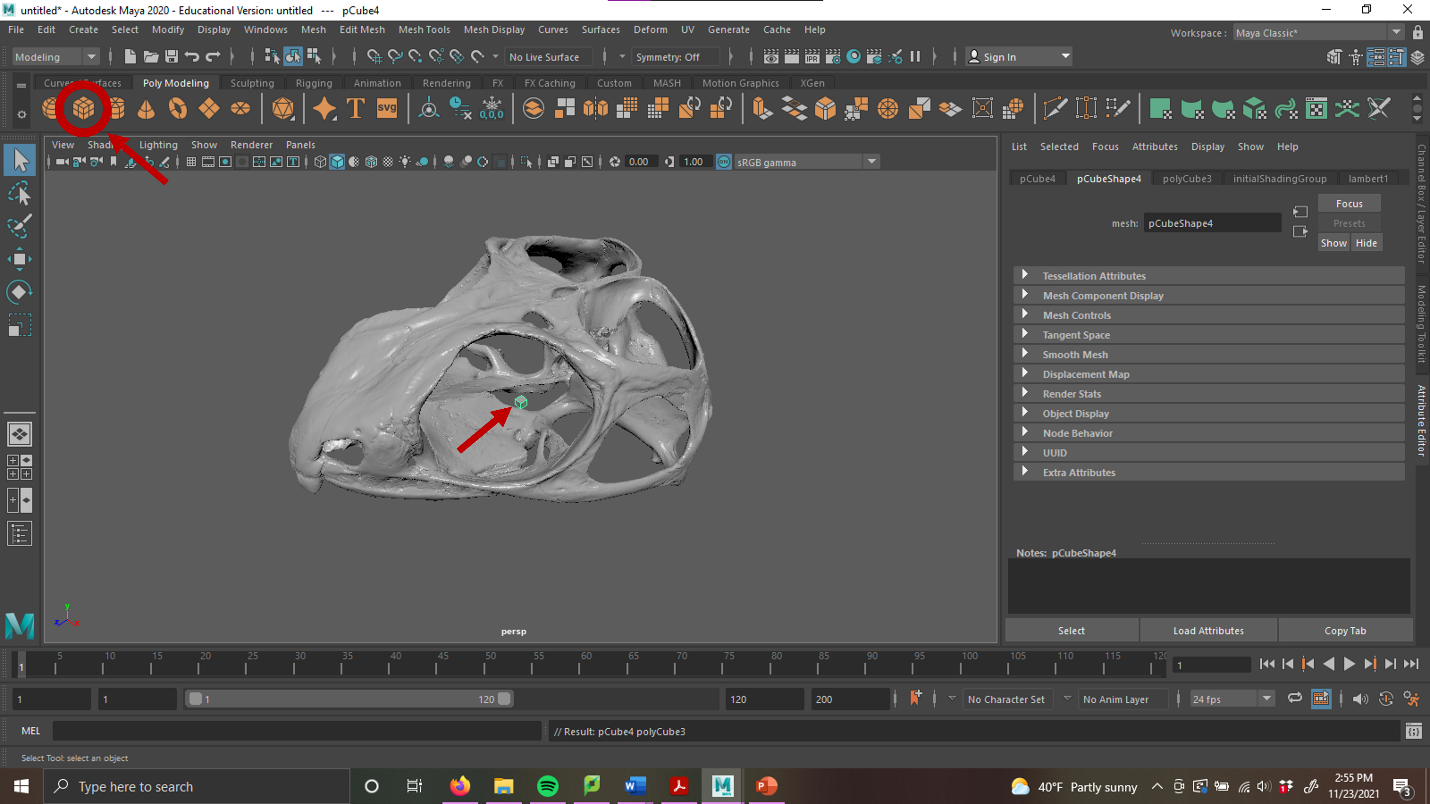


**Using the translate tool, move the cube so that it centered within the hole being fixed.** You may find it helpful to scroll around the scene to make sure the cube is aligned with the hole as closely as possible.


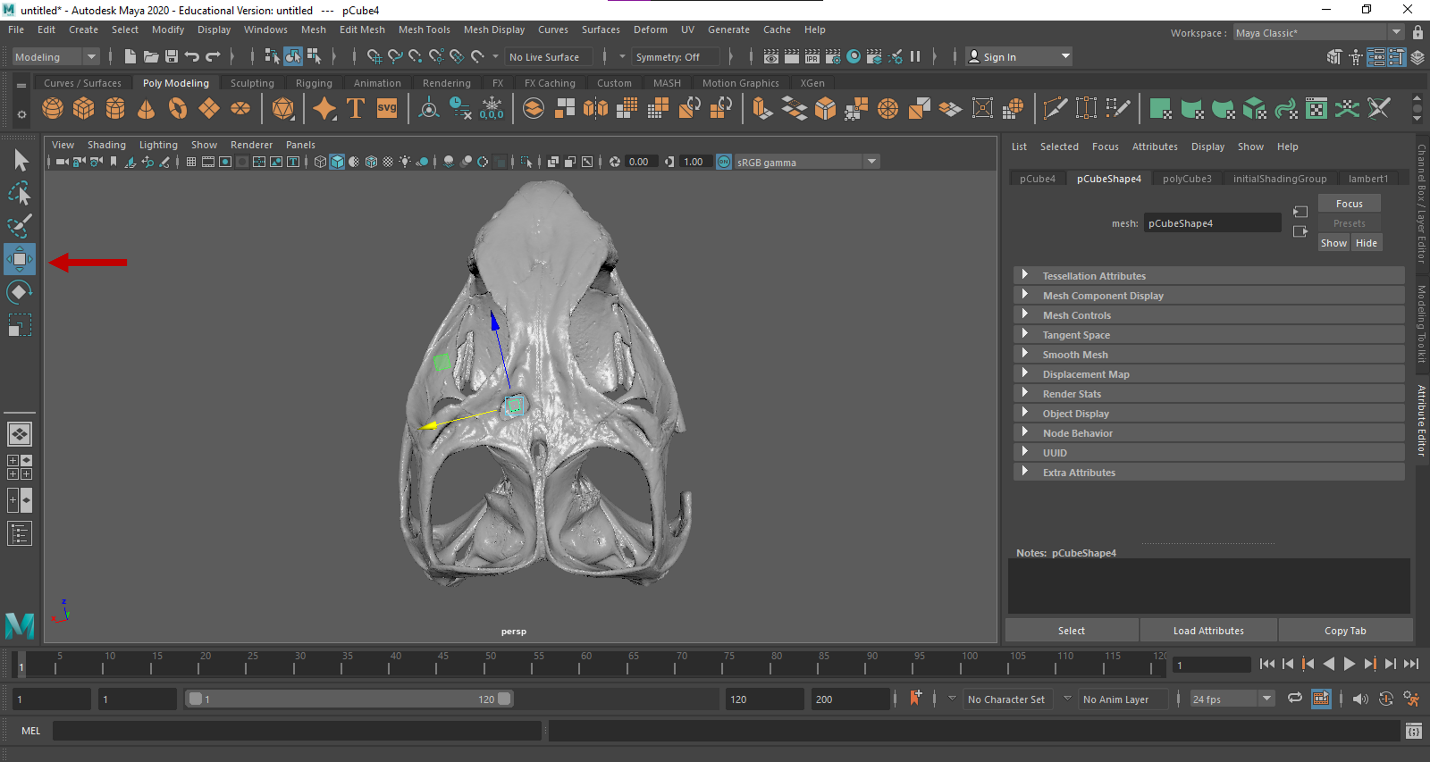


**Using the scale tool, size the cube so that it approximately fits the size of the hole.** You may also find it useful to fine-tune the placement of the cube using the rotate and translate tools.


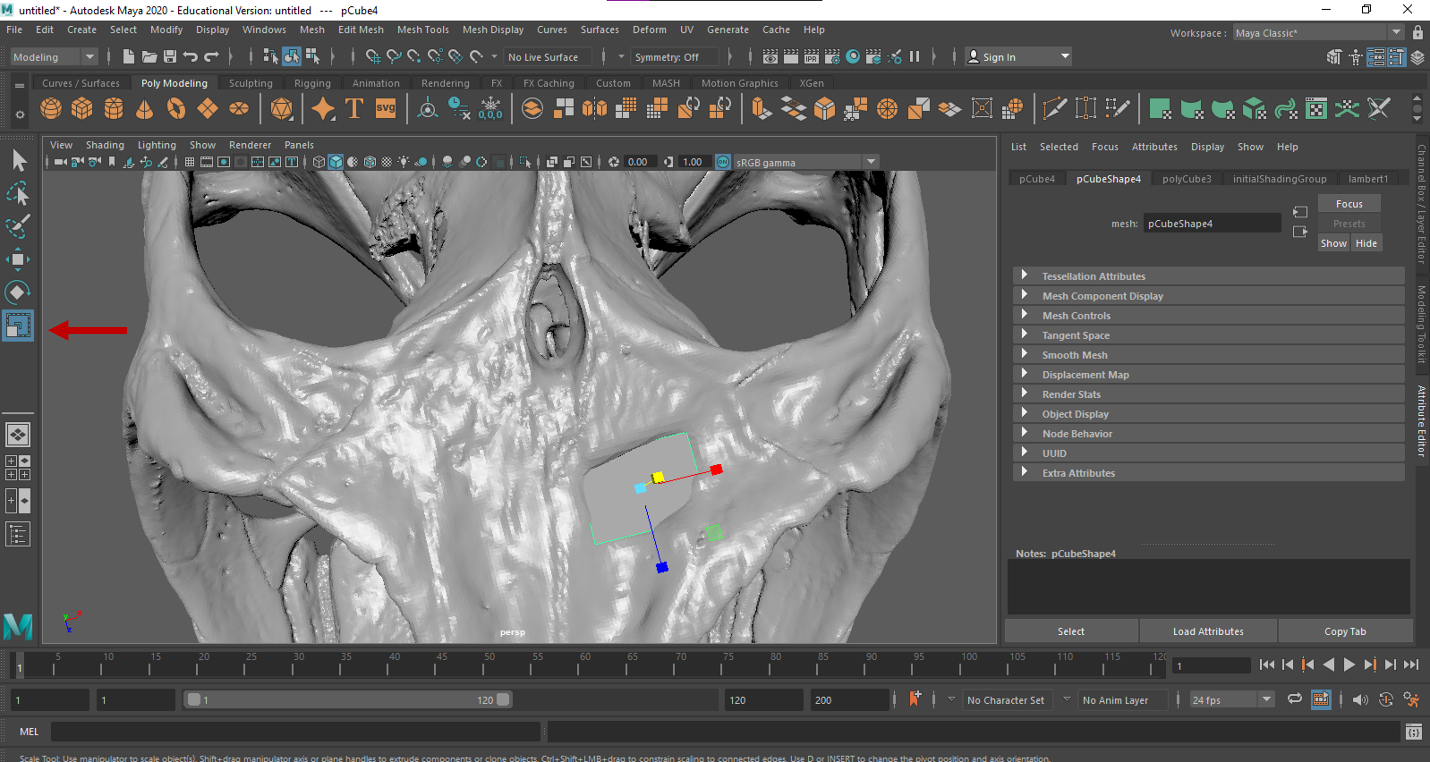


**Add subdivisions to the cube by** **selecting the polycube tab from the Attribute editor**. It is recommended to have more subdivisions for larger areas, and fewer subdivisions in smaller areas. In this example, there are 10 subdivisions for width, 3 for height, and 10 for depth.


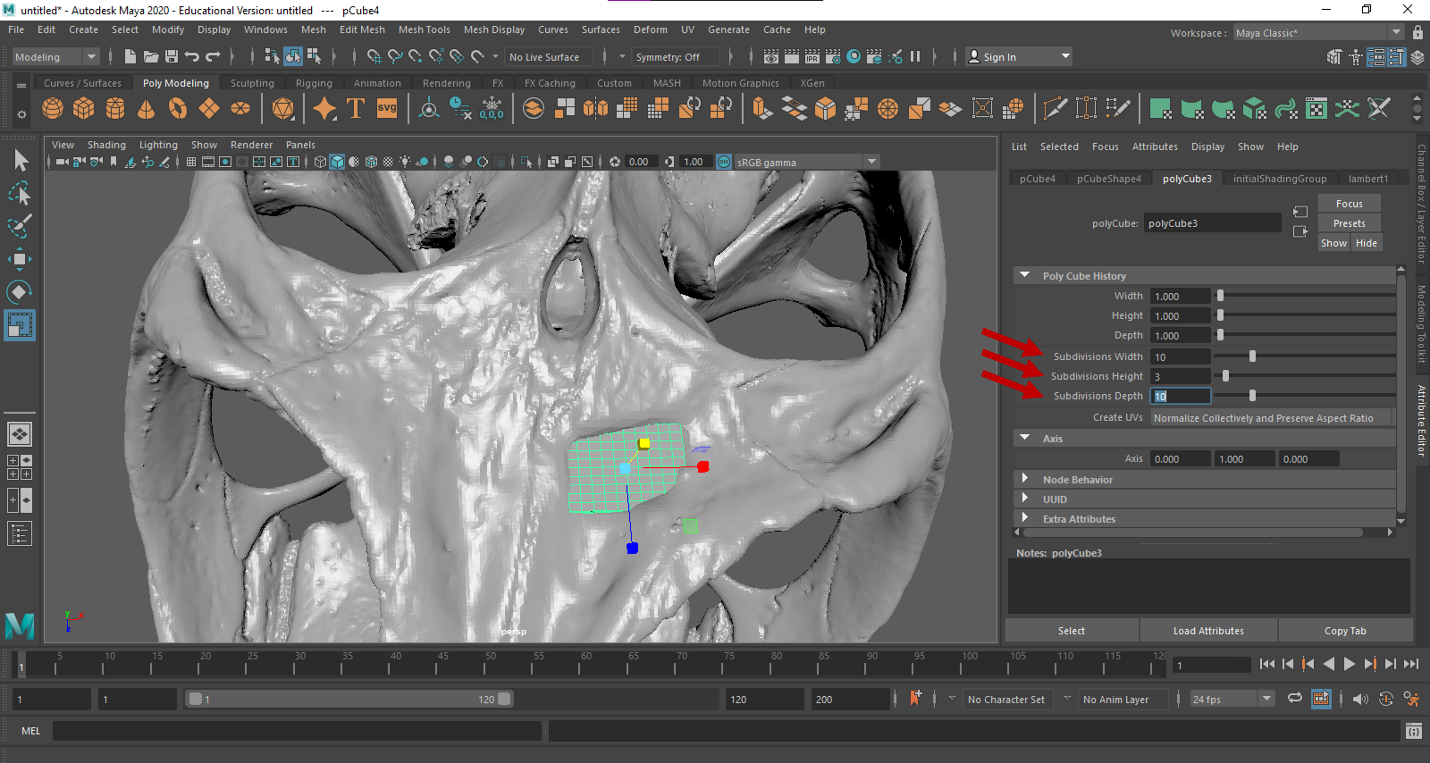


Now, switch to edge mode by **holding the right mouse button > Edge**. Now, the cube can be manipulated by its edges to match its surroundings using the scale, rotate, and translate tools as outlined in part one.

Sculpting is an individual process, and some users may be more artistically inclined than others. Depending on what you plan to use the model for (3D print vs. publication figures vs. biomechanical modeling), you may want to spend more or less time achieving as close to an anatomically correct model as possible. With that said, there are a few recommendations to make the patch look more streamlined with the original mesh.

First, **bury the edges of the patch within the original mesh**. In example below, this was done by translating edges of the patch and dragging them within the borders of the skull.


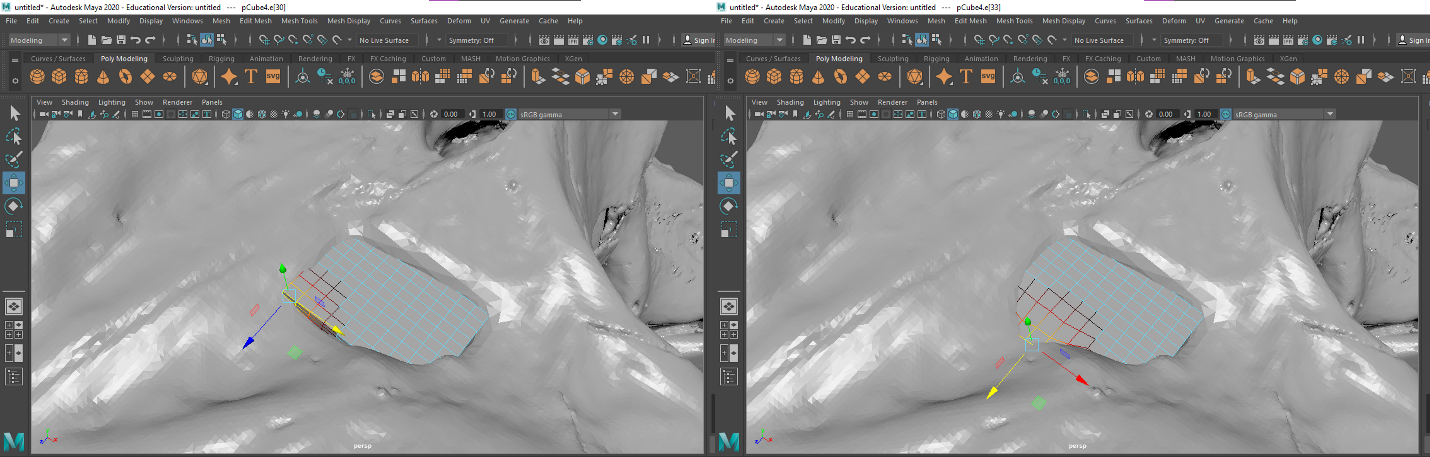


Second, **use the translate tool so that sunken areas are flushed with the original model**. This can be done by raising and lowering areas of the mesh to align with the morphology of the original model.


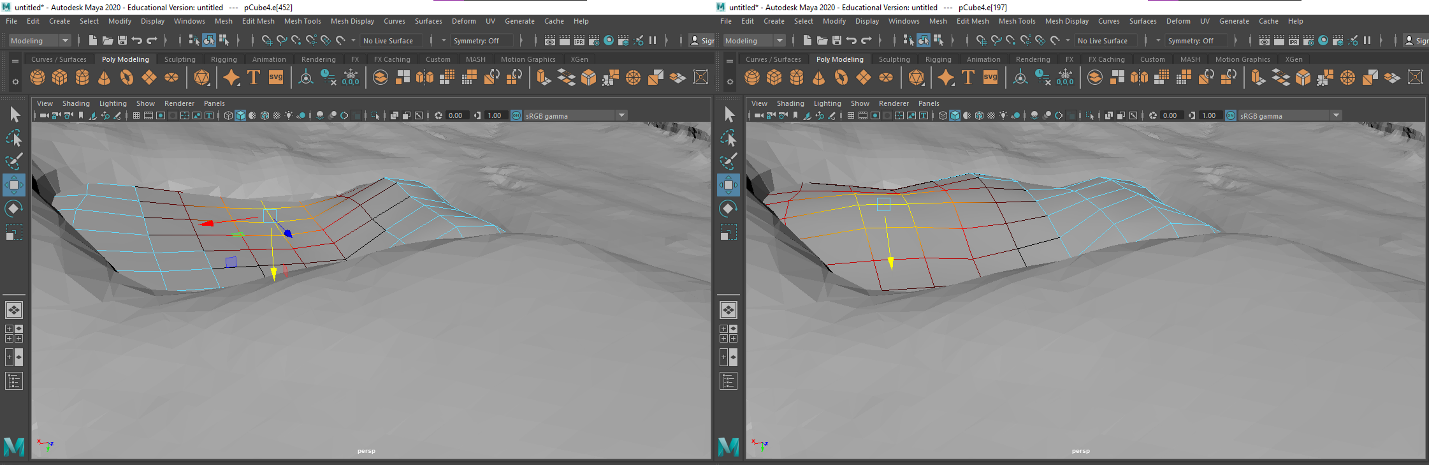


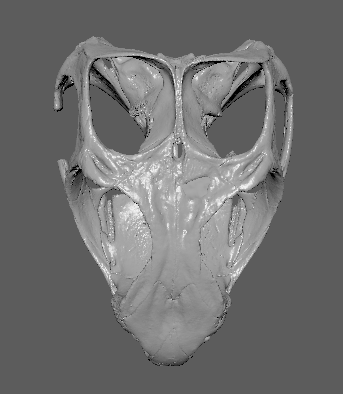


Next, repair the missing postorbital bar. This time, **select the cylinder from the Poly Modeling tab**. Note, it also appears in the center of the scene.


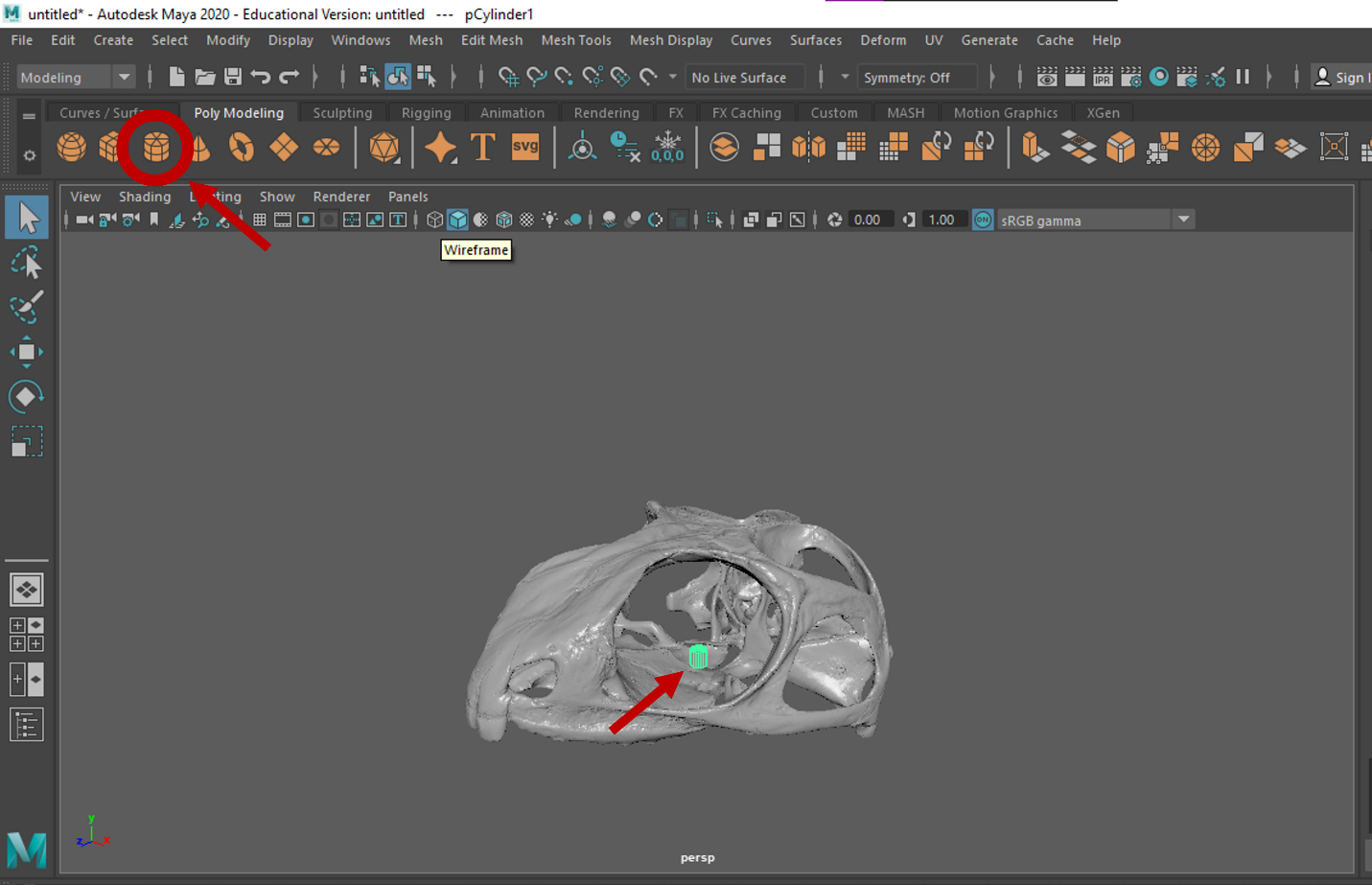


**Using the translate and rotate tools, align the cylinder with the post orbital bar.**


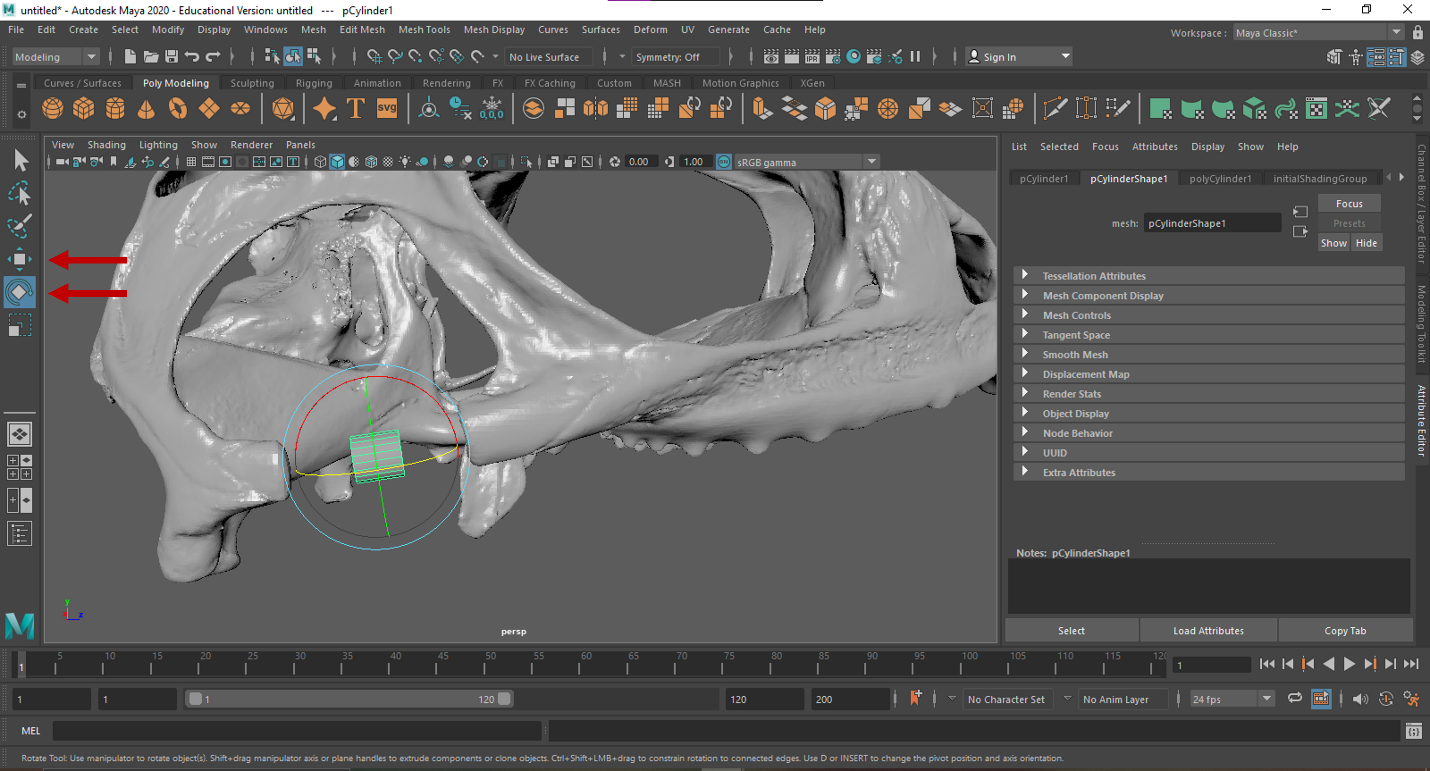


**Lengthen the cylinder in the direction of the post orbital bar using the scale tool.**


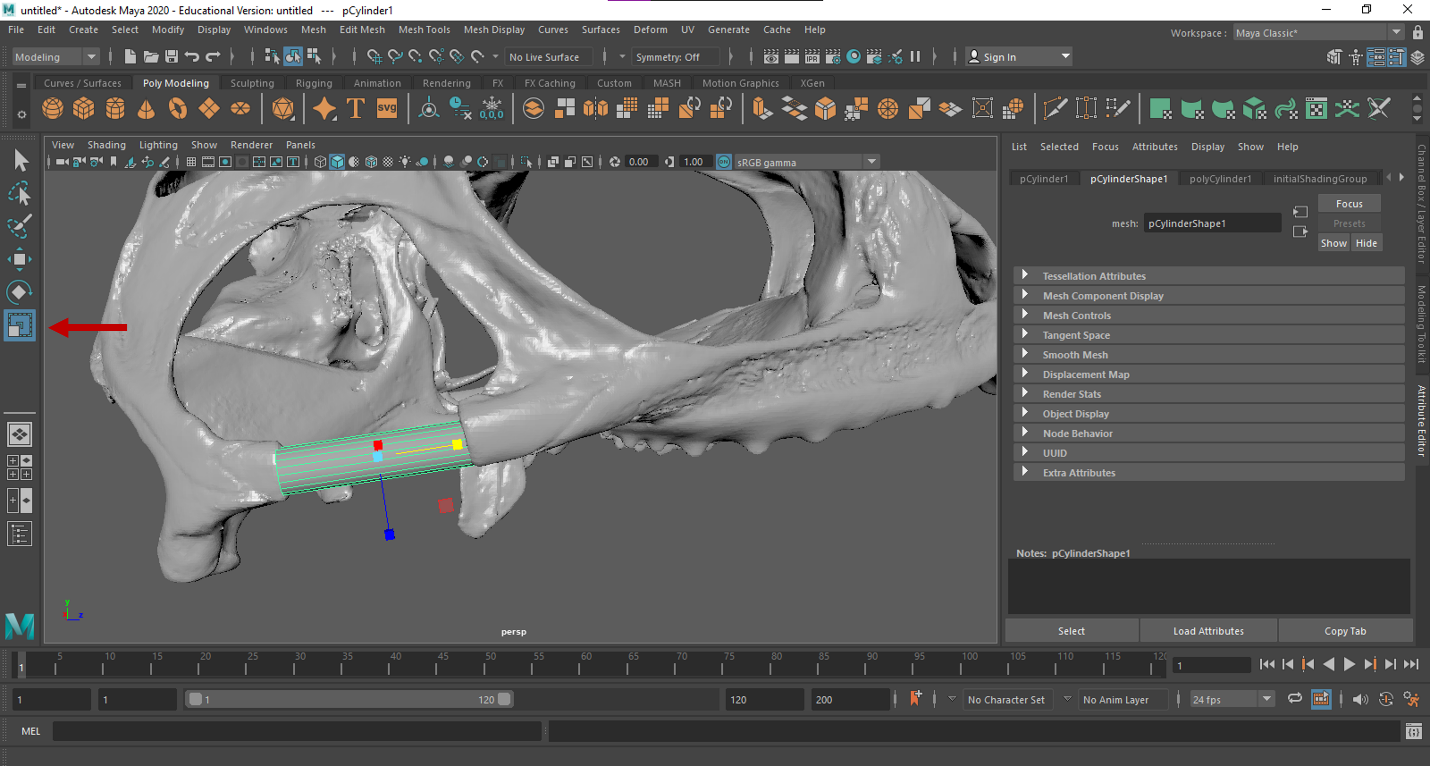


**Resize the bar in the dorsal and lateral directions using the scale tool** so to match the size and shape of the adjacent mesh. You may find it useful to toggle between the scale and rotate tools as you fit the new mesh to the skull mesh.


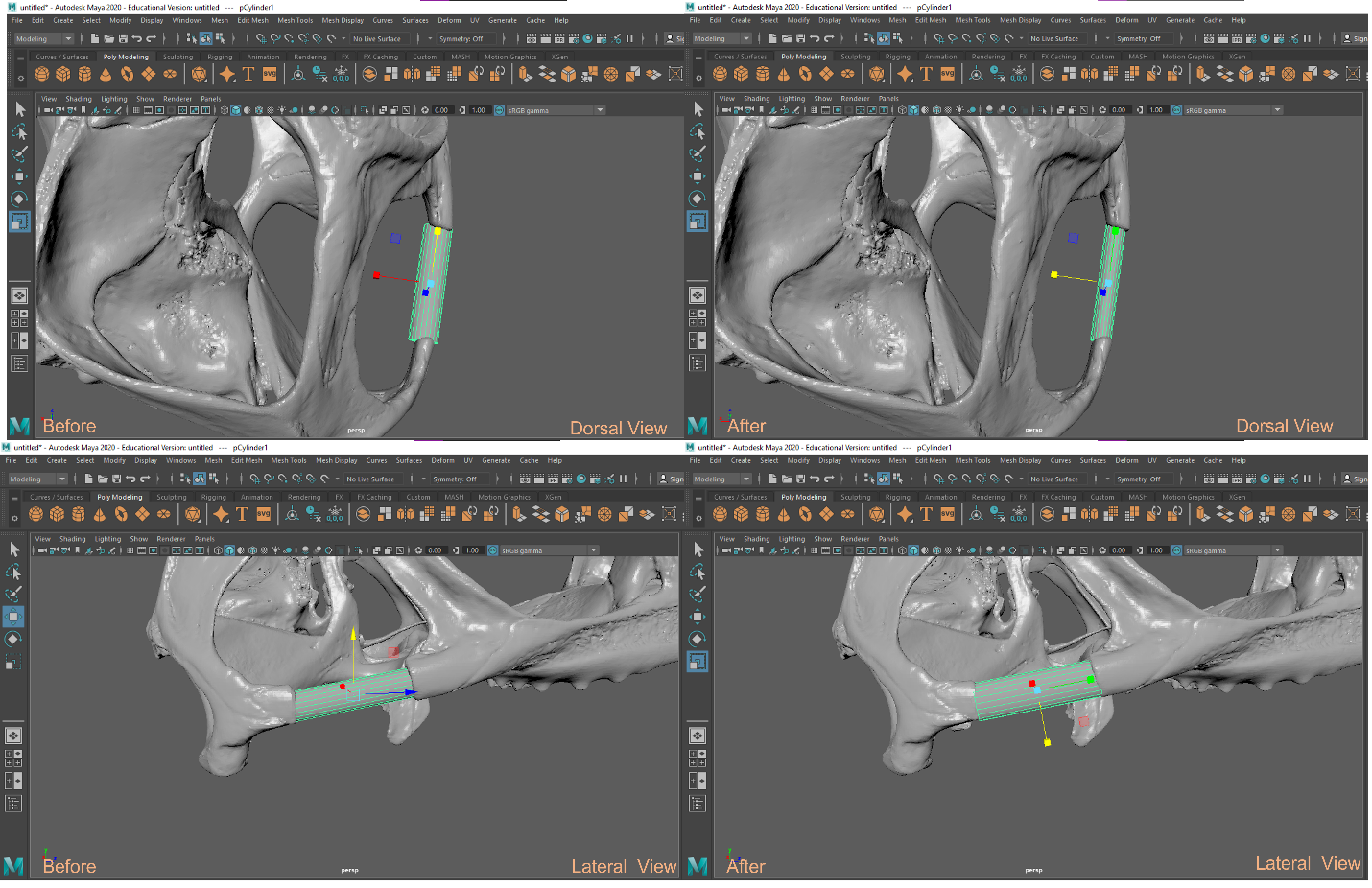


Once the cylinder is roughly sized, **add subdivisions by selecting the polyCylinder tab of the attribute editor.** You may notice that the “subdivisions axis” is already set to 20 subdivisions. **Add 20 subdivisions to the height** for fine tuning the mesh.


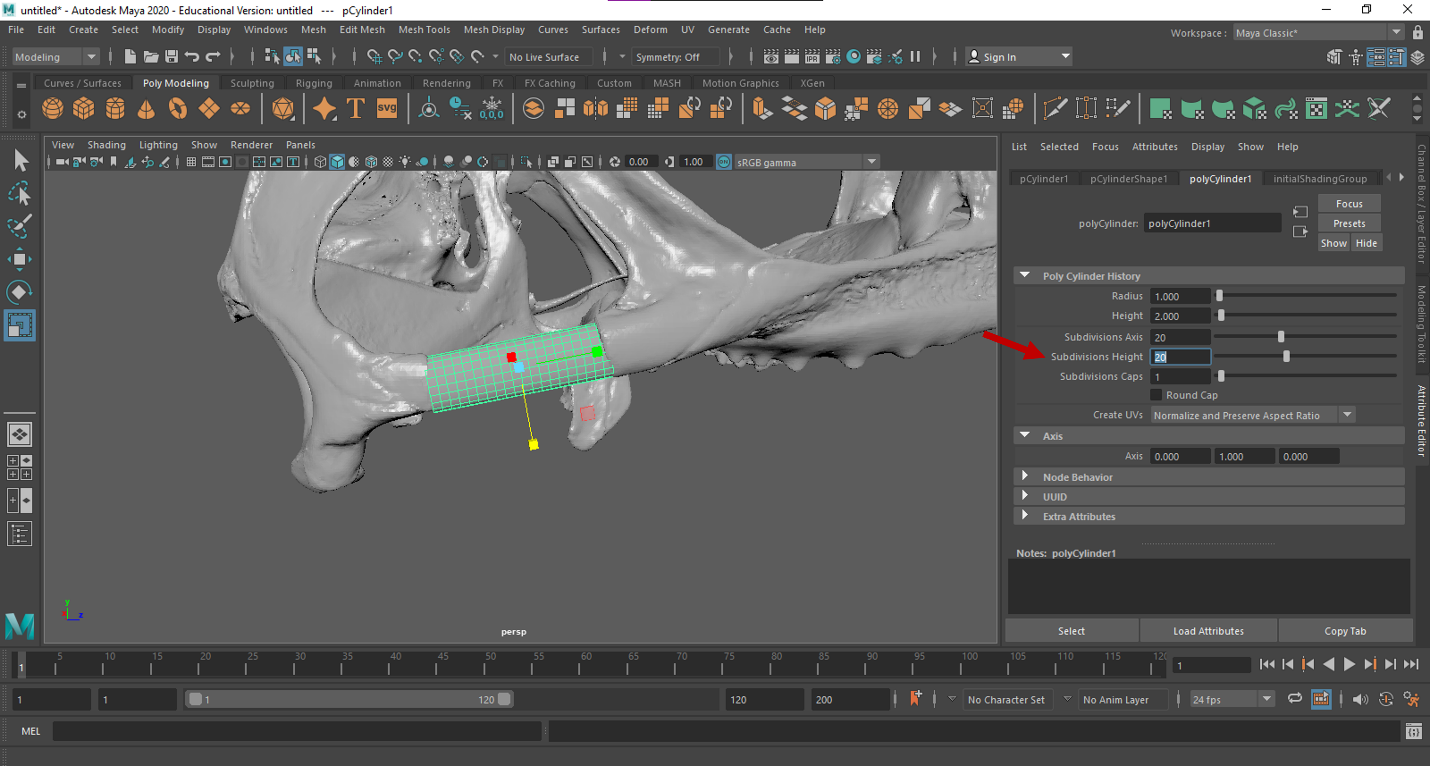


Similar to the simple patch method, **switch to edge mode by holding the right mouse button and selecting Edge.** Just like the patch, the **cylinder edges should be buried within the skull mesh to hide rough edges**.


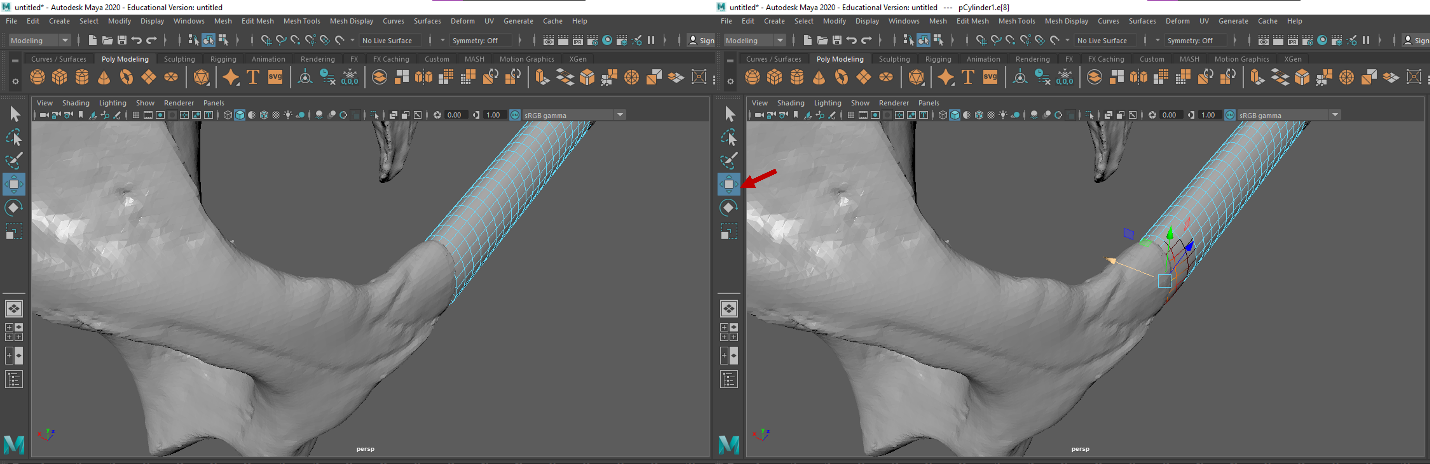


Fine tune the mesh by manipulating it until the desired shape is achieved, in this case it should look similarly to the post orbital bar on the opposite side of the model.


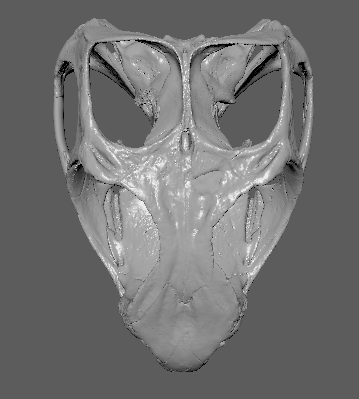

Dorsal view of patched skull

To combine the new meshes with the original model, enter object mode by **holding the right mouse button and selecting the “Object Mode” icon. Then select the selection tool icon.
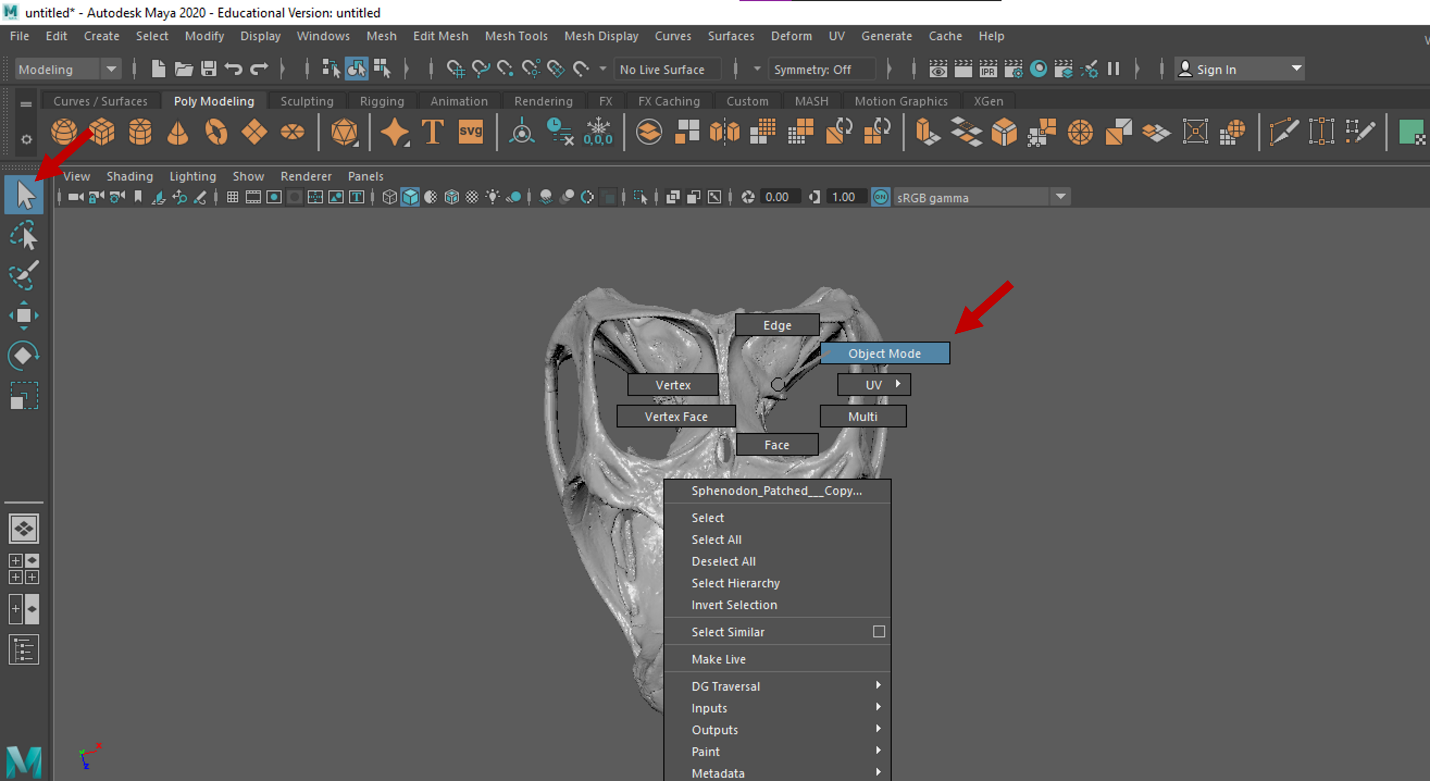
**

**Select all components (the original model and two patches) by dragging the cursor around all the meshes**, creating a box around all the elements in the scene.
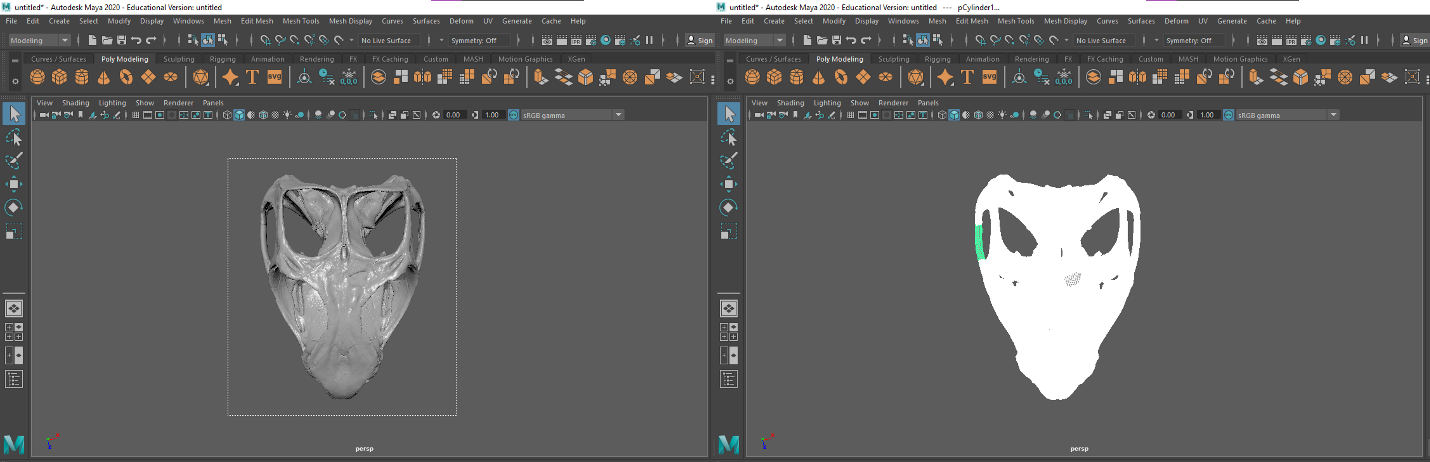


Combine the selection by going to **Mesh > Combine**.


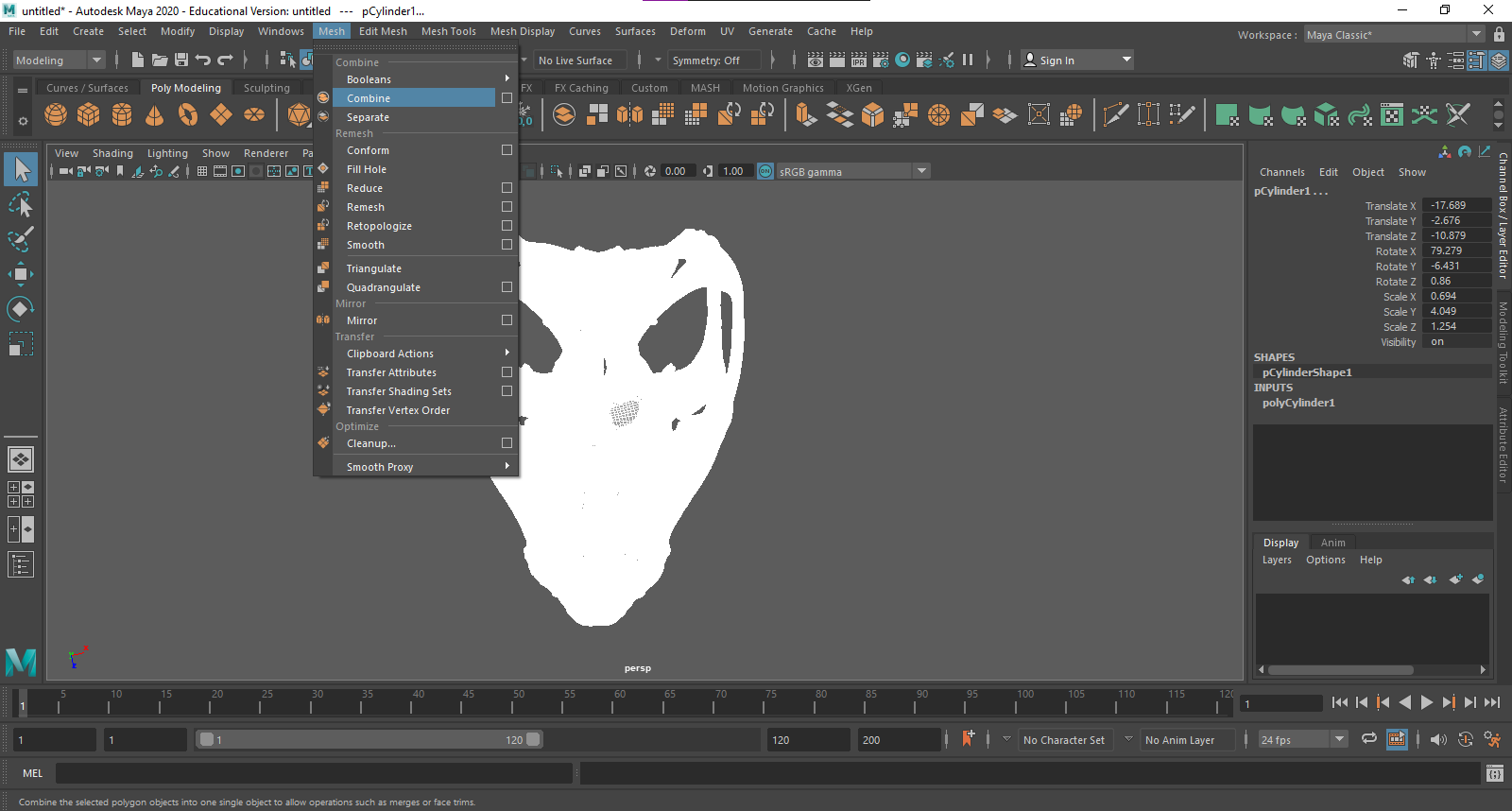


Export mesh by going to **File > Export Selection.** In the new pop-up window, name the file “Sphenodon Patched” and export selection as an .stl or .obj.
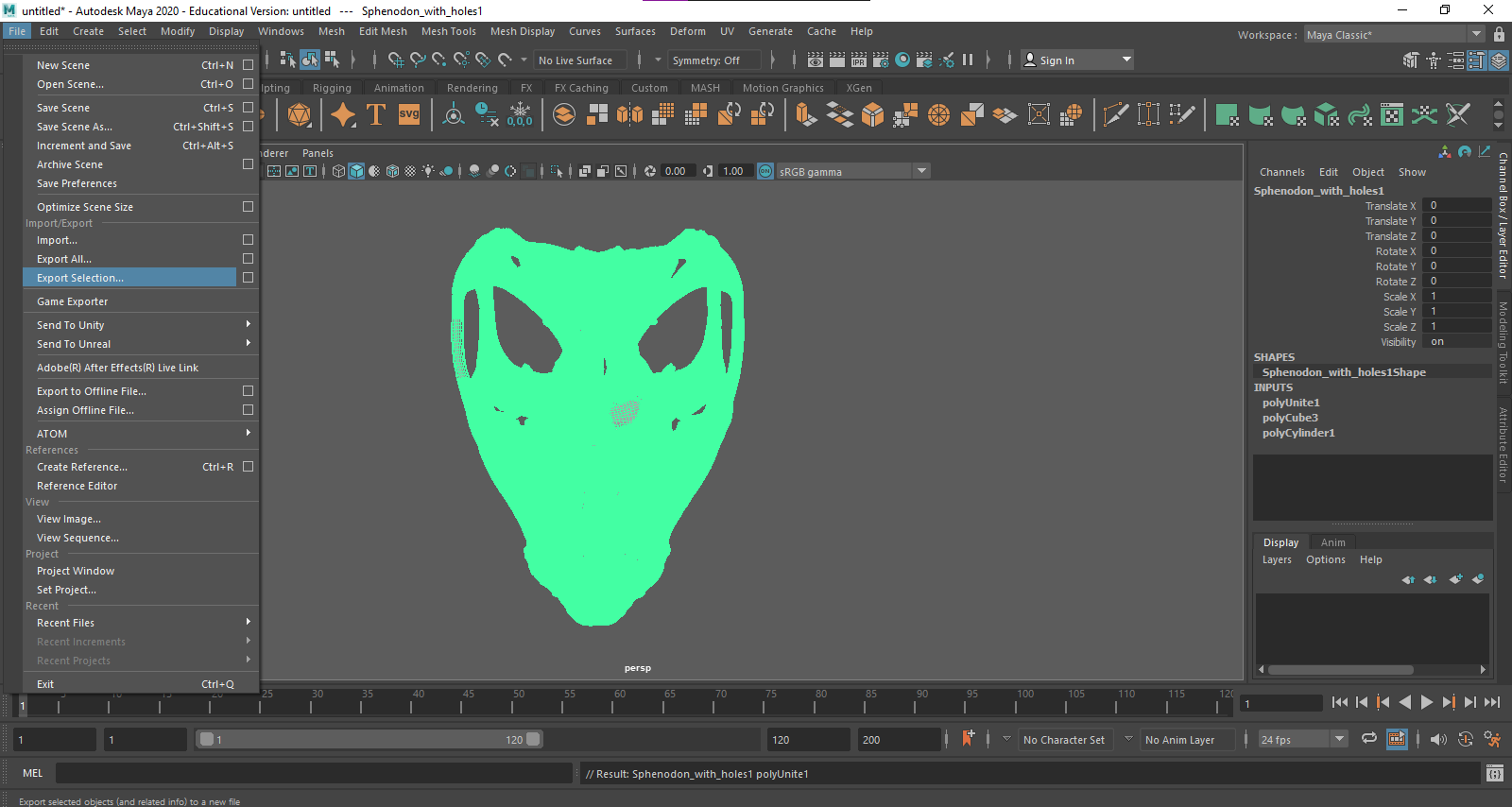


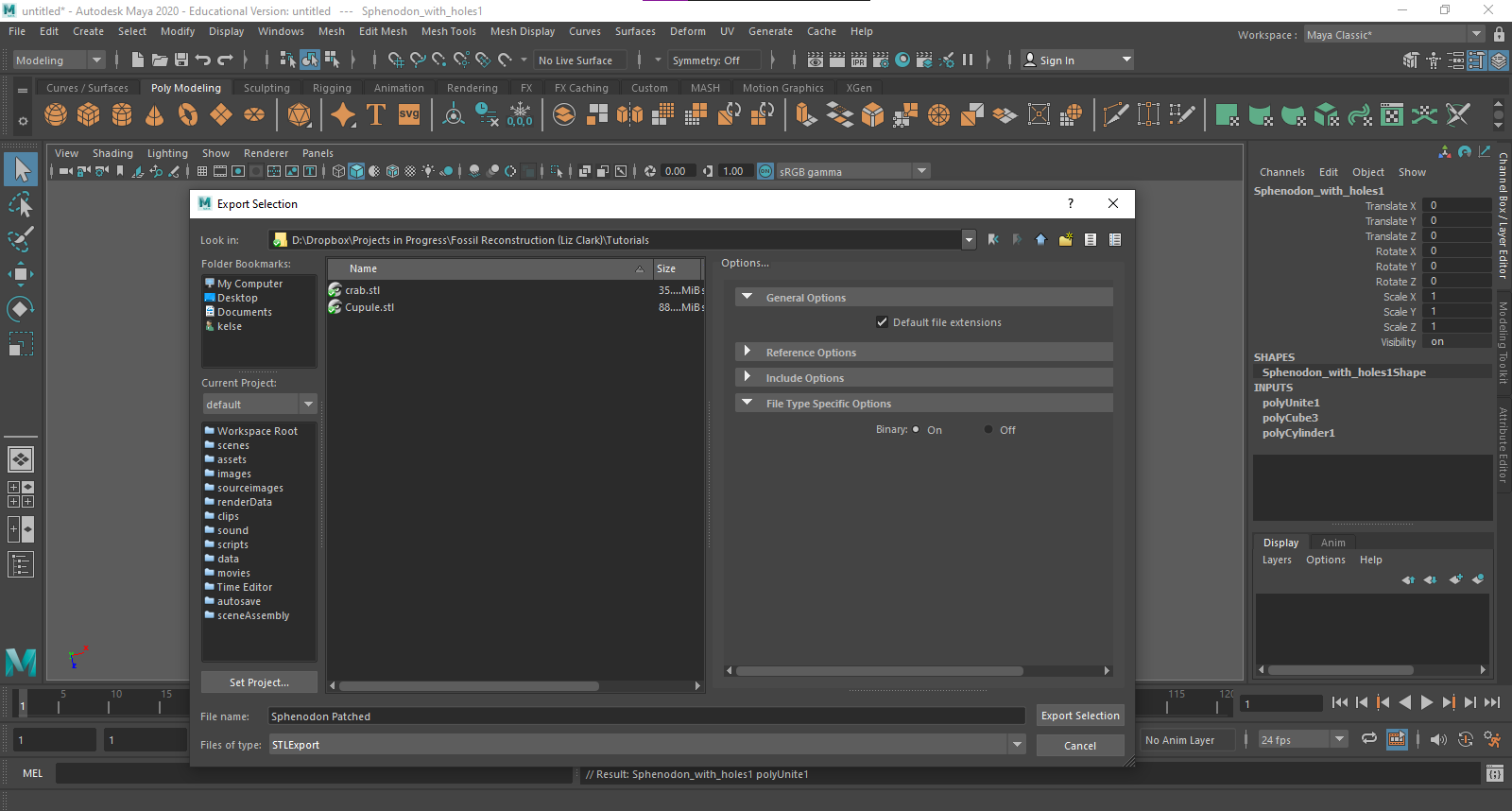


Shrinkwrapping and File Reduction

While the mesh is now patched, the model is still technically multiple meshes (the skull and two patches) instead of a single unified mesh. You may choose to keep the model in this format, though having multiple mesh elements may complicate downstream analyses. To rectify this, a shrinkwrap can be applied.

**Open Autodesk Fusion 360.** Open the file by **selecting the File Icon > Open… > Open from my computer…**

**
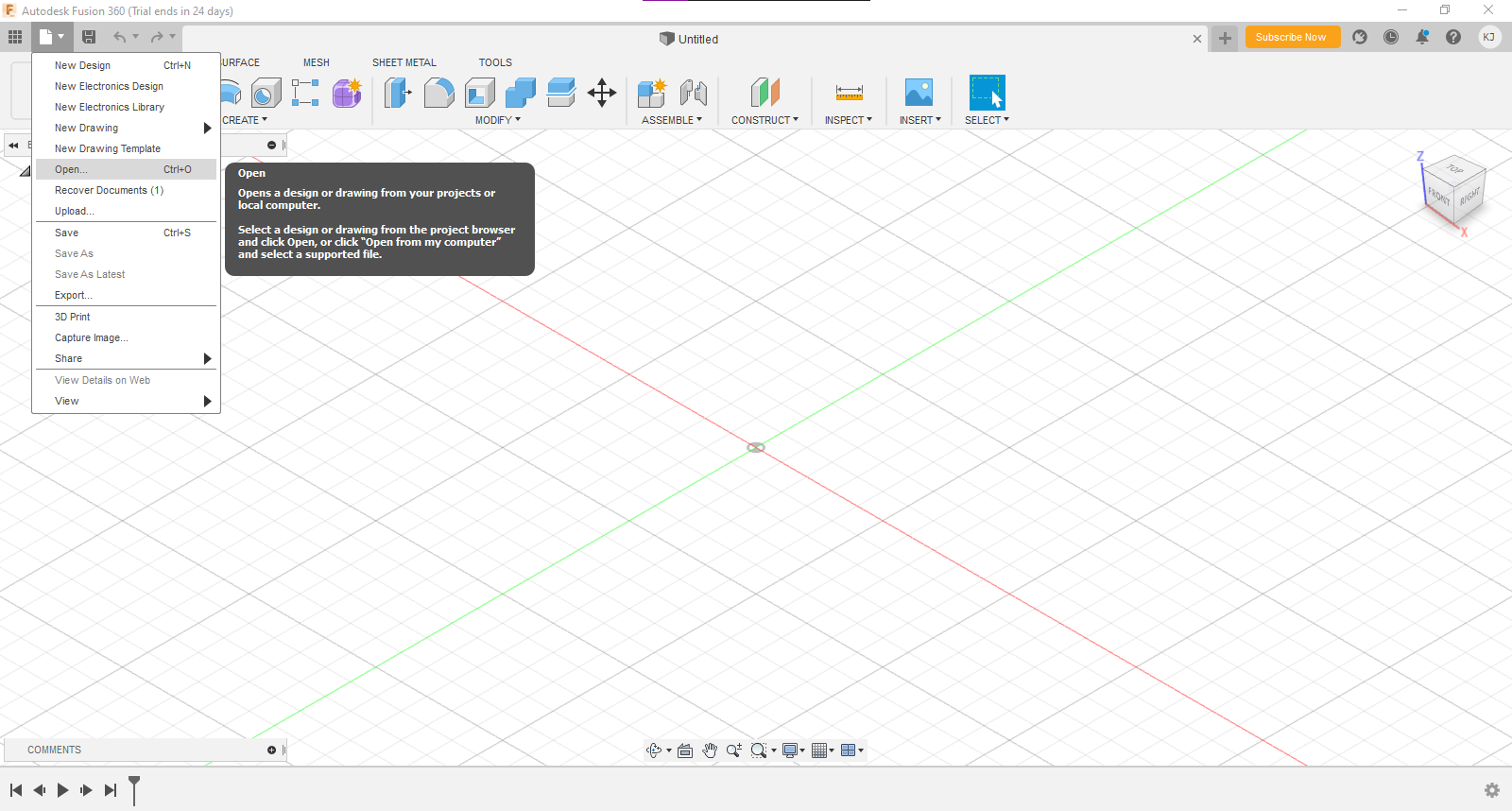
**

Once the model loads, the view can be rotated by toggling the view cube in the top right corner.

**
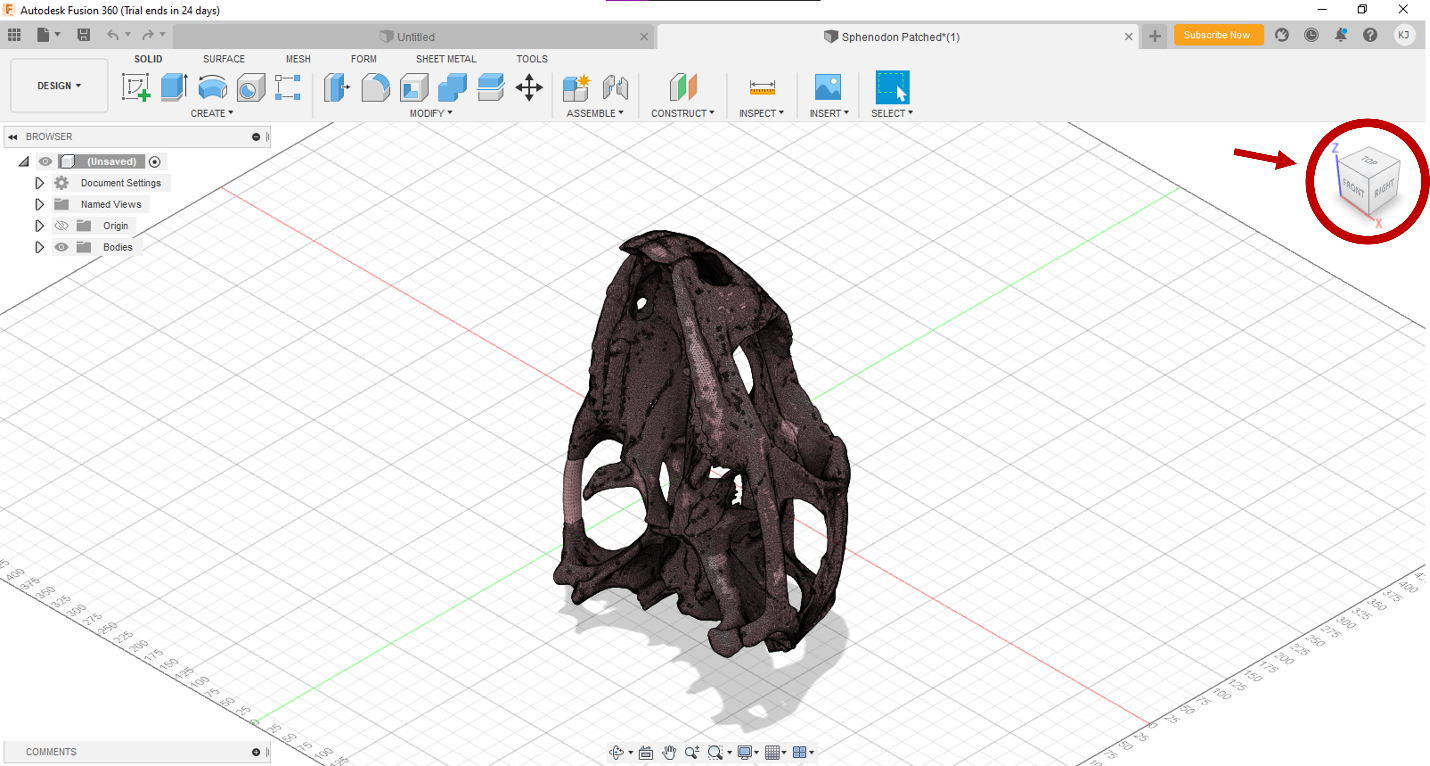
**

To begin, select the **Mesh tab.** Under that tab, select **Prepare > Repair**. A pop-up window will appear.


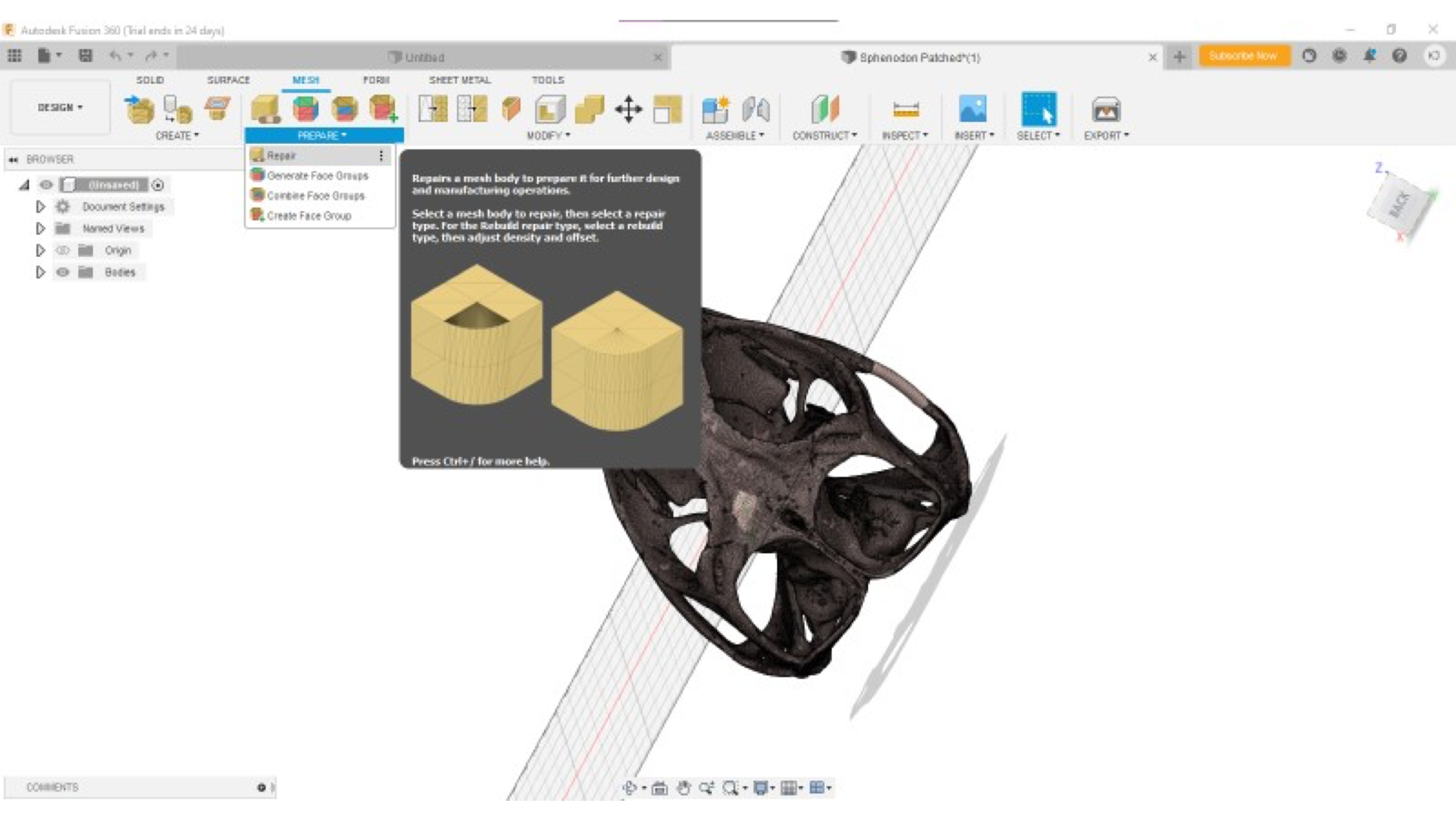
 **
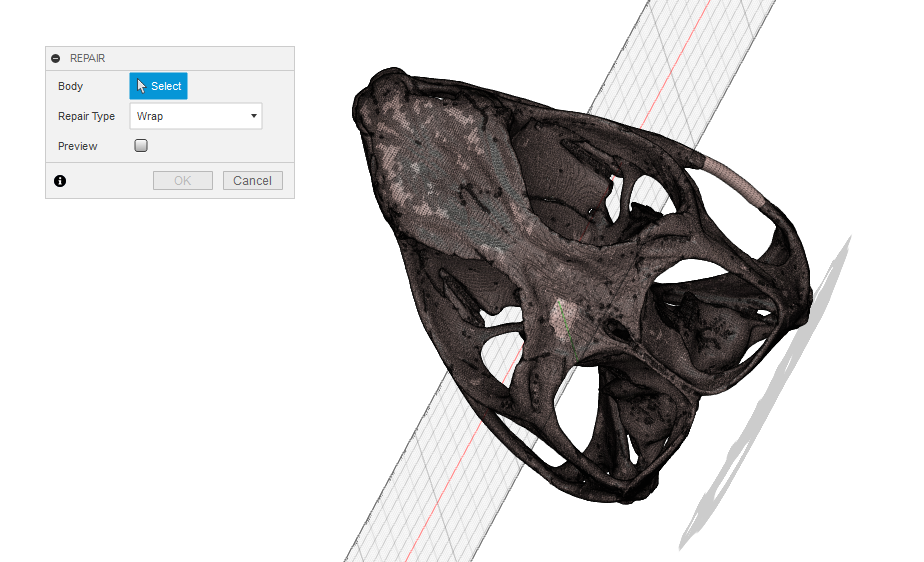
**

In the pop-up window **click Select and then select the model in the scene. Repair type should be changed to Wrap.** You may opt to view a Preivew by selecting the preview tab, but note that this task may take several minutes depending on the number of faces in the model. **Click OK to create the shrinkwrap.**

Once the shrink wrapping is complete, the mesh can be exported. However, you may want to consider also minimizing the number of faces in the mesh to further simplify the model to reduce the total file size. Be aware that this will also reduce some of finer details in the model. To do this, select **Mesh > Modify > Reduce**. A pop-up dialog box will appear.


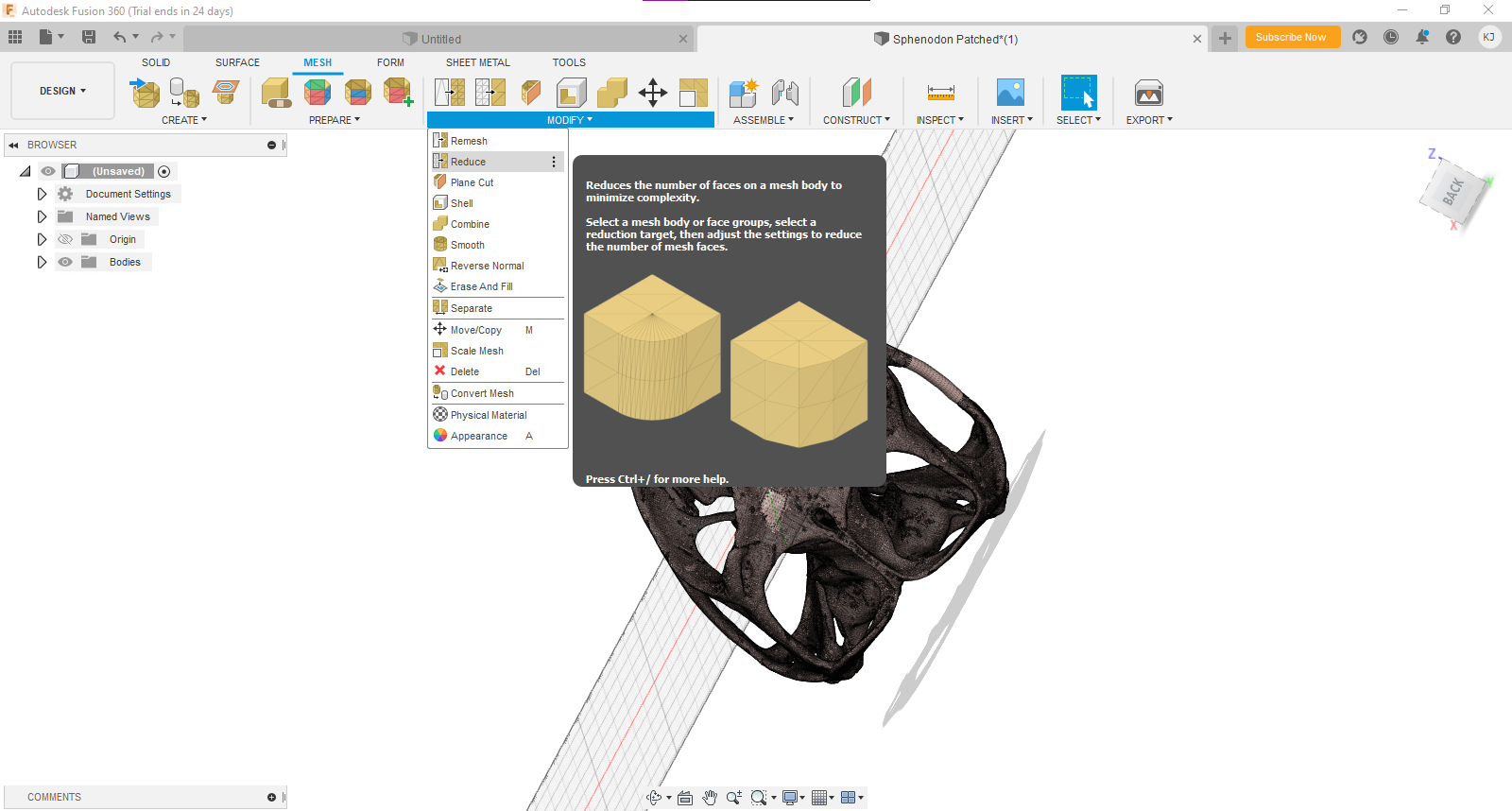


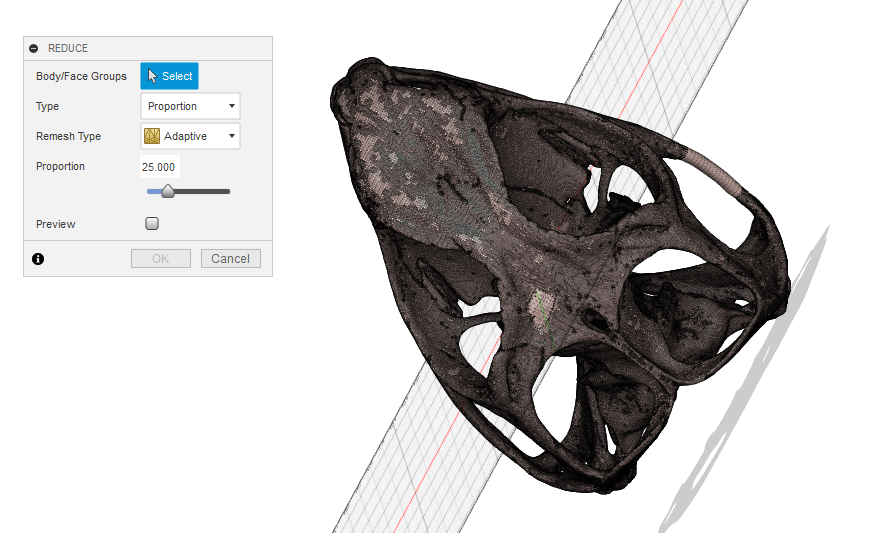


**Click select and select the model in the scene by dragging a box around the model.** There are several ways to reduce the face count, either by proportion or by face number. In this example, the number of faces are reduced by a proportion of 25, in that the original number of faces are reduced to 25% of the original value. Remesh type is Adaptive. Again, you may opt to view a preview. **Select OK.**


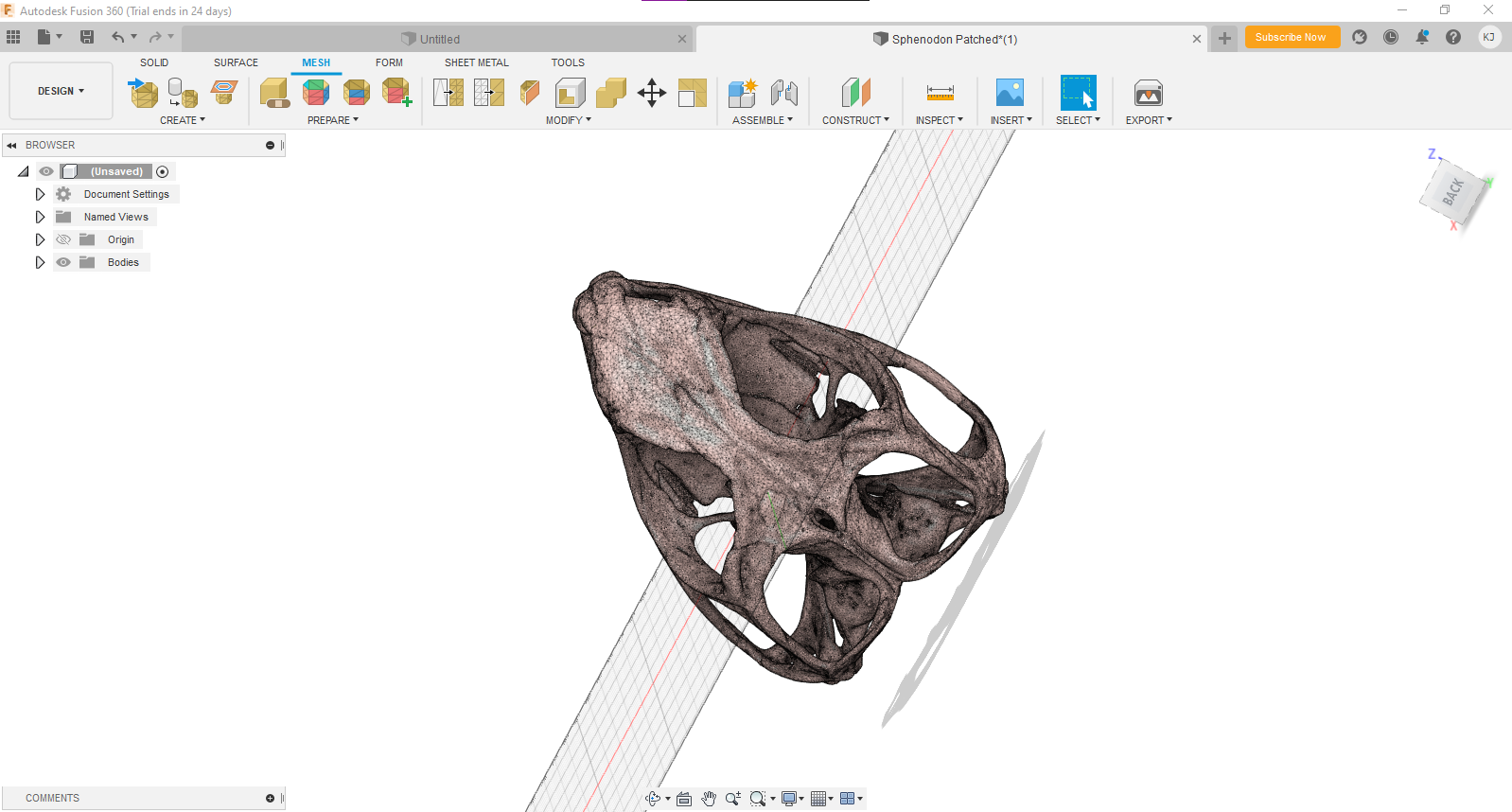


To export the model, **select the File Icon > Export.**


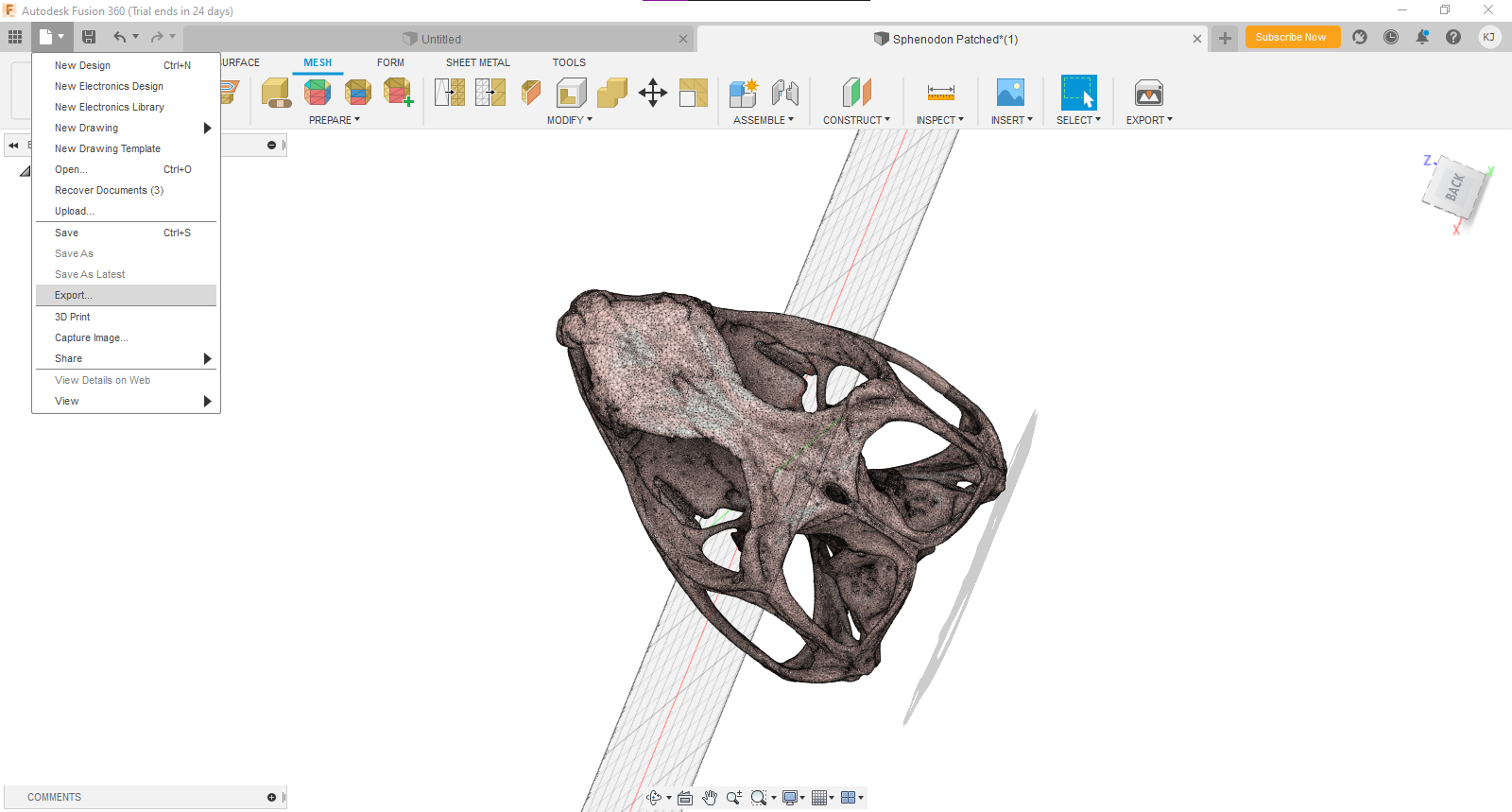


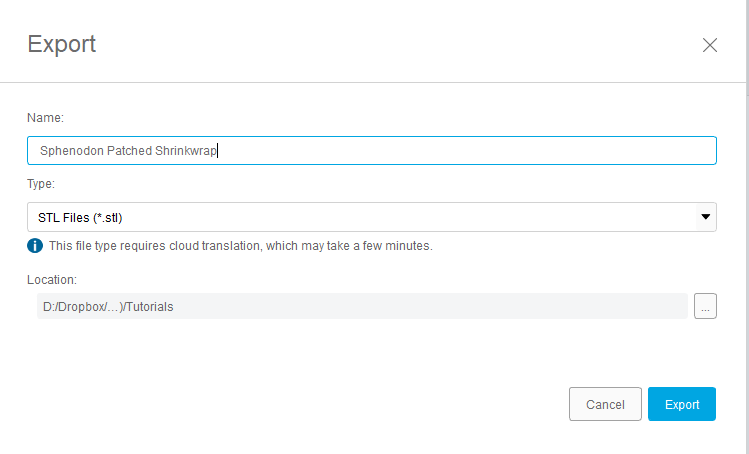


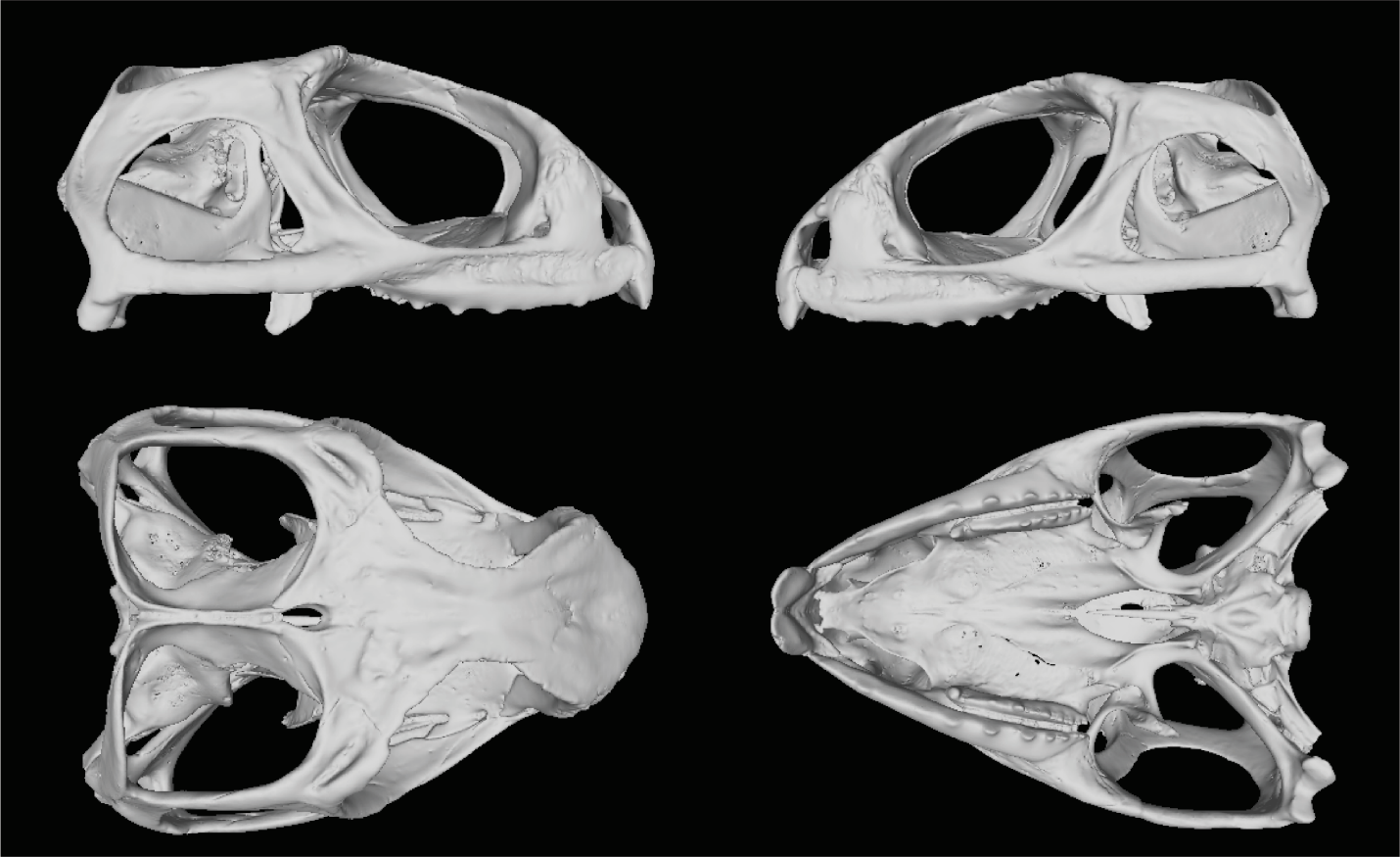


Repaired model

**3D Mesh Cleanup Tutorial: Fossil Crab (Advanced)**

*Skills developed*: Advanced mesh editing

3D meshes of fossil specimens extracted directly from 3D imaging data often need post-processing to be useful for paleontological analyses. Here, we present the workflow that was used to digitally process *Cretapsara athanata* (Luque et al. 2021), the most complete fossil crab ever discovered, as a tutorial. This tutorial uses two pieces of software: the opensource mesh editing program Meshlab (Meshlab.net) and the 3D modeling software Autodesk Maya (autodesk.com/products/maya) that can be downloaded for free by students or through an institutional subscription. It is highly recommended that the first two tutorials in this series are completed before beginning this one. Note that this tutorial utilizes Meshlab 2021.10 and Autodesk Maya 2017, and other versions may have slightly different layouts and functions.


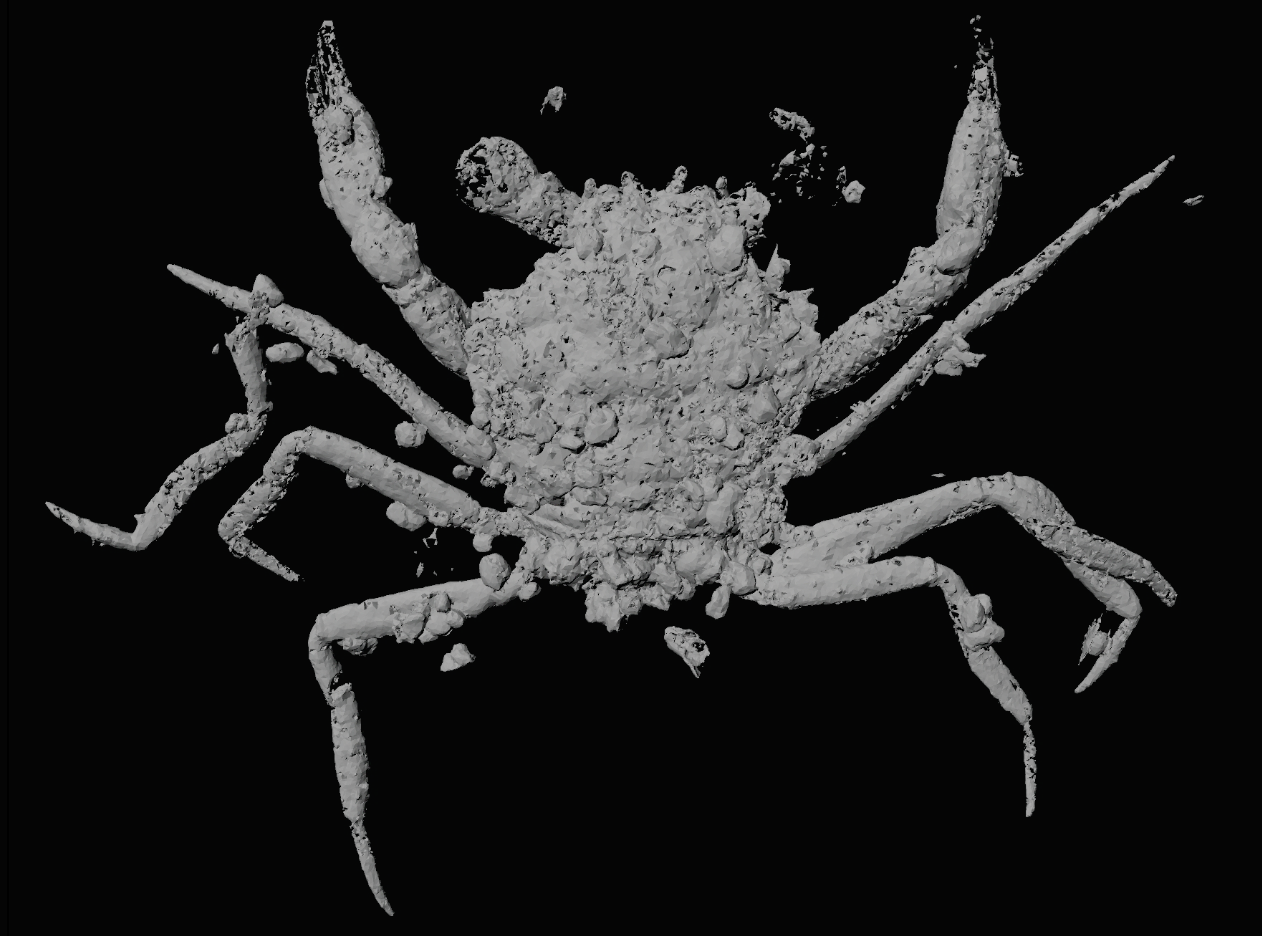


When the digitized fossil crab was first exported as a mesh from the 3D processing software, a number of common issues were evident. The right eye is missing, a leg is detatched, tips of the claws are floating, isolated faces, and debris surround and abut the specimen. This tutorial will walk through how to rectify these issues.

Import the crab into Meshlab by following Part 1 in the Cupule Cleanup Tutorial.


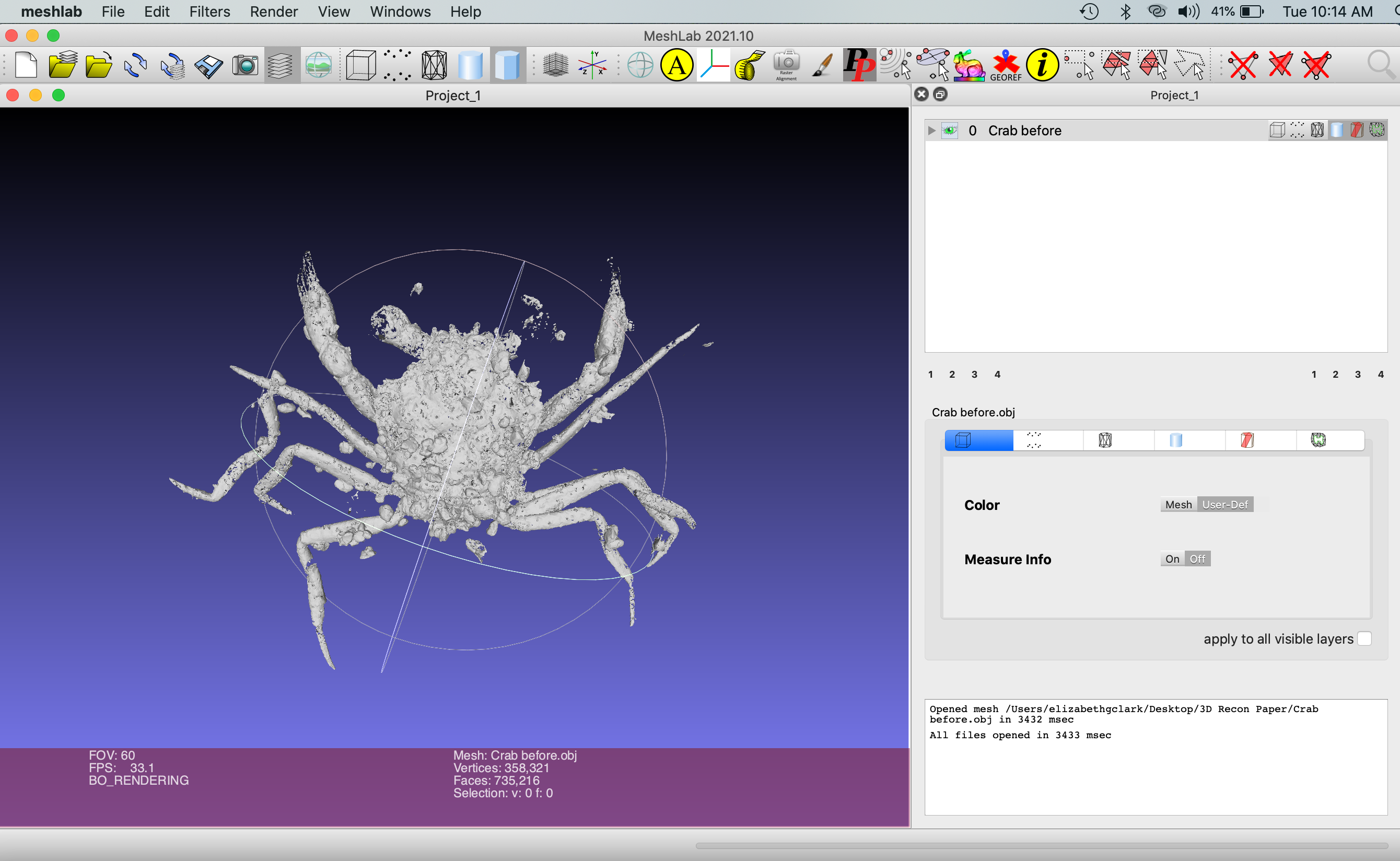


*Remove External Debris*

The imported mesh includes the crab, but it also includes other small isolated meshes that are 3D reconstructions of bits of the matrix. Remove this external debris by running a filter that removes small isolated mesh particles. **Go to Filters** > **Remeshing, Simplification and Reconstruction** > **Remove isolated pieces with respect to face number**. This option may be under “Cleaning and Repairing” in other versions of the software. As the 3D mesh of the crab is a large connected mesh with a relatively high number of faces, start by removing meshes with a relatively small number of faces (e.g., 200) and increase incrementally (e.g., by 50-500) until the bulk of the isolated extraneous mesh pieces are removed.


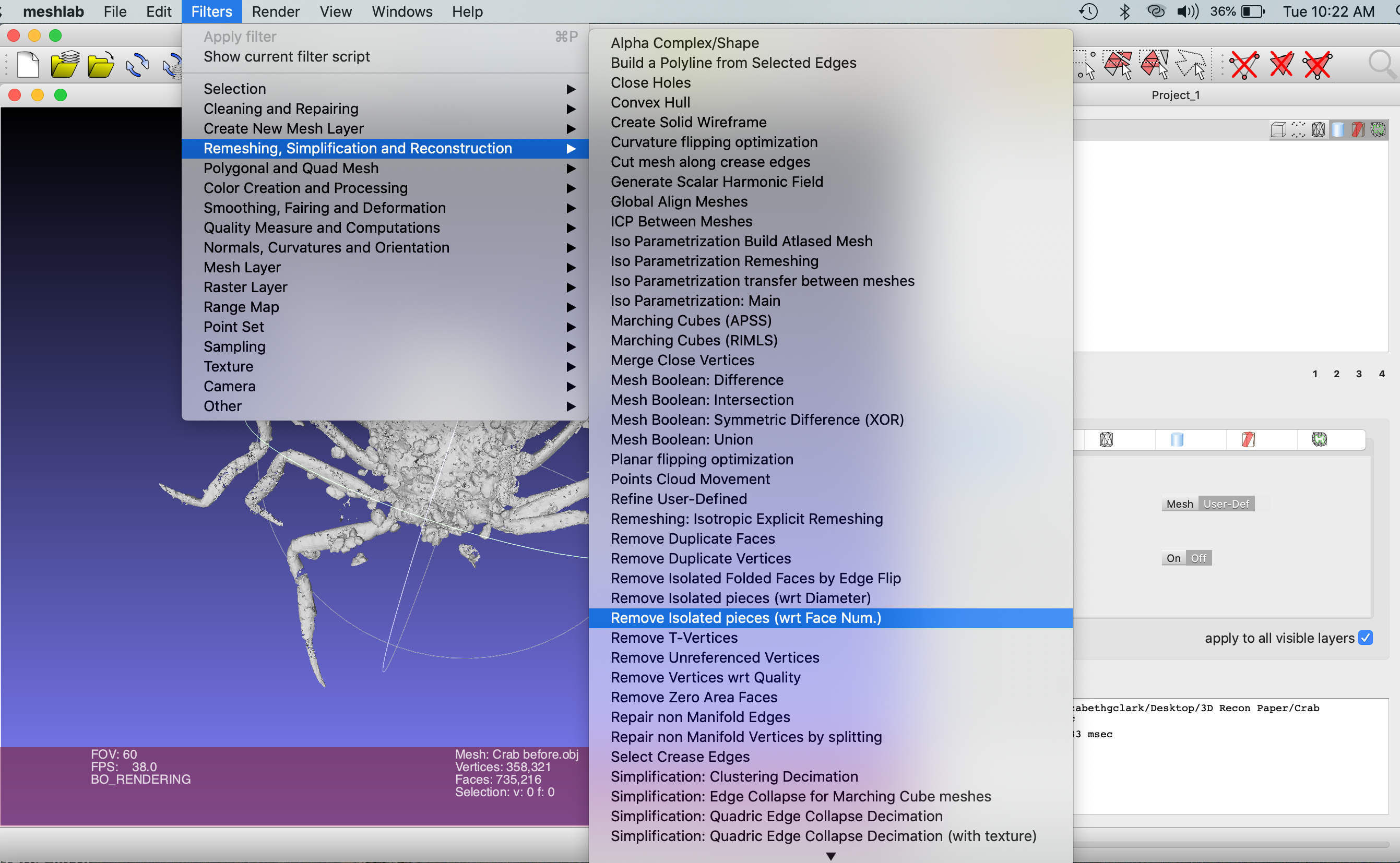


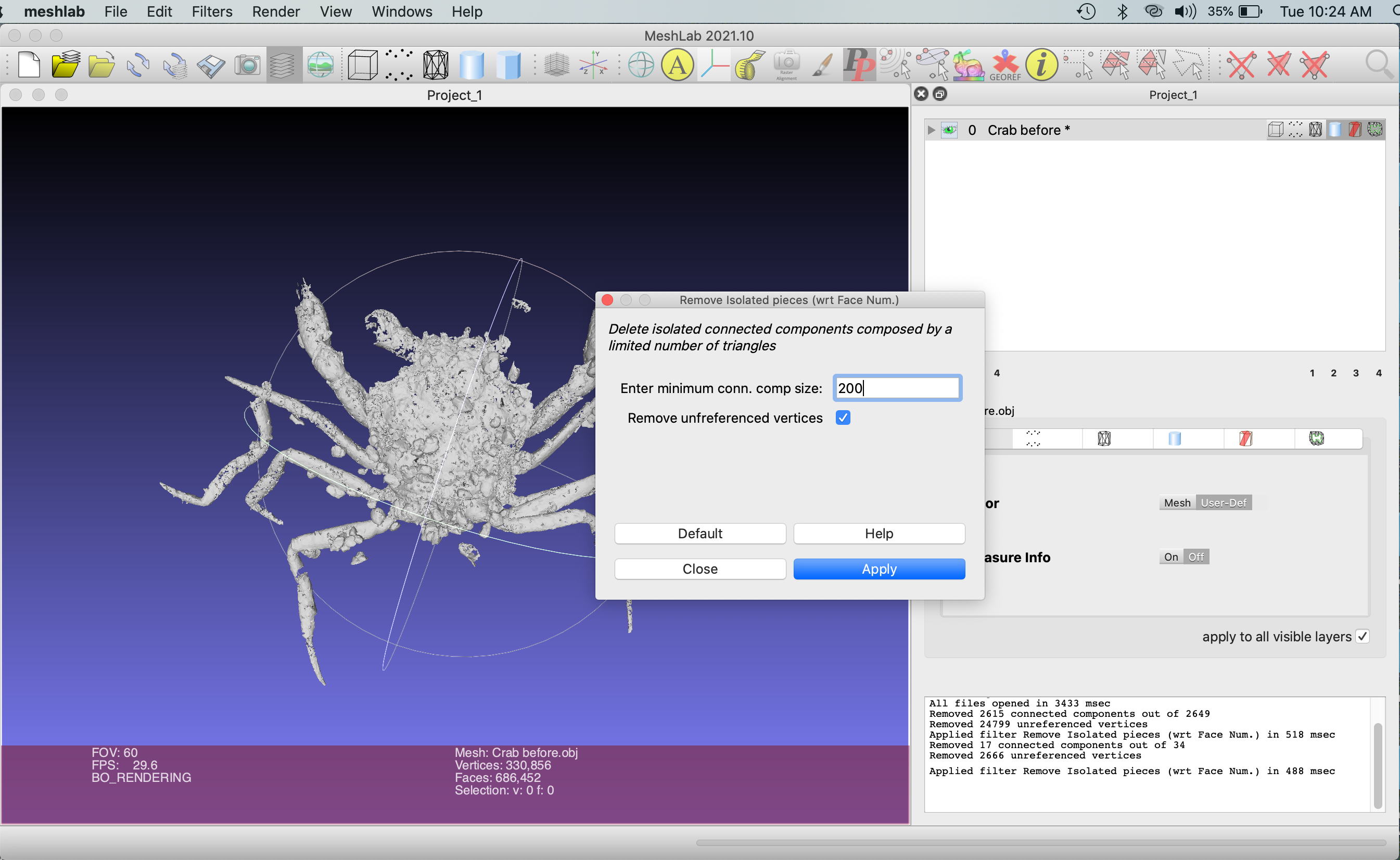


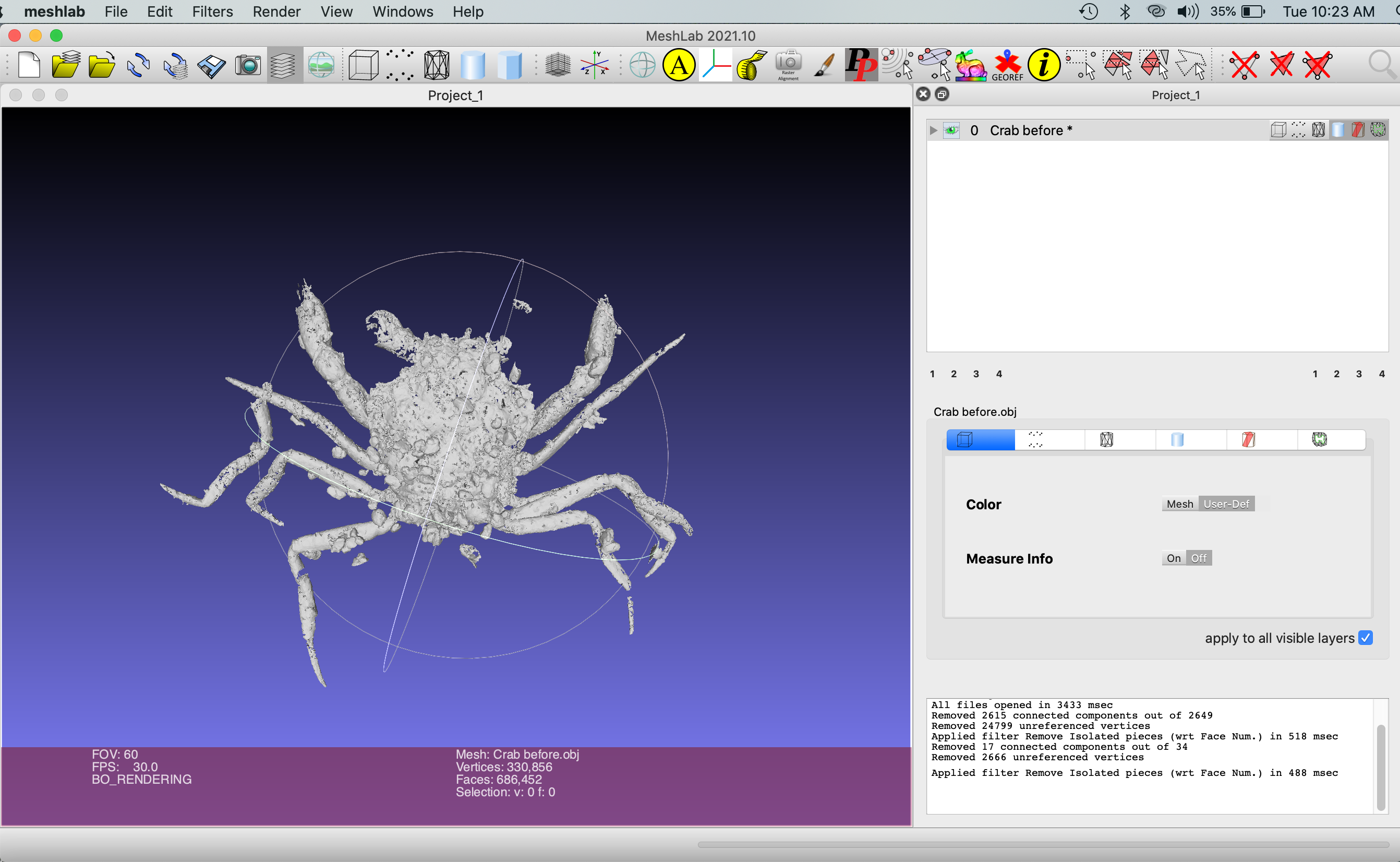


The scene still contains a few relatively large bits of extraneous isolated meshes. To select and delete these faces manually, first **click on the Interactive Selection tool** in the top right of the icon-based menu.


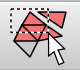


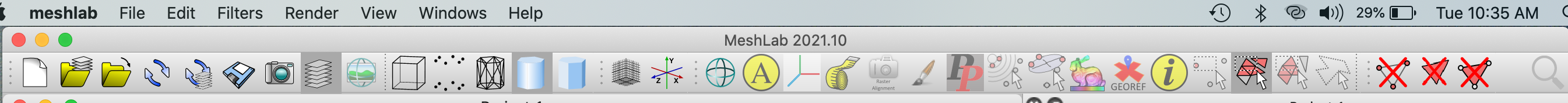


Hold left click and drag your cursor to select faces. Note that, while this tool is on, the faces you selected previously will become deselected once you click somewhere else in the project window. To add faces to your selection, **hold command** (Mac) or **ctrl** (PC) **while you left click and drag**. To delete faces from your selection, **hold shift while you left click and drag**.


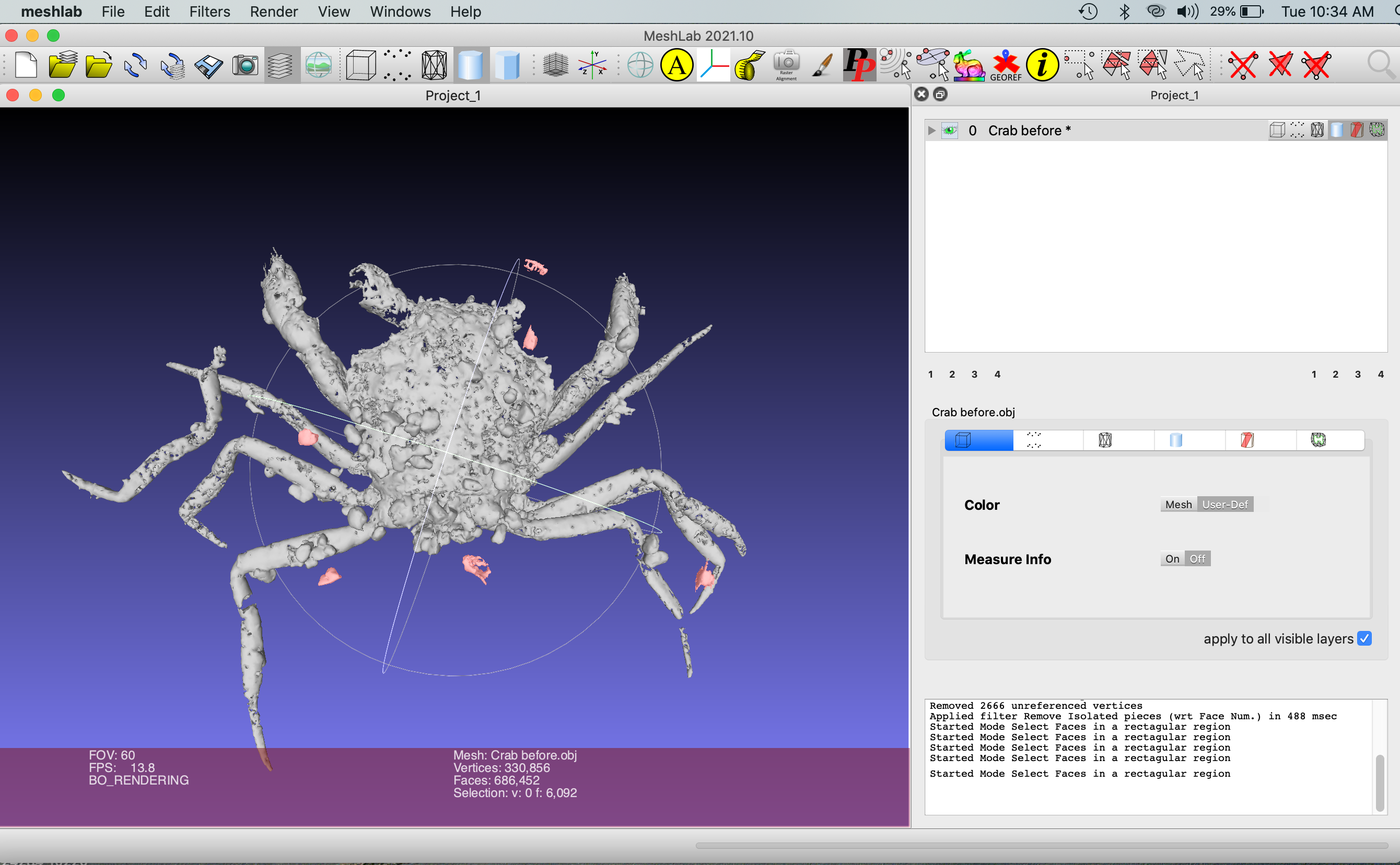


Delete these faces by **deselecting the Interactive Selection tool** and **selecting** **“Delete Selected Faces and Vertices”** on the right of the icon-based menu.


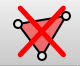


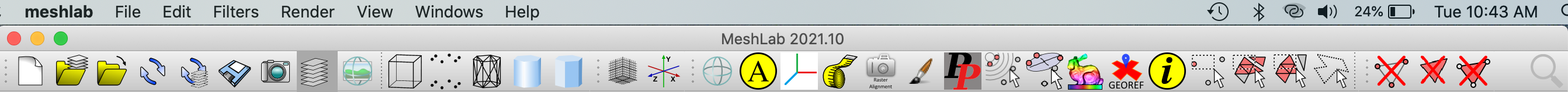


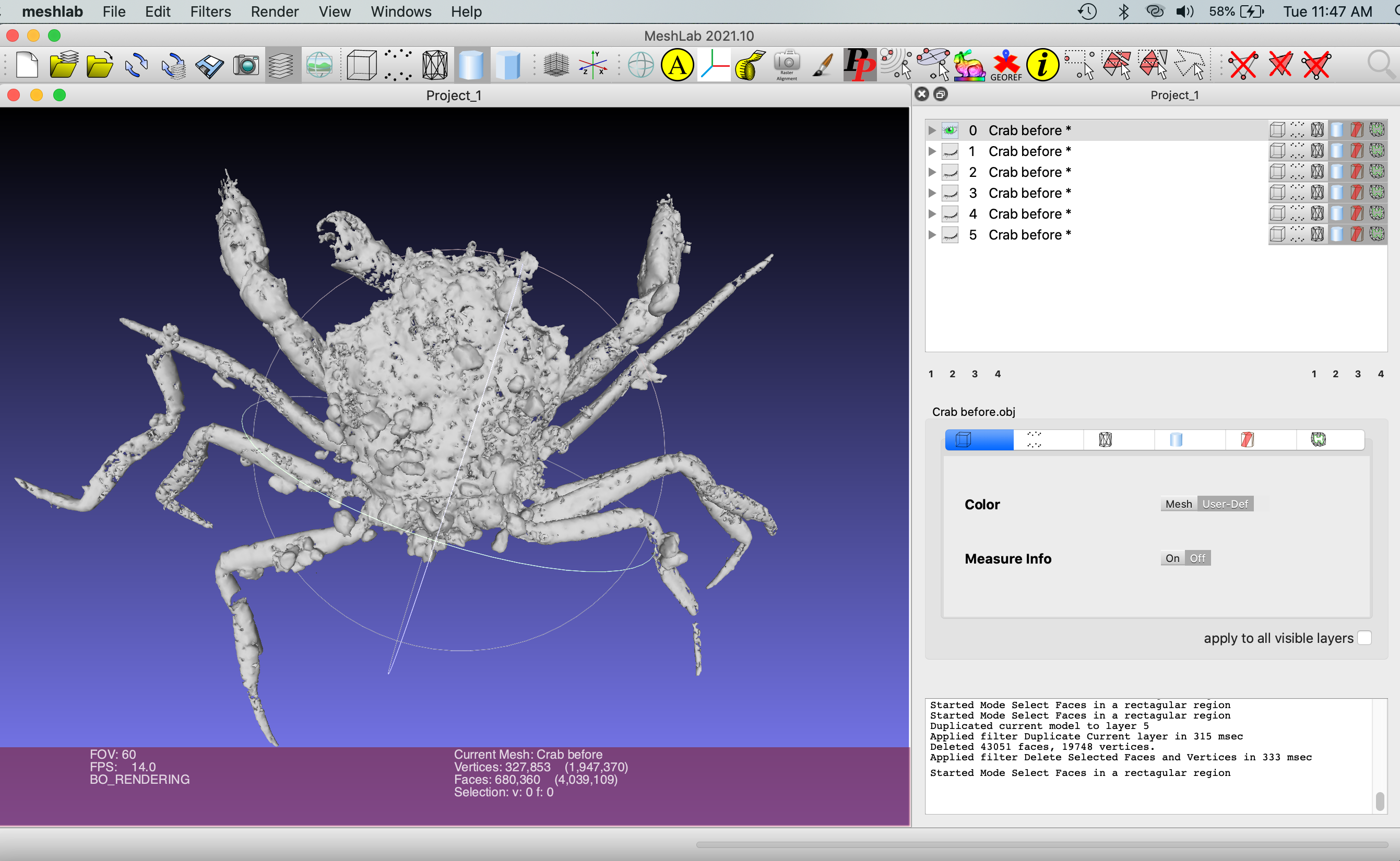


Next, repeat these steps to select and delete the small, rounded pebbles that are touching the crab mesh. Note that your selection goes through the entire mesh in that plane of view. Be sure to minimize the number of faces of the crab mesh itself that are deleted; this can be done by turning off the Interactive Selection tool (
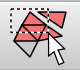
) and rotating the mesh to ensure no pieces of the crab mesh were accidentally selected before deleting mesh components. We recommend duplicating the mesh, as Meshlab does not have an undo function, in case desired components of the mesh are accidentally deleted or deselected. Duplicate the mesh by **right clicking the file name** and **selecting “Duplicate the Current Layer.”** Select the eye icon on the original layer to toggle visibility so you can see deletions on the new layer more clearly. You may wish to delete extraneous mesh little-by-little as you are just beginning, as opposed to all at once (as shown in the image below).


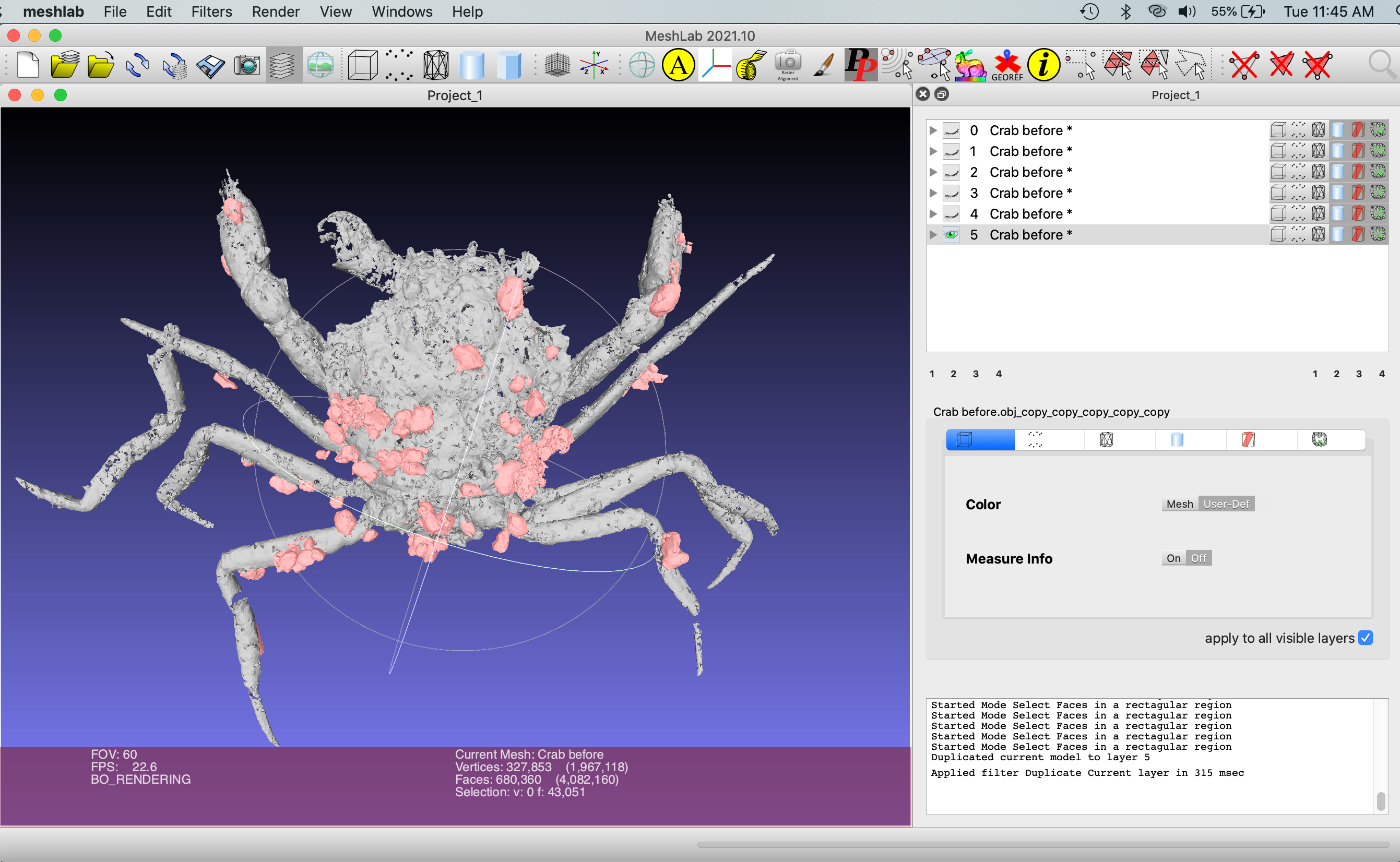


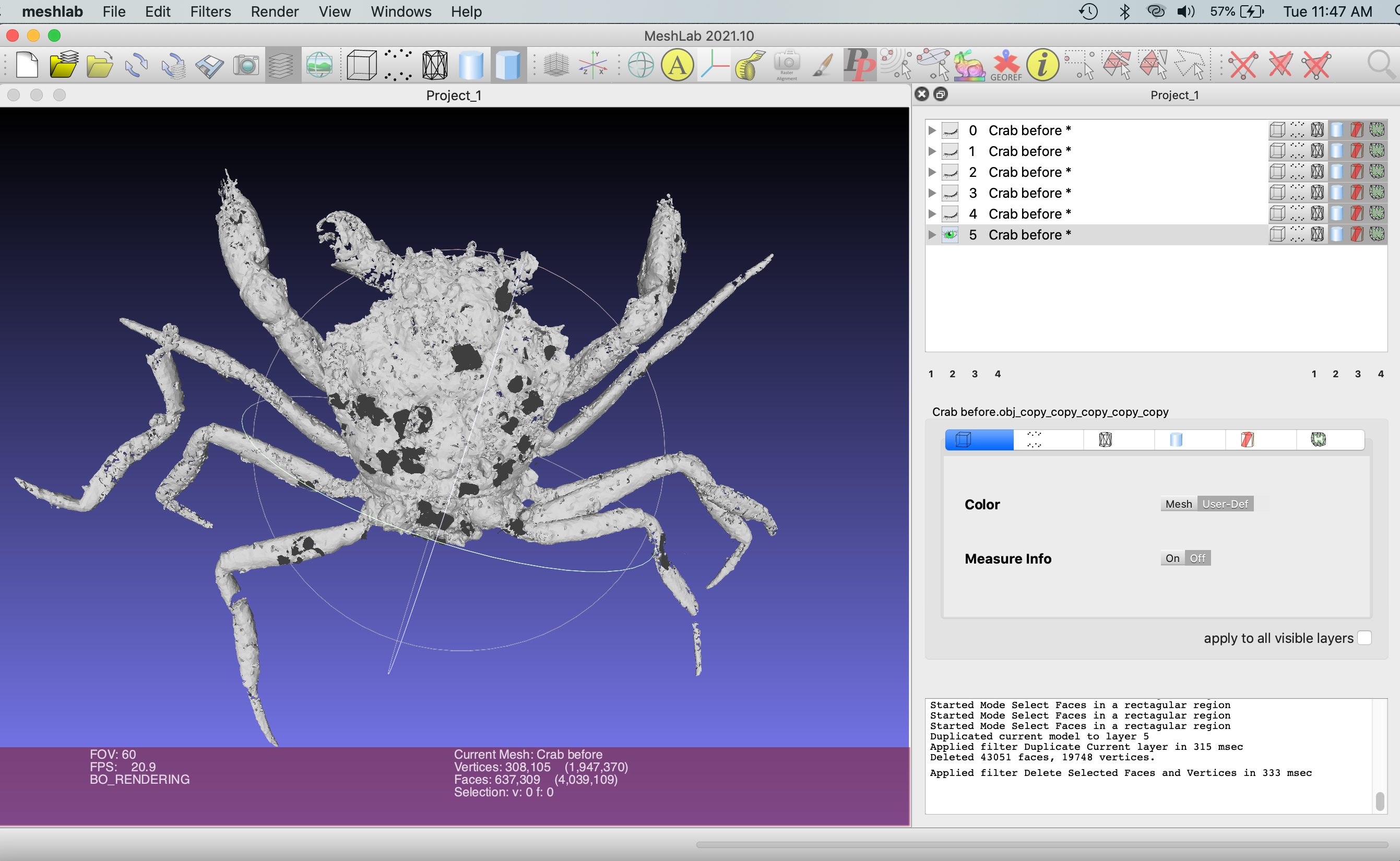


*Fill Holes*

Clean up the mesh by selecting **Filters** > **Remeshing, Simplification and Reconstruction** > **Repair non Manifold edges**. **Select Remove Faces** and **click Apply**. Then, remove small isolated pieces that might have been formed during previous steps repeating **Filters** > **Remeshing, Simplification and Reconstruction** > **Remove isolated pieces with respect to face number**. Save this mesh layer by duplicating it.

Close holes that formed in the crab mesh during the deletion of articulating debris by selecting **Filters** > **Remeshing, Simplification and Reconstruction** > **Close Holes**. Set **“Max size to be closed” to 500** and **select Apply**.


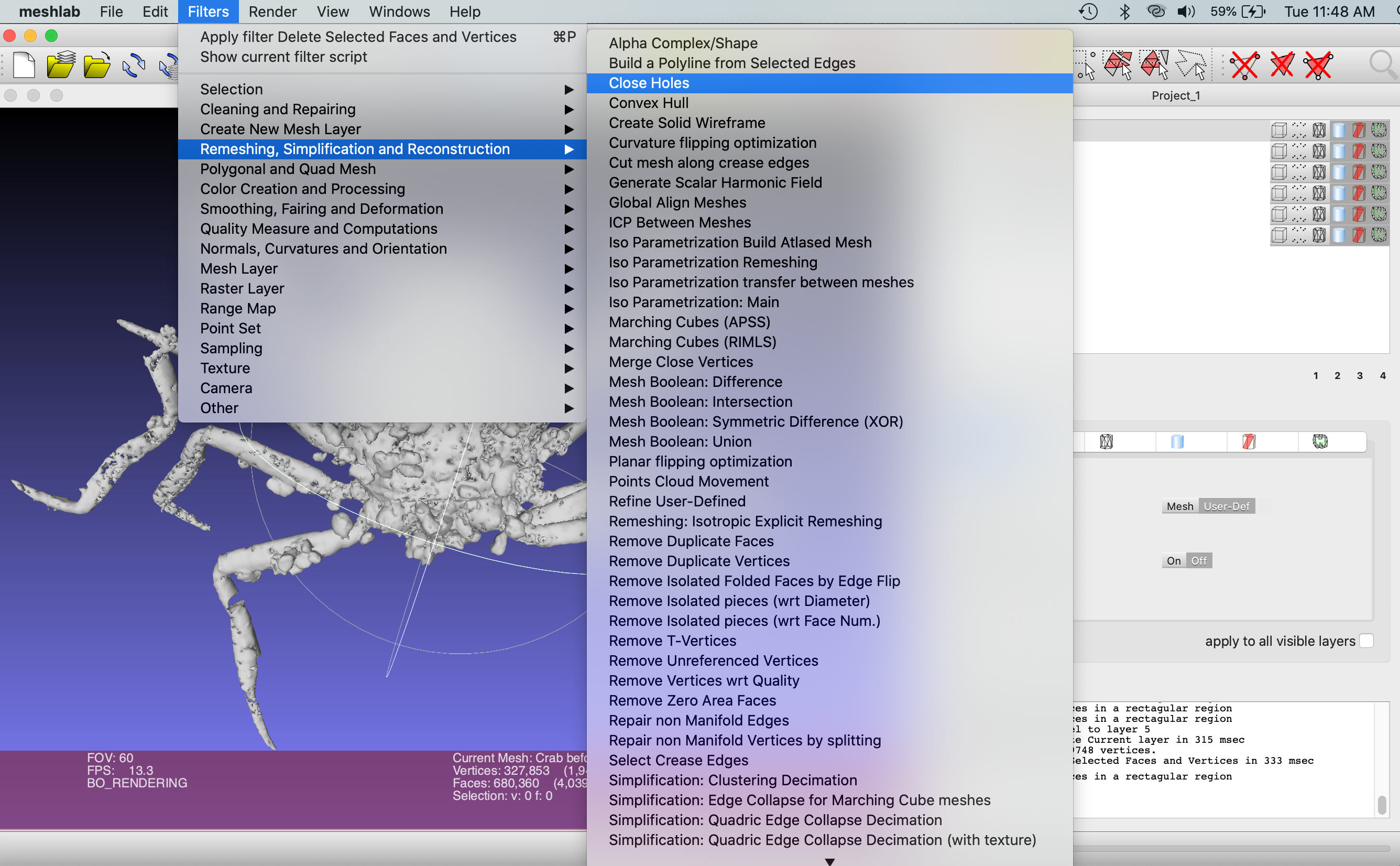


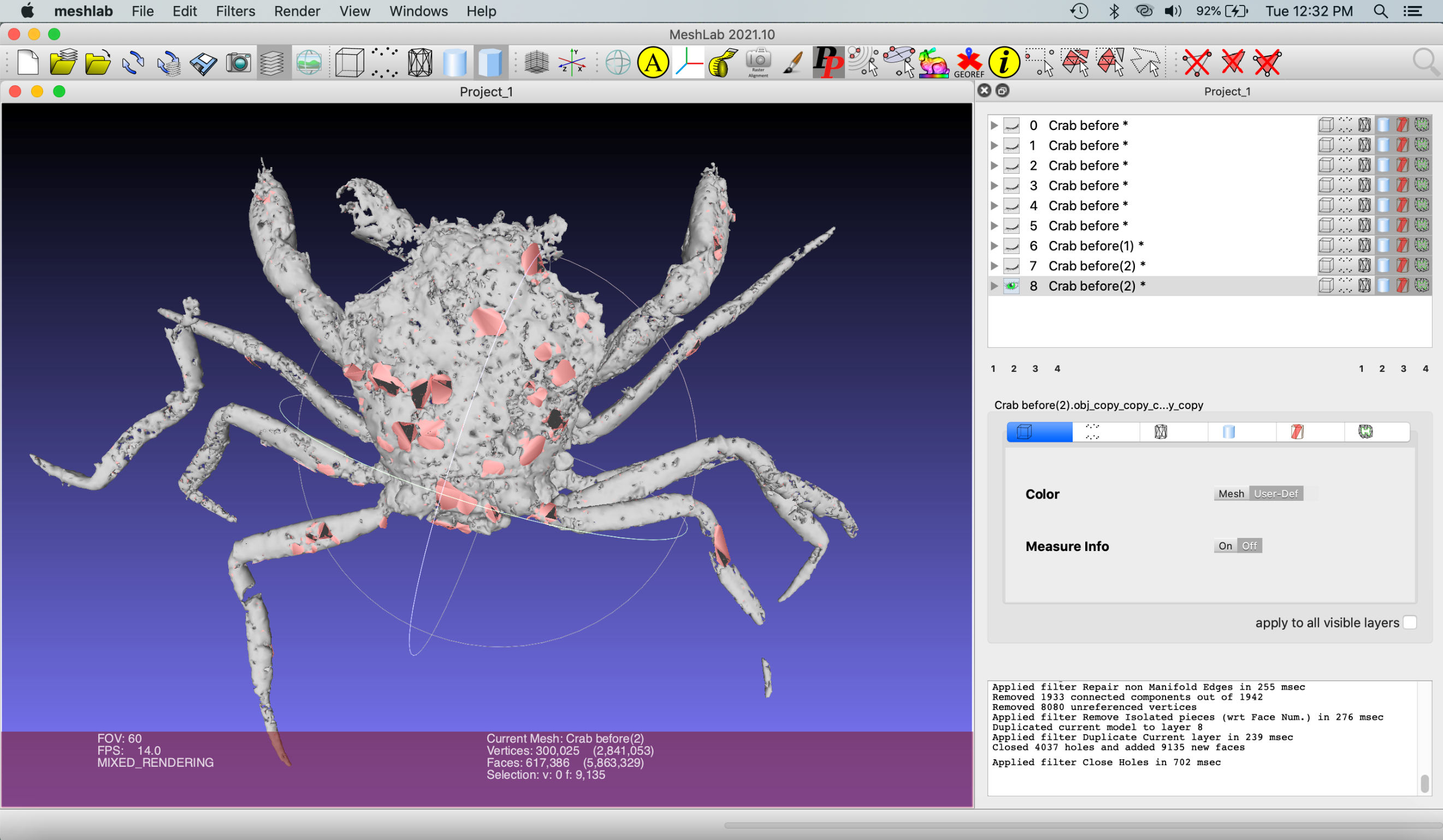


Deselect everything by selecting **Filters** > **Selection** > **Select None**.

*Shrinkwrap surface*

Now, we’re going to shrinkwrap a copy of the crab mesh to fill the leftover holes and smooth the surface of the crab. The first step to do this is to hollow out the inside of the mesh. Duplicate the mesh and select **Filters** > **Color Creation and Processing** > **Ambient Occlusion.** Keep default settings selected **click “okay” on the popup window**. This tags the external faces white and internal faces dark. Next, select **Filters** > **Selection** > **Select by Vertex Quality**. **Check preview selection**. **Move the sliders to set Min and Max** to optimize the highest number of internal faces and minimal external ones **(e.g., Min at 0 and Max at 0.132944)** (review cupule tutorial for details)**. Click “Apply.”**


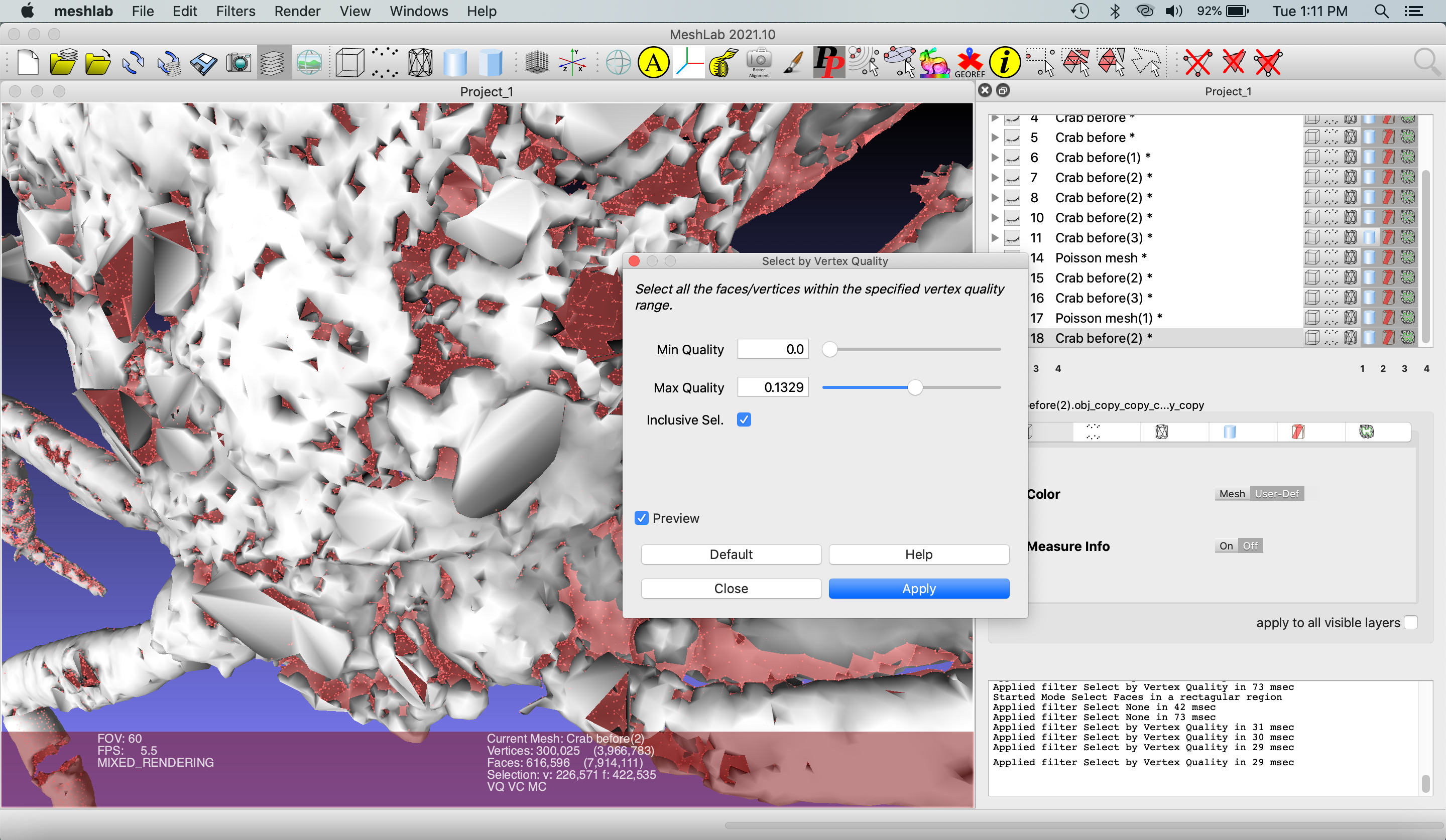


Select **Filters** > **Remeshing Simplification and Reconstruction** > **Surface Reconstruction: Screened Poisson**


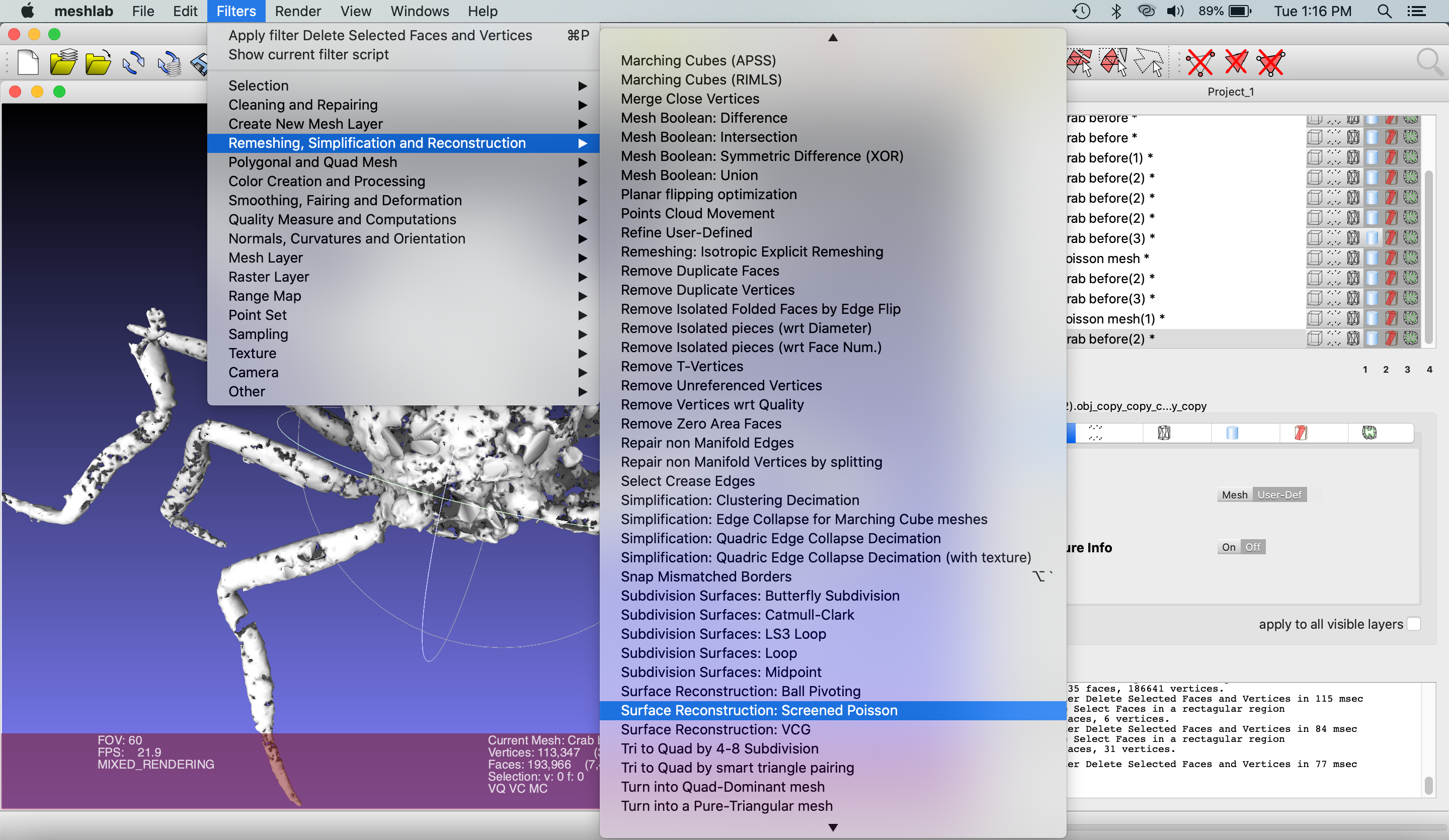


15. **Check “Pre-Clean”** and **click Apply**.


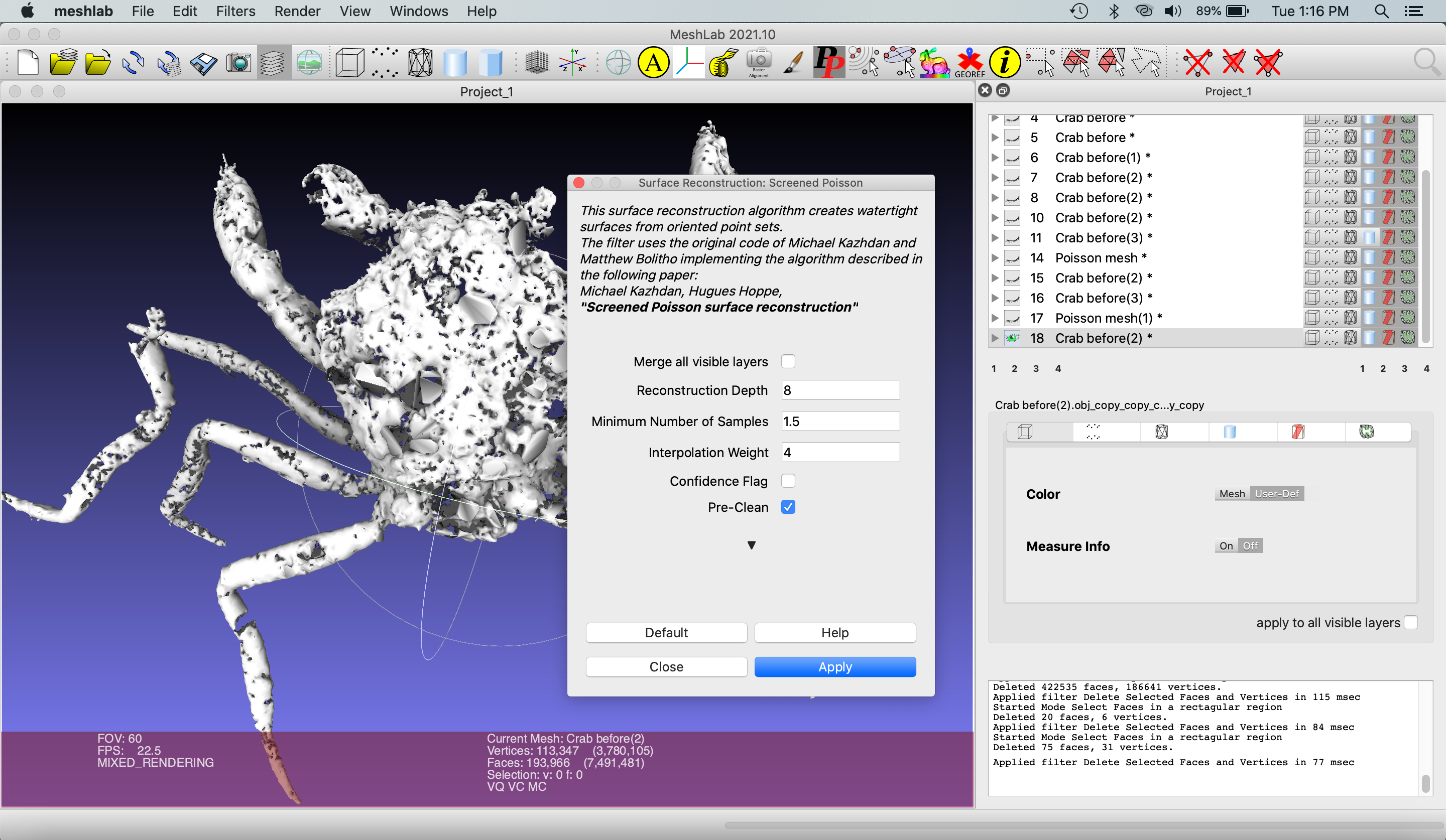


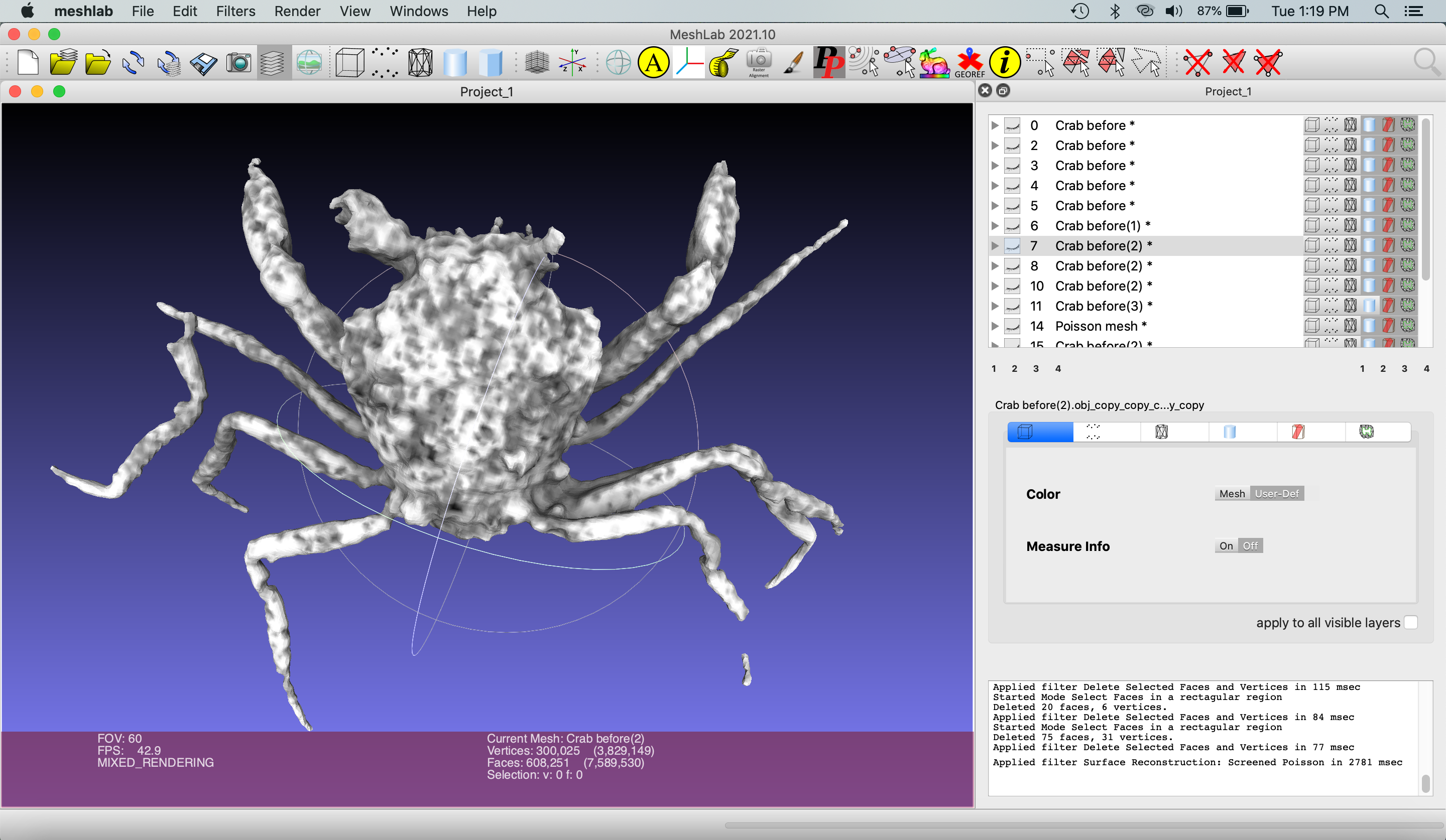


This new version of the mesh has filled holes and a smooth surface; however, some detail has been removed. Merge the new Poisson mesh with the mesh saved previously by making these two meshes visible and hiding any others by selecting the eye icon. **Right click one of the meshes and select “Flatten Visible Layers” to create a merged mesh**. Keep default settings selected.


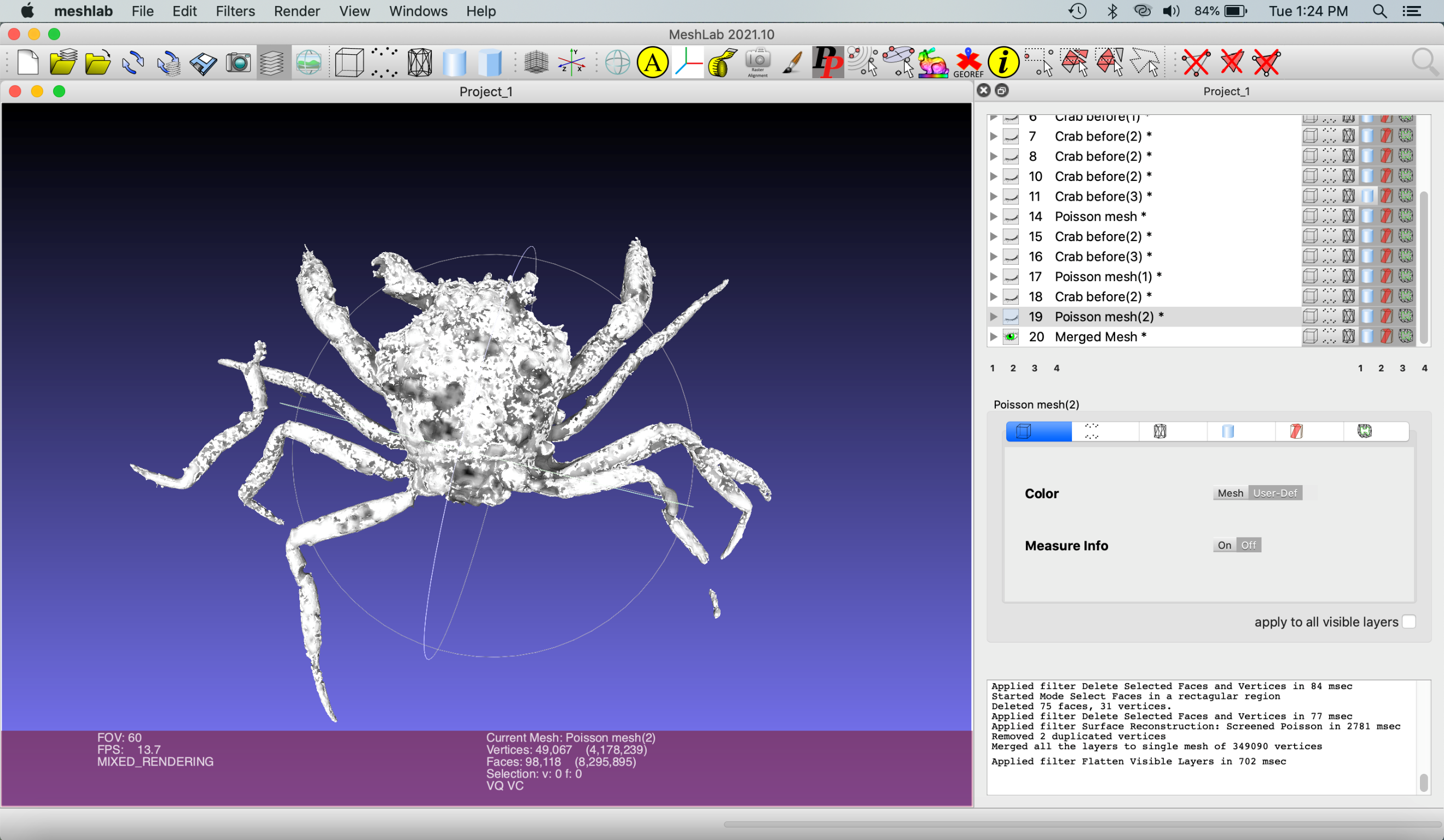


Save this mesh by selecting **File** > **Export Mesh**. Name the file “Body remesh.” A new pop-up window will appear. Select “none” in the bottom left corner and click “OK.”

*Repair Missing Features: Eye*

To simplify your scene and get rid of unwanted meshes, **close and restart Meshlab. Reopen Crab before.obj in Meshlab.**

Position the left eye of the crab at the center of the project window by **holding Command (Mac) or ctrl (PC) and dragging the mesh**, or holding down the scroll wheel and dragging on a mouse.


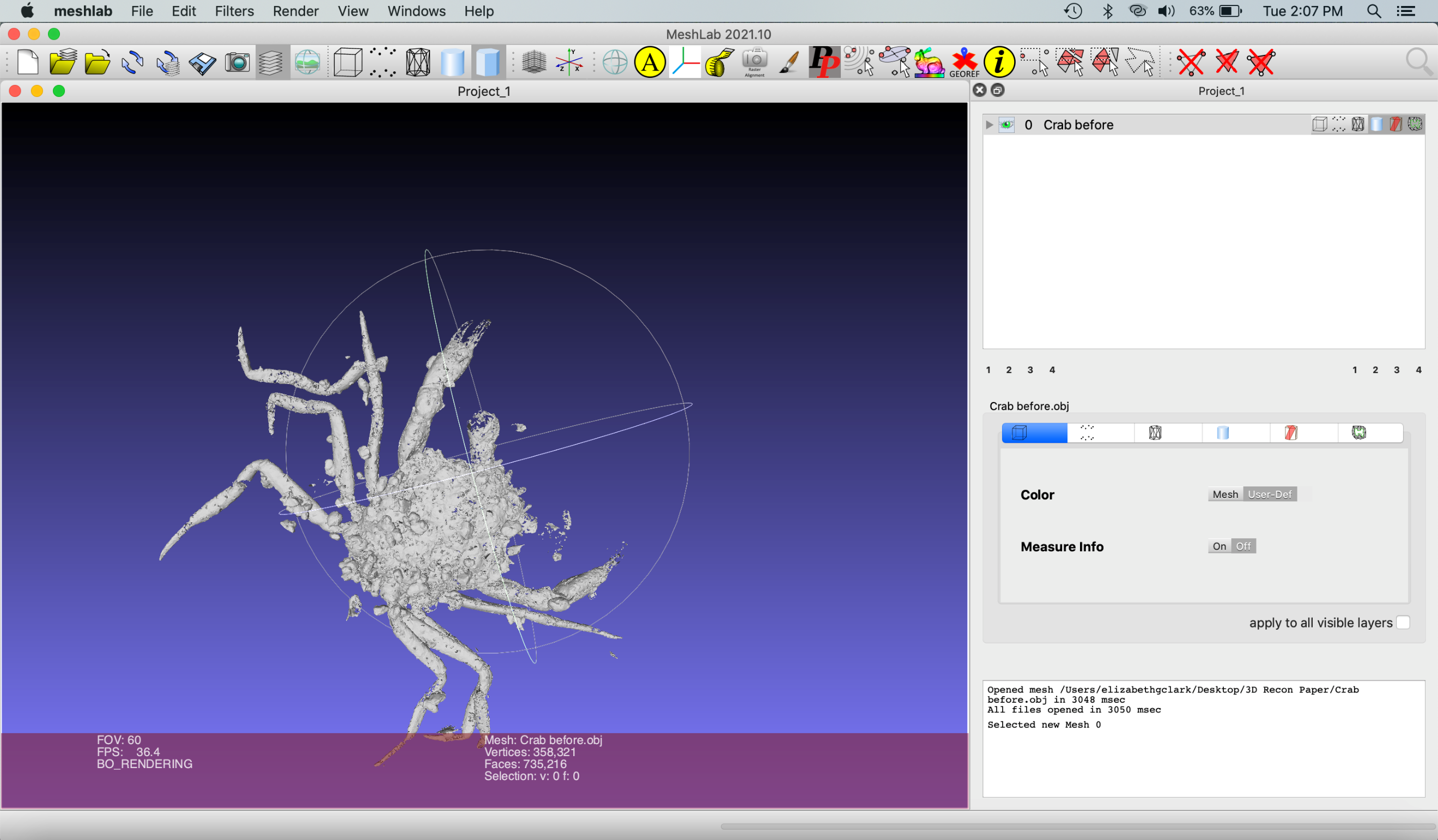


Zoom in using the scroll wheel on the mouse.

Select the eye using the Interactive Selection tool (
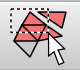
).


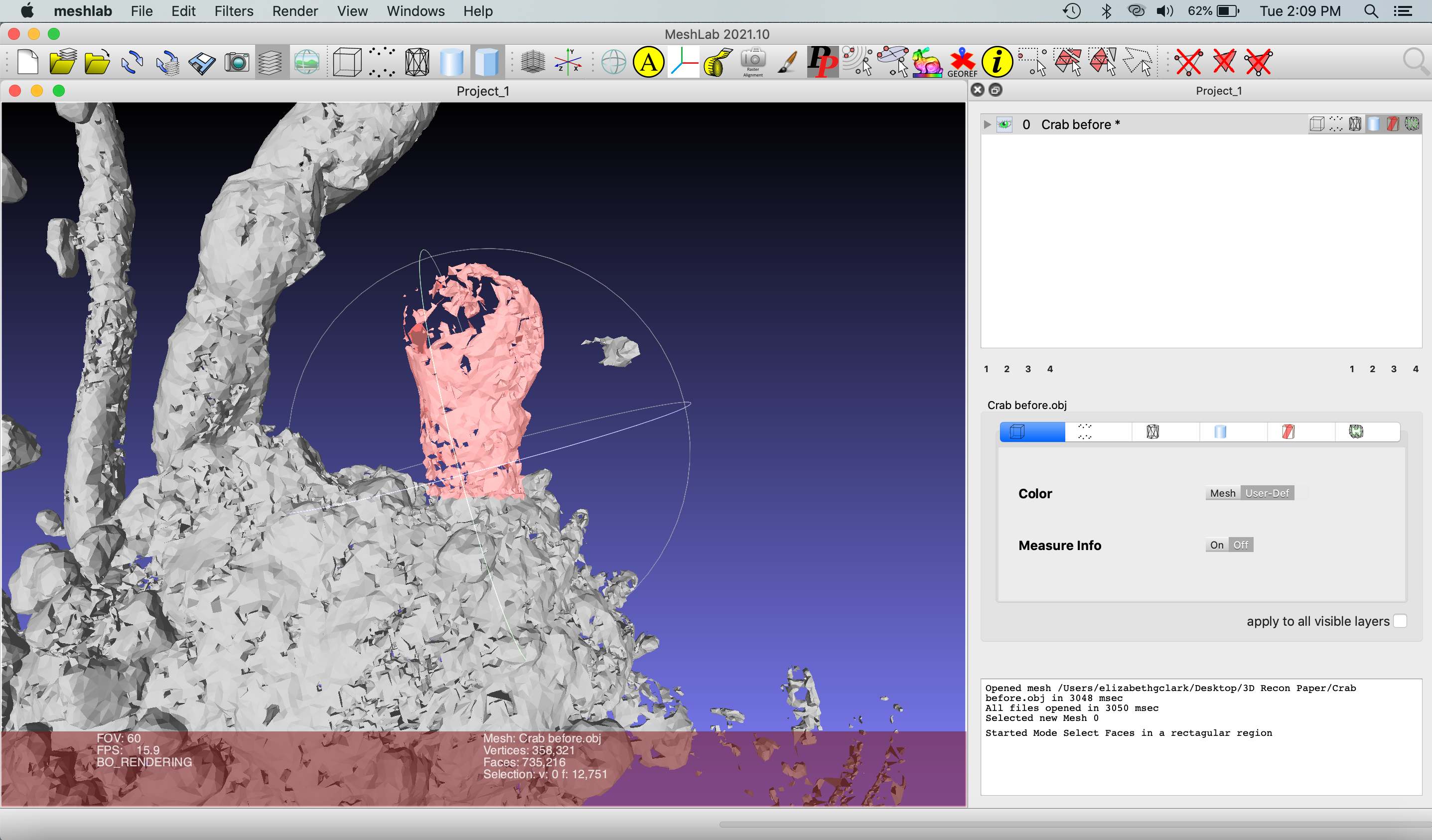


Select everything except for the eye by selecting **Filters** > **Selection** **> Invert selection**. **Check both Invert Faces and Invert Vertices, and click “Apply.”** All of the mesh components except those of the eye will be selected in red.


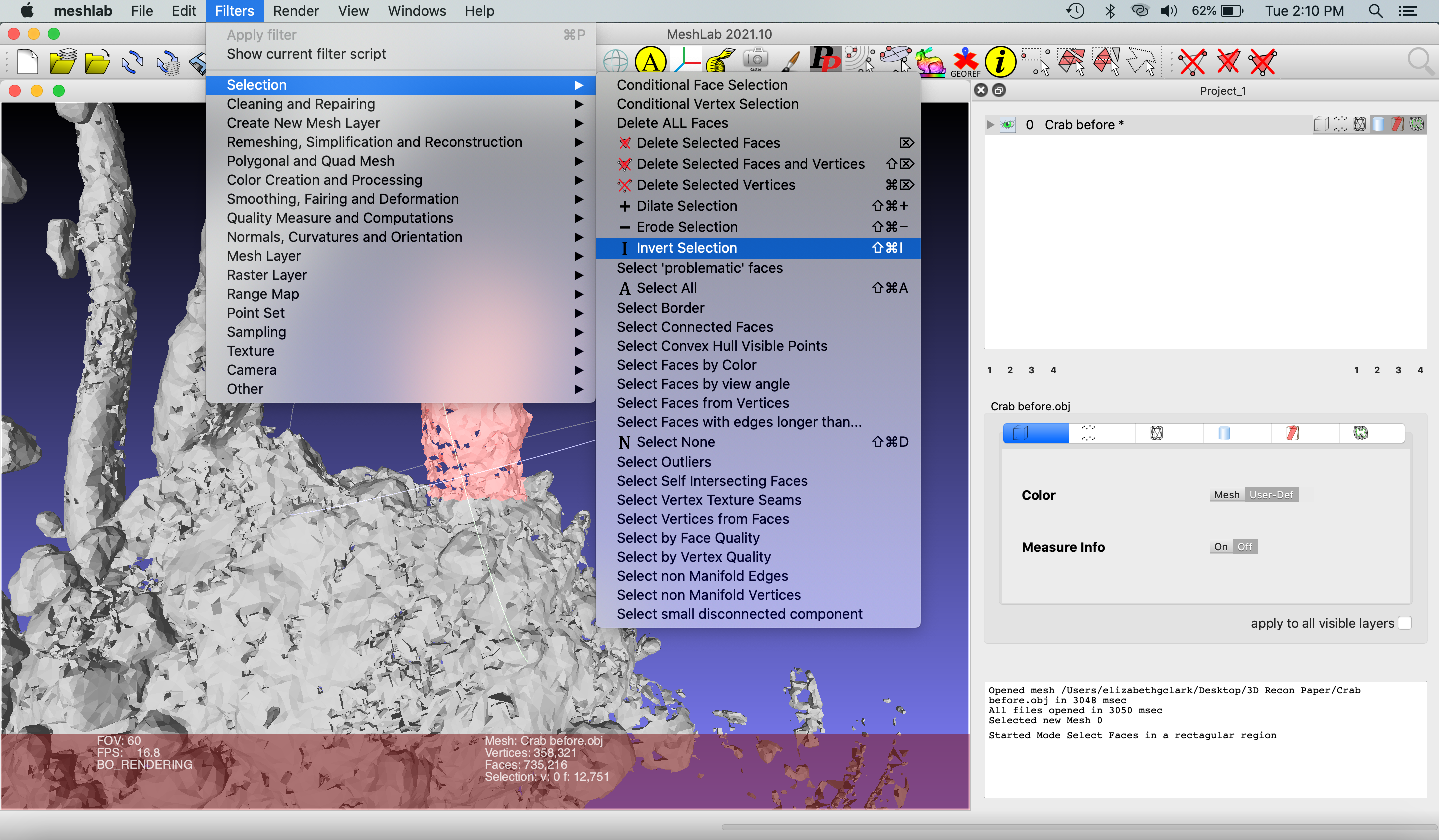


Delete selected faces and vertices (
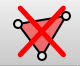
).

Shrinkwrap the eye. Perform the ambient occlusion filter and delete the internal vertices. Save this mesh by duplicating it. (See “Shrinkwrap surface” section for details)

Apply the Surface Reconstruction: Poisson Remesh. Enter 4 into the “Reconstruction Depth” window.

Merge the two meshes of the eye created here. Perform a Poisson Remesh of this new mesh: enter 5 into the “Reconstruction Depth” window. Export the file as “Eye remesh.”

*Repair Missing Features: Leg*

Close and restart Meshlab. Import and merge “Body remesh” and “Eye remesh”

Select the leg that's isolated from the rest of the body.

Copy the mesh.


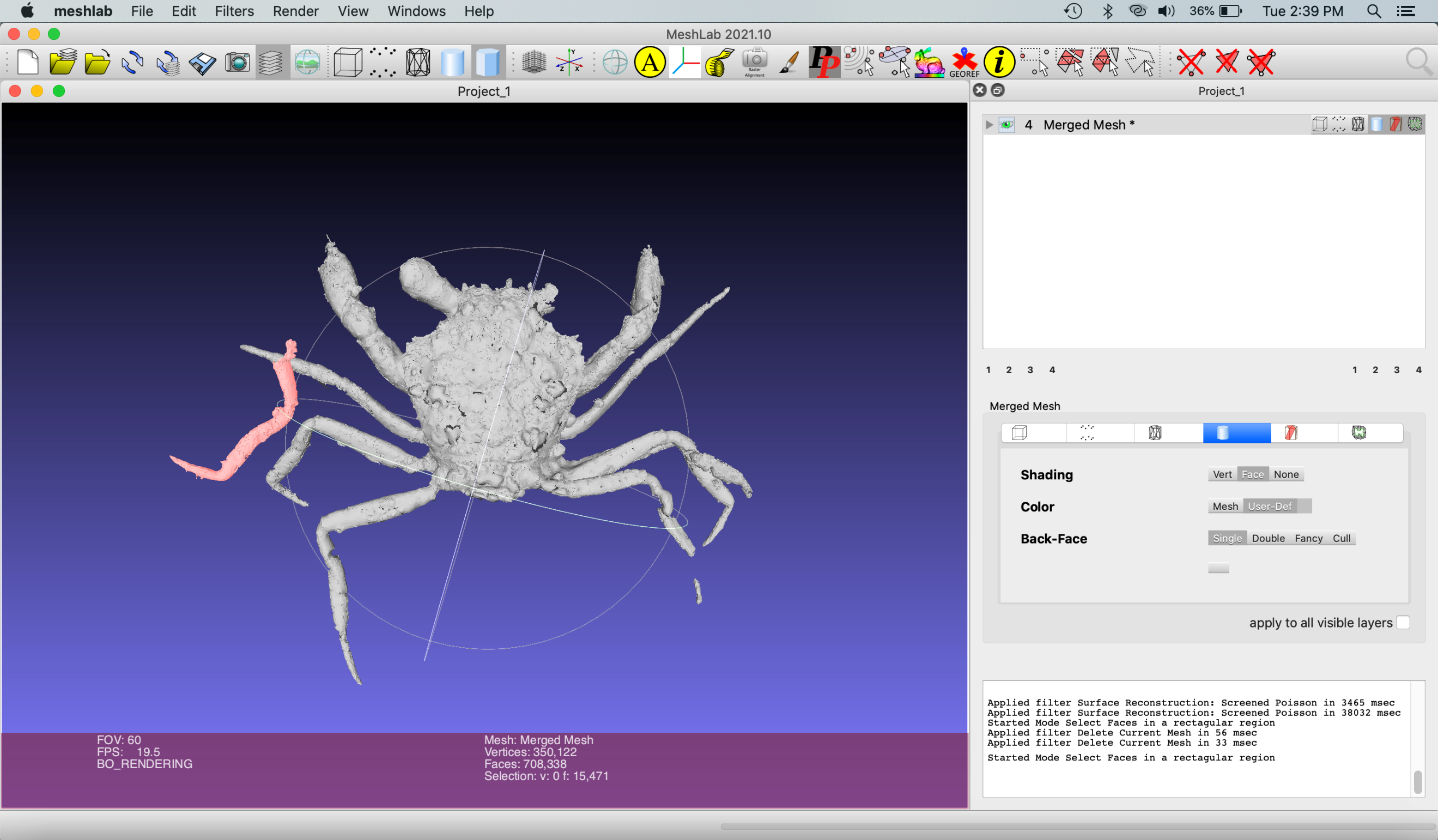


From the original mesh, invert the selection and delete the rest of the body.

Export this mesh “Leg remesh.”


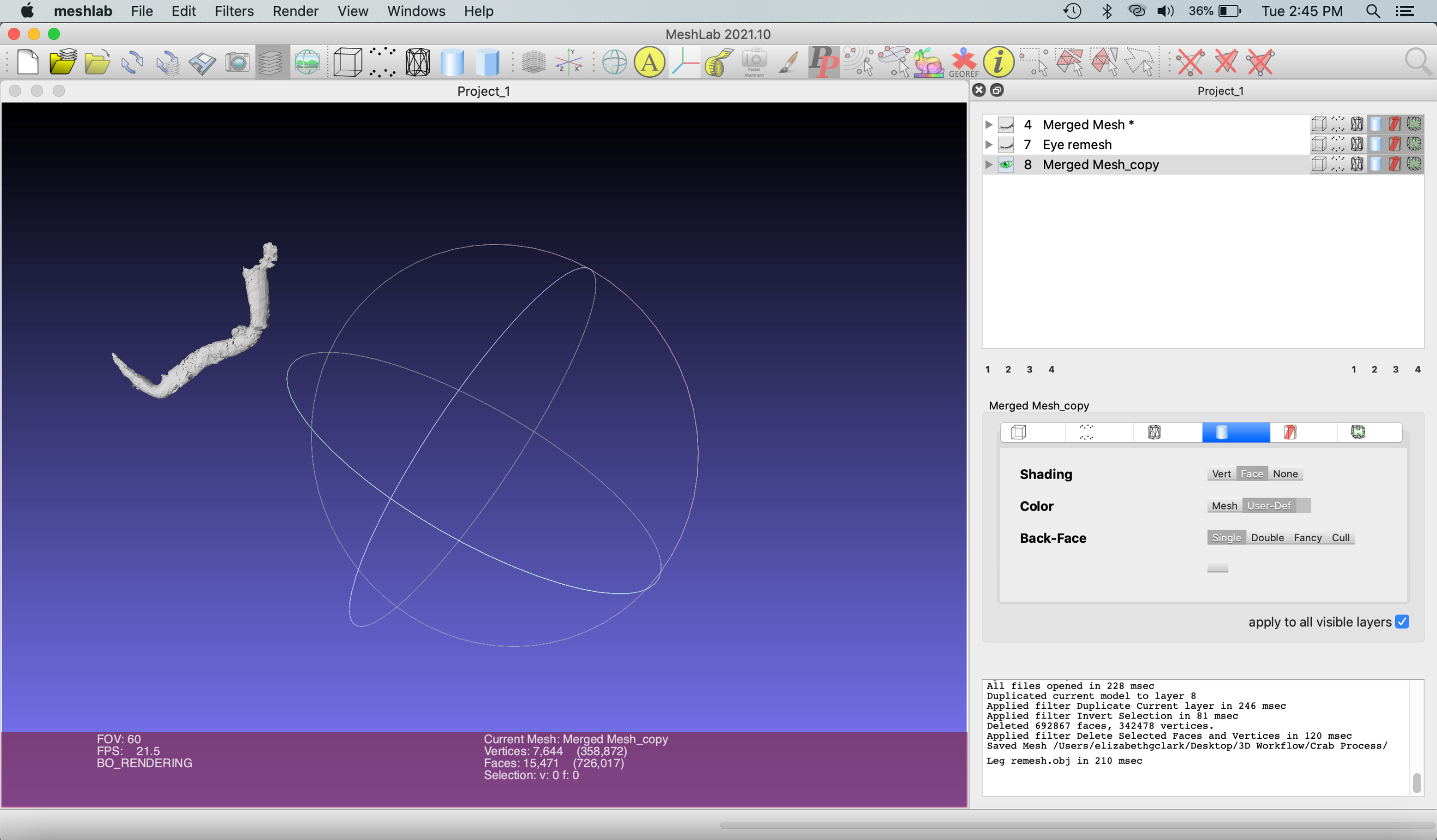


In the copy of the mesh, delete the leg. Export this mesh as “Crab body and eye remesh”


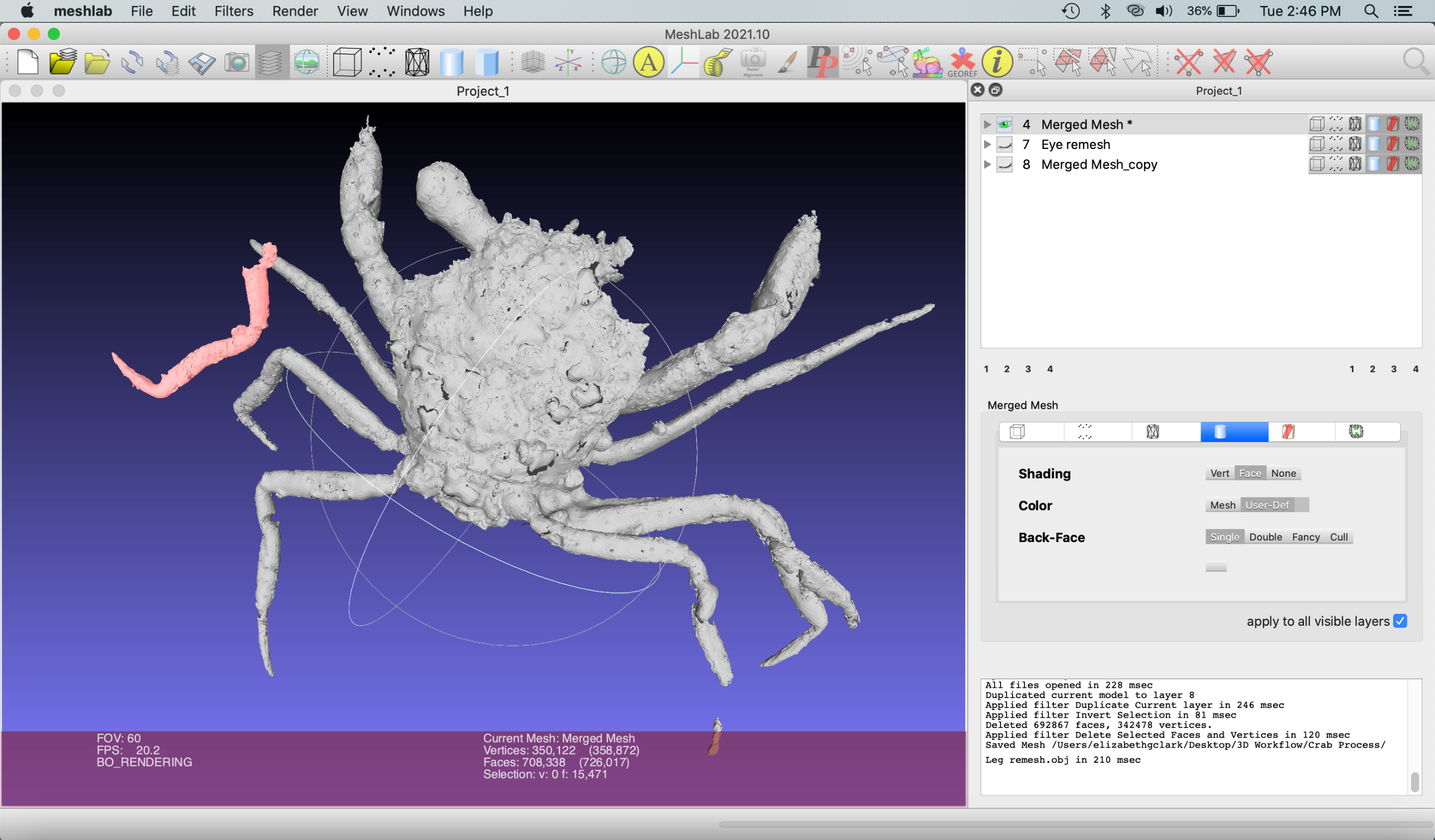


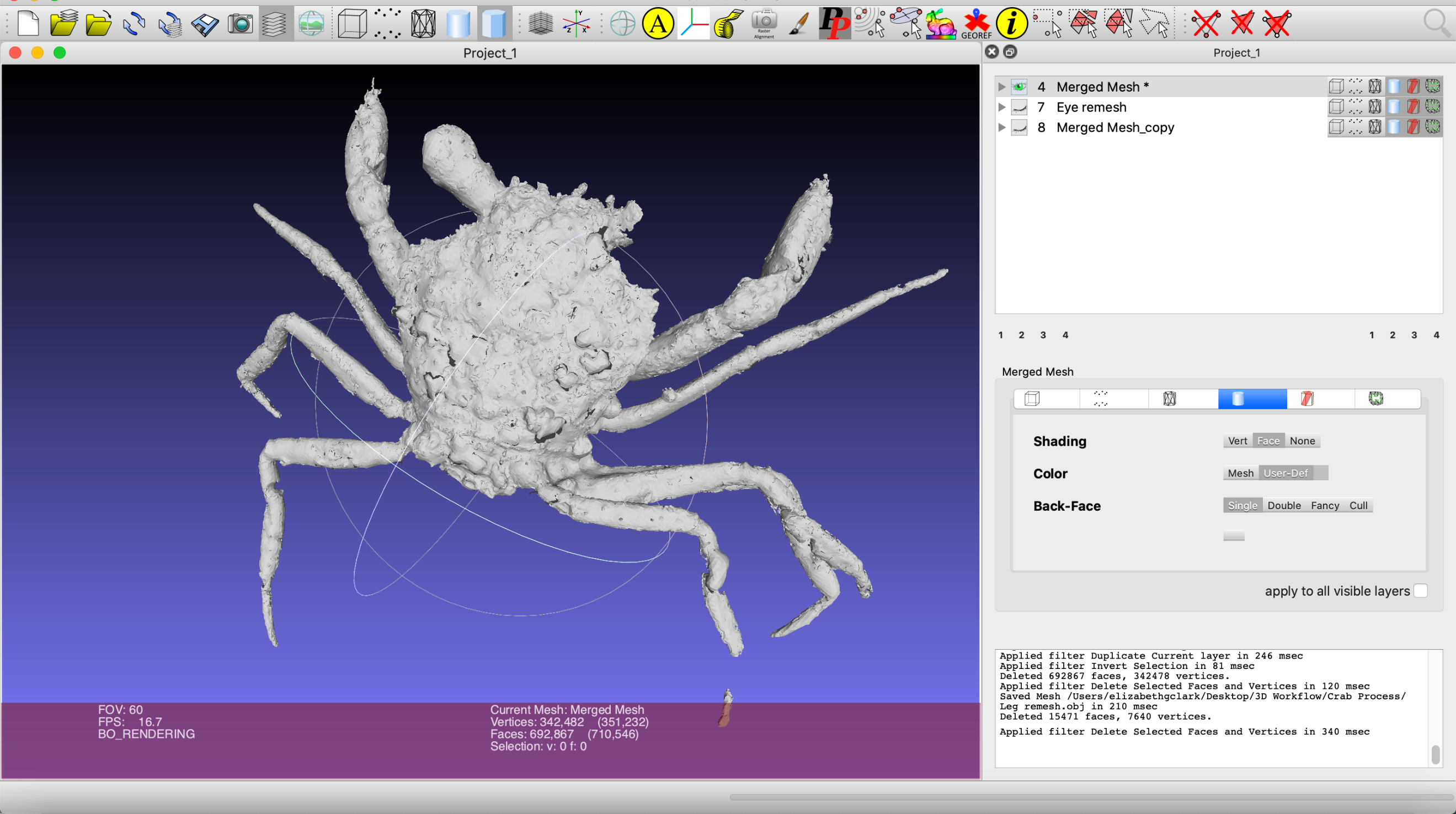


*Reposition and combine meshes*

Open Maya


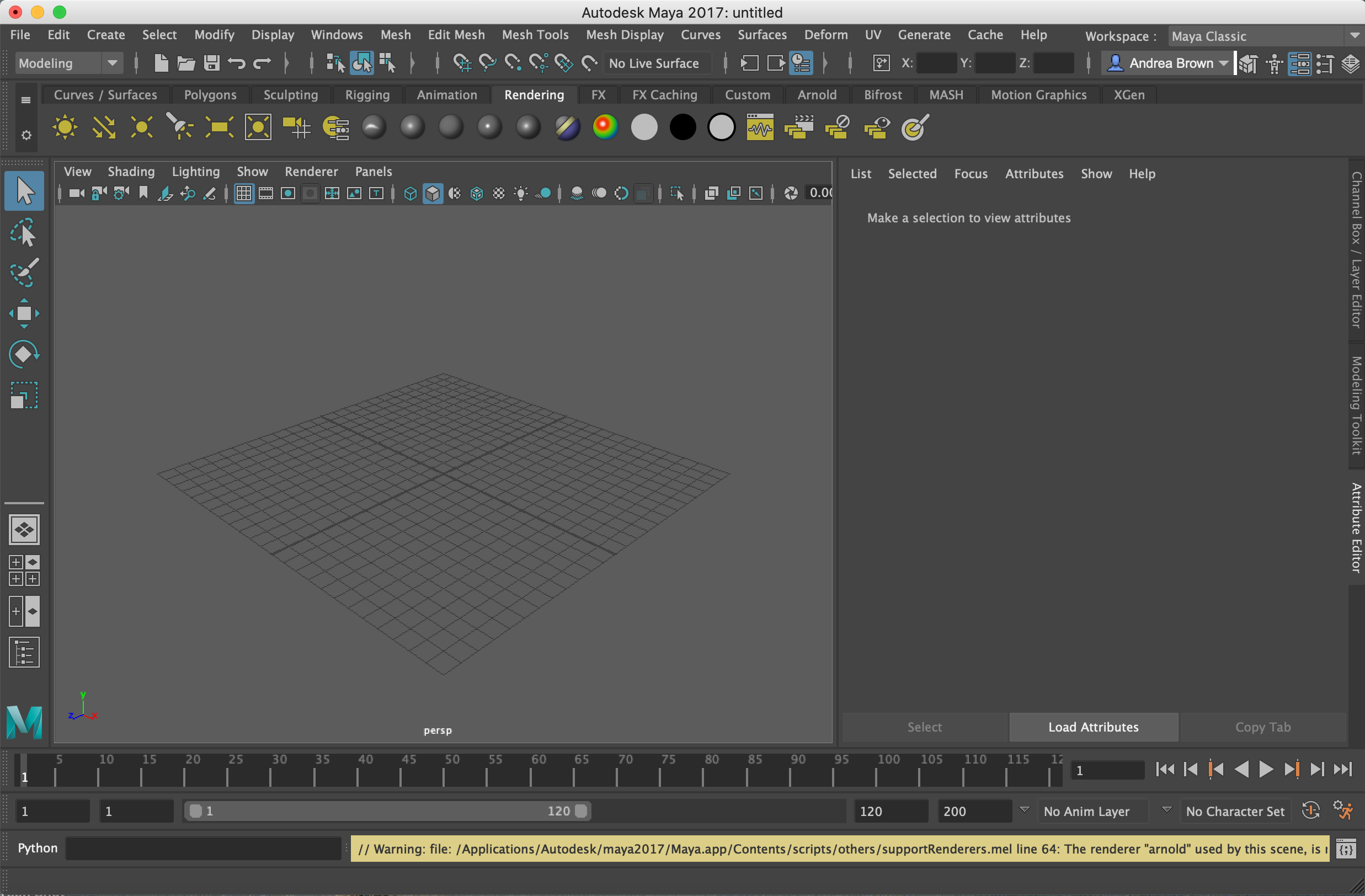


Import “Crab body and eye remesh.obj” and “Leg remesh.obj” into Maya by going to **File** > **Import** and selecting these files.


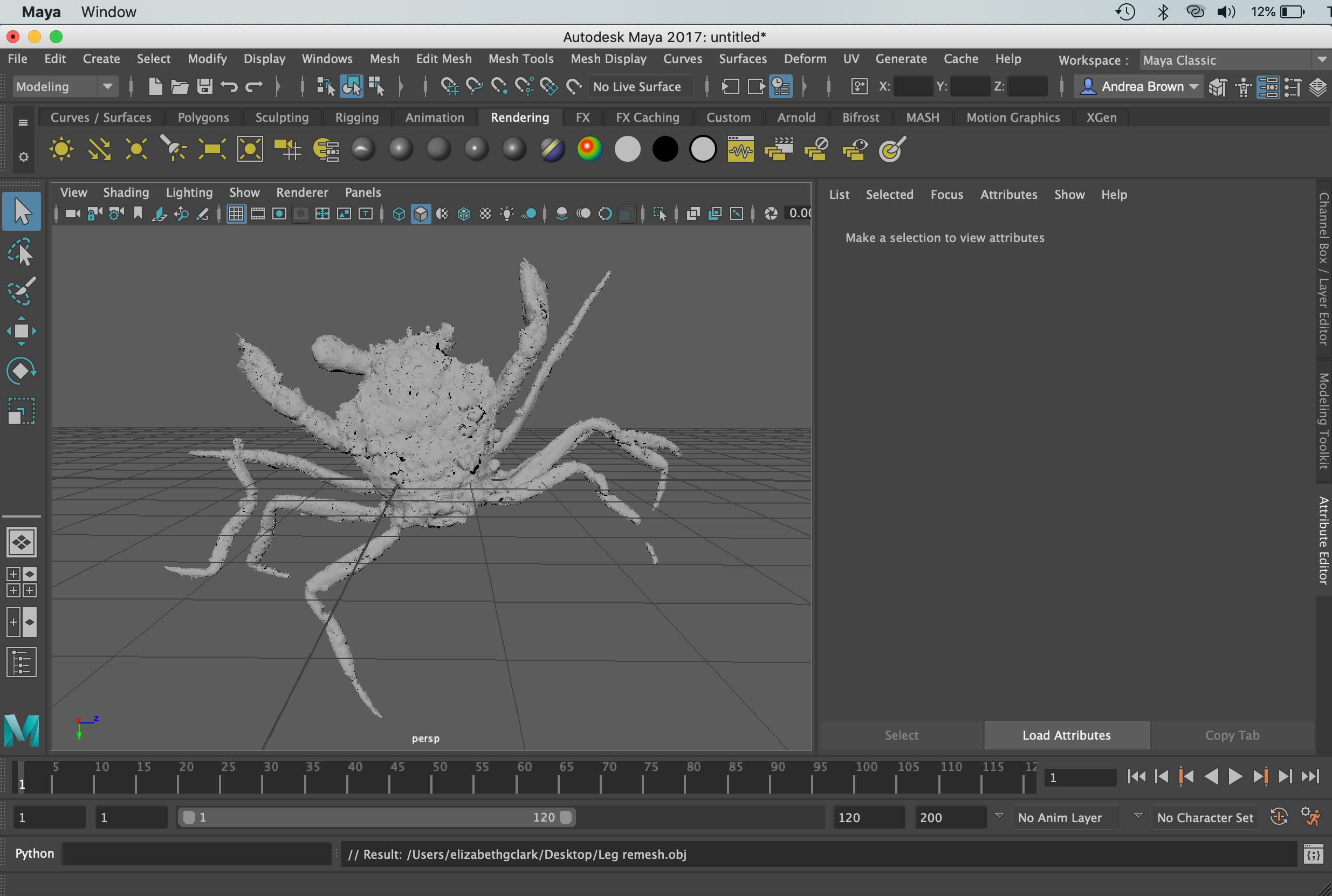


Click “A” to view all imported files. To rotate through the scene, hold “Alt” and left click while dragging the cursor.

On the lefthand side of the screen, there are tools for moving, rotating and scaling the objects within the scene.


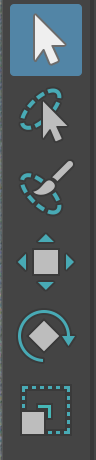


One of them is the transform tool:

And another is the rotation tool:

Click on the leg, and use these tools to transform and rotate the leg to reattach it to the body of the crab.

Now, we’re going to make a copy of the left eye to use as the right.

Reopen “Crab body and eye remesh.obj” in Meshlab.

Select the eye.

Invert selection and delete the body, leaving only the eye.

Export mesh as “Eye remesh 2.obj”

Import “Eye remesh 2.obj” into Maya.

To mirror the eye, first, select the scale icon on the left:

Click the icon to the right of the X, Y, Z in the first icon-based menu at the top of the screen and set to “Relative transform”

Type -1 into the Z box and push “Enter”

Select the newly mirrored eye and move to the correct location on the crab using the transform and rotate tools (hint: it helps to move the pivot by selecting the eye and selecting Modify – Center Pivot in the top menu).

Next, select all mesh components by clicking the selection icon, holding left click and dragging the cursor over all of the objects. Command A also works.

Select Mesh – Combine in the first menu at the top to merge all mesh components together.

Repeat the *Repair Missing Elements* protocol for the eyes to fix the tip of the claws and legs. Congratulations on making a beautiful crab!
