# Supplementary material for: Back to life: Techniques for developing high-quality 3D reconstructions of plants and animals from digitized specimens
Source: PLoS One. 2023 Mar 29;18(3):e0283027. doi: 10.1371/journal.pone.0283027 (PMC10058149; doi:10.1371/journal.pone.0283027)
Supplement: Attachment — Submitted filename: PONE-D-22-27354_reviewer_VDD.pdf [file pone.0283027.s002.pdf]

PLOS ONE

Back to life: Techniques for developing high-quality 3D reconstructions of plants and animals from digitized specimens

--Manuscript Draft--

**Manuscript Number:**	PONE-D-22-27354
**Article Type:**	Lab Protocol
**Full Title:**	Back to life: Techniques for developing high-quality 3D reconstructions of plants and animals from digitized specimens
**Short Title:**	Developing high-quality 3D reconstructions of plants and animals from digitized specimens
**Corresponding Author:**	Elizabeth G. Clark, Ph.D.<br>University of California Berkeley<br>Berkeley, CA UNITED STATES
**Keywords:**	Anatomy, functional morphology, 3D imaging, 3D reconstructions, 3D models, paleontology, vertebrate, arthropod, plant
**Abstract:**	Expanded use of 3D imaging in organismal biology and paleontology has substantially enhanced the ability to visualize and analyze specimens. These techniques have improved our understanding of the anatomy of many taxa, and the integration of downstream computational tools applied to 3D datasets have broadened the range of analyses that can be performed (e.g., finite element analyses, geometric morphometrics, biomechanical modeling). However, morphological analyses inevitably present challenges, particularly in fossil taxa where taphonomic or preservational artifacts distort and reduce the fidelity of the original morphology through shearing, compression, and disarticulation, for example. Here, we present a compilation of techniques to build high-quality 3D digital models of extant and fossil taxa from 3D imaging data using freely available software for students and educators. Our case studies and associated supplementary tutorials present instructions for working with reconstructions of plants and animals to directly address and resolve common issues with 3D imaging data. The strategies demonstrated here optimize scientific accuracy and computational efficiency and can be applied to a broad range of taxa.
**Order of Authors:**	Elizabeth G. Clark, Ph.D.
	Kelsey M. Jenkins, M. Sc.
	Craig R. Brodersen, Ph.D.
**Additional Information:**	
**Question**	**Response**
**Financial Disclosure**<br><br>Enter a financial disclosure statement that describes the sources of funding for the work included in this submission. Review the submission guidelines for detailed requirements. View published research articles from *PLOS ONE* for specific examples.<br><br>This statement is required for submission and **will appear in the published article** if the submission is accepted. Please make sure it is accurate.	The author(s) received no specific funding for this work.

Powered by Editorial Manager® and ProduXion Manager® from Aries Systems Corporation

**Unfunded studies**<br>Enter: *The author(s) received no specific funding for this work.*<br><br>**Funded studies**<br>Enter a statement with the following details:<br>• Initials of the authors who received each award<br>• Grant numbers awarded to each author<br>• The full name of each funder<br>• URL of each funder website<br>• Have or will the sponsors or funders play any role in the study design, data collection (when applicable) and analysis, decision to publish, or preparation of the manuscript?<br>• **NO** - Include this sentence at the end of your statement: *The funders had and will not have a role in study design, data collection and analysis, decision to publish, or preparation of the manuscript.*<br>• **YES** - Specify the role(s) played.<br><br>* typeset	
**Competing Interests**<br><br>Use the instructions below to enter a competing interest statement for this submission. On behalf of all authors, disclose any competing interests that could be perceived to bias this work—acknowledging all financial support and any other relevant financial or non-financial competing interests.<br><br>This statement is required for submission and **will appear in the published article** if the submission is accepted. Please make sure it is accurate and that any funding sources listed in your Funding Information later in the submission form are also declared in your Financial Disclosure statement.<br><br>View published research articles from *PLOS ONE* for specific examples.	The authors have declared that no competing interests exist.

**NO authors have competing interests**<br><br>Enter: *The authors have declared that no competing interests exist.*<br><br>**Authors with competing interests**<br><br>Enter competing interest details beginning with this statement:<br><br>*I have read the journal's policy and the authors of this manuscript have the following competing interests: [insert competing interests here]*<br><br>* typeset	
**Ethics Statement**<br><br>Enter an ethics statement for this submission. This statement is required if the study involved:<br><br>• Human participants<br>• Human specimens or tissue<br>• Vertebrate animals or cephalopods<br>• Vertebrate embryos or tissues<br>• Field research<br><br>Write "N/A" if the submission does not require an ethics statement.<br><br>General guidance is provided below. Consult the submission guidelines for detailed instructions. **Make sure that all information entered here is included in the Methods section of the manuscript.**	N/A

Powered by Editorial Manager® and ProduXion Manager® from Aries Systems Corporation

**Format for specific study types**<br><br>**Human Subject Research (involving human participants and/or tissue)**<br>• Give the name of the institutional review board or ethics committee that approved the study<br>• Include the approval number and/or a statement indicating approval of this research<br>• Indicate the form of consent obtained (written/oral) or the reason that consent was not obtained (e.g. the data were analyzed anonymously)<br><br>**Animal Research (involving vertebrate animals, embryos or tissues)**<br>• Provide the name of the Institutional Animal Care and Use Committee (IACUC) or other relevant ethics board that reviewed the study protocol, and indicate whether they approved this research or granted a formal waiver of ethical approval<br>• Include an approval number if one was obtained<br>• If the study involved *non-human primates*, add *additional details* about animal welfare and steps taken to ameliorate suffering<br>• If anesthesia, euthanasia, or any kind of animal sacrifice is part of the study, include briefly which substances and/or methods were applied<br><br>**Field Research**<br><br>Include the following details if this study involves the collection of plant, animal, or other materials from a natural setting:<br>• Field permit number<br>• Name of the institution or relevant body that granted permission	
**Data Availability**<br><br>Authors are required to make the data underlying their research findings fully available, without restriction.<br><br>If the manuscript reports sample data generated using the protocol, the data underlying those results must be made available at the time of publication. A Data	Yes - sample data are fully available without restriction

Availability Statement describing where the data can be found is required at submission. When legal or ethical requirements prohibit public data sharing, authors must indicate how researchers may obtain access to the data. See the PLOS Data Policy and FAQ for detailed information.<br><br>A Data Availability Statement describing where the data can be found is required at submission. Your answers to this question constitute the Data Availability Statement and **will be published in the article**, if accepted.<br><br>**Important:** Stating 'data available on request from the author' is not sufficient. If your data are only available upon request, select 'No' for the first question and provide justification in the text box.<br><br>For protocols without sample data or results, you must state this in your Data Availability Statement.<br><br>Do the authors confirm that EITHER the protocol does not report results OR that sample data will be made available?	
**Describe where the data may be found in full sentences. If you are copying our sample text, replace any instances of XXX with the appropriate details.**<br><br>• If the data are **held or will be held in a public repository**, include URLs, accession numbers or DOIs. If this information will only be available after acceptance, indicate this by ticking the box below. For example: *All XXX files are available from the XXX database (accession number(s) XXX, XXX.*).<br>• If the data are all contained **within the manuscript and/or Supporting Information files**, enter the following: *All relevant data are within the*	Data is available as part of the Supplemental Materials and from Figshare: 10.6084/m9.figshare.21266568<br><br>Please note that the data is available for review through this link: https://figshare.com/s/99f6d615c534187f5350

manuscript and its Supporting Information files.<br>• If neither of these applies but you are able to provide **details of access elsewhere**, with or without limitations, please do so. For example:<br><br>*Data cannot be shared publicly because of [XXX]. Data are available from the XXX Institutional Data Access / Ethics Committee (contact via XXX) for researchers who meet the criteria for access to confidential data.*<br><br>*The data underlying the results presented in the study are available from (include the name of the third party and contact information or URL).*<br>• This text is appropriate if the data are owned by a third party and authors do not have permission to share the data.<br><br>* typeset	
Additional data availability information:	Tick here if the URLs/accession numbers/DOIs will be available only after acceptance of the manuscript for publication so that we can ensure their inclusion before publication.

Manuscript Main Text
Click here to access/download;Manuscript;3D Recon Main Text Submitted.docx

Title: Back to life: Techniques for developing high-quality 3D reconstructions of plants and animals from digitized specimens

Authors: Elizabeth G. Clark[1], Kelsey M. Jenkins[2], Craig R. Brodersen[3]

1. Department of Environmental Science, Policy and Management, University of California Berkeley, Berkeley CA, 94720
2. Department of Earth and Planetary Sciences, Yale University, New Haven CT, 06511
3. School of the Environment, Yale University, New Haven CT, 06511

Abstract

Expanded use of 3D imaging in organismal biology and paleontology has substantially enhanced the ability to visualize and analyze specimens. These techniques have improved our understanding of the anatomy of many taxa, and the integration of downstream computational tools applied to 3D datasets have broadened the range of analyses that can be performed (e.g., finite element analyses, geometric morphometrics, biomechanical modeling). However, morphological analyses inevitably present challenges, particularly in fossil taxa where taphonomic or preservational artifacts distort and reduce the fidelity of the original morphology through shearing, compression, and disarticulation, for example. Here, we present a compilation of techniques to build high-quality 3D digital models of extant and fossil taxa from 3D imaging data using freely available software for students and educators. Our case studies and associated supplementary tutorials present instructions for working with reconstructions of plants and

animals to directly address and resolve common issues with 3D imaging data. The strategies demonstrated here optimize scientific accuracy and computational efficiency and can be applied to a broad range of taxa.

Introduction

The use of three-dimensional (3D) imaging techniques, particularly computed tomography (CT), has become widespread in biology and paleontology within the last decade (e.g., Sutton et al., 2017; Bicknell et al. 2018; Luque et al. 2021). One of the primary advantages of many 3D methods is their non-destructive nature that allows the user to digitally dissect the sample into thousands of virtual serial sections, which can then be viewed using computer visualization software to explore the sample beyond the traditional orthogonal planes available with light microscopy. CT imaging also permits the visualization of internal and external anatomical features as they are arranged *in situ*, which can be useful for interpreting function and behavior (e.g., Brainerd et al. 2010; Brodersen et al. 2011; Clark et al. 2018; Wason et al. 2019). CT imaging can be especially useful in the visualization of fossil taxa that may be embedded within a rocky matrix (e.g., Clark et al. 2020 a,b; Shi et al. 2021).

Using 3D image processing software, CT image data can be used to create a 3D reconstruction of a fossil or extant specimen. This is done by manually or automating the selection of **voxels** (three-dimensional pixels) that designate the specimen in the 3D image. The shape of the selected voxels can then be used to generate a **mesh** (a digital polyhedron) comprised of a series of triangular **faces** made of points (**vertices**) connected by line segments (**edges**) formed about the surface of the selected voxels (Fig

1). This mesh can be exported from the 3D image processing software for subsequent analyses. In studies of functional morphology, 3D reconstructions integrating or informed by CT imaging can be used in downstream analyses such as geometric morphometrics (e.g., Hopkins and Pearson 2016), finite element analyses (Rayfield 2007), biomechanical analyses (e.g., Clark et al. 2018, Molnar et al. 2021), and fluid dynamics simulations (e.g., Brodersen 2013; Rahman et al. 2015; Darroch et al. 2017).

Fig 1. (A) 3D reconstruction of a left femur from *Equus* sp. (UF 234402) downloaded from morphosource.org and visualized using Meshlab. Specimen demonstrates several preservational issues commonly encountered while working with 3D imaging data (labeled). (B) View of the mesh components that comprise the 3D reconstruction, which includes (C) edges, vertices and faces.

However, digital volumes of whole specimens or even isolated anatomical structures directly extracted from 3D imaging data are generally suboptimal for surface visualization and downstream analyses due to structural inaccuracies which can be acquired during the processes of preservation, sample preparation, and mesh creation. During preservation, samples can incur structural damages which 3D digital visualizations alone cannot rectify. Sample preparation can also integrate structural inaccuracies in 3D visualization. The generation of a 3D volume with CT scanning relies on a density contrast between the sample or anatomical features of interest and their surroundings; objects embedded within material of similar densities, for instance, can have low contrast and

result in a 3D volume of low quality. Many meshes generated from 3D visualization software can be extremely large and require simplification due to computational limitations, which can remove detail. Sculpting a model via manual translation of vertices, edges and faces of a simpler shape has become a common way to circumvent these issues (Rahman et al. 2015, 2020; Rahman and Lautenschlager 2017; Darroch et al. 2017; Morón-Alfonso et al. 2020; Liu et al. 2021).

Ideally, however, 3D reconstructions would directly incorporate as much data from the original digitized specimen as possible. Field-specific techniques have been developed to reconstruct damaged or missing anatomical components based on known morphologies of similar, closely related fossil taxa with few taphonomic artifacts (Zollikofer, 2002; Zollikofer and Ponce de León, 2005; Gunz et al., 2009; Degrange et al., 2010; Grine et al., 2010; Molnar et al., 2012; Young et al., 2012; Cunningham et al., 2014; Lautenschlager 2016; Chambi-Trowell et al., 2020; Pintore et al. 2022; DeVries et al. 2022). However, every digitized specimen comes with unique anatomy and its own set of structural inaccuracies. The goal of this paper is to provide a platform for developing customizable workflows to build an optimal 3D digital reconstruction for specimens across taxonomic boundaries. Using 3D editing software that is freely available for students and for educational and research purposes, we present a compilation of strategies for repairing common taphonomic and preservational artifacts in 3D meshes of digitized extant and fossil specimens that can be applied broadly across plants and animals. These workflows are designed to create 3D models from original specimen data and improve the fidelity of 3D digital reconstructions of a wide range of specimens. We include three case studies using plant, vertebrate, and arthropod specimens, respectively, which

include detailed tutorials for user learning and practice (Supplemental Information). . Elements of the workflows presented here have been employed to build 3D reconstructions of specimens for several studies (e.g., Clark et al. 2020a; Luque et al. 2021; Shi et al. 2021).

A 3D reconstruction is, by definition, a representation of a physical specimen. While repair and touch-ups of specimens may obscure original data, a carefully constructed model can often better convey the organism as it was *in vivo* and enable computationally intensive downstream analyses. This enables the application of digital technology to study organisms in a new way, opening doors to illuminate previously unanswerable questions in organismal and evolutionary biology, as well as enabling digital data dissemination and promoting accessibility.

Material and Methods

Common challenges in the study of digitized specimens can include the presence of external debris, shearing and compression, disarticulated, broken or missing elements, irregular surfaces and rough edges, large file size, and data storage (Fig 1A). Digitally correcting structural problems within each dataset will require a detailed understanding of an intact specimen to guide the process. Here, we present a collection of strategies for addressing these issues and demonstrate their use through Case Studies 1, 2 and 3 (see Supplementary Material).

Removing external debris: The presence of external debris can hinder the digital visualization and preparation of both fossil and extant specimens. 3D imaging requires

that the specimen is immobilized for the duration of the scan. This typically includes mounting the specimen in foam, plastic, or another low-density material. While it is a necessary step in data acquisition, the material used for specimen containment can be captured in the reconstructed image files, particularly when conducting extremely high-resolution imaging, such as synchrotron-based tomography. Furthermore, extracted volumes from 3D images of fossil specimens often retain portions of rock matrix. The removal of external debris can be incredibly time-consuming when performed manually; however, aspects of this process can be automated. Because digitized fossil elements are often one or several connected meshes, removing isolated mesh elements smaller than the primary mesh of the specimen can be an effective way to remove extraneous elements in bulk (demonstrated in Case Study 3). For noise represented by larger meshes, faces and vertices can be manually selected and deleted (demonstrated in Case Study 3). Extraneous faces connected to the desired object can also be smoothed out or removed (demonstrated in Case Study 2 and 3).

Patching holes: At times, holes in the polygon mesh representing the surface structure resulting from taphonomic processes are present in fossils, or even in modern specimens that have missing or broken elements pre- or post-mortem. Following the rearticulation of disassociated elements (as demonstrated in Case Study 3), a secondary mesh (i.e., a **patch**) can be sized and sculpted in a modeling software to repair holes in the digital specimen (demonstrated in Case Study 2). Depending on subsequent analyses taking place using the specimen, the user may need to combine the fossil mesh and patch by

shrinkwrapping by digitally creating a new simplified mesh using the position of the vertices at the surface of the original object. This process is outlined in Case Study 2.

Shearing and compression: Diagenetic processes can result in shearing or compression of fossil specimens (e.g., Ogihara et al., 2006; Arbour and Currie, 2012). However, in certain cases, planes of symmetry can be inferred to provide guidance for reconstructing damaged areas. Deshearing or decompression of 3D object files can be performed by editing selected faces, vertices and/or edges via translation or scaling. This approach can allow the user to select a specific volume of tissue that is broken or compressed and then manipulate those areas such that they can be restored to match the surrounding unaffected tissue. The appropriate degree of deshearing or decompressing to be performed on a 3D object file can be difficult to estimate. For many taxa, the assumption of bilaterality is appropriate, and deshearing can be carried out over an observed or calculated plane of symmetry (DeVries et al. 2022). In other cases, an examination of multiple specimens preserved in different orientations can provide guidance regarding the degree of deshearing or decompression to be applied. In either case, prior knowledge of the taxa or group is necessary to inform these reconstructions, and any editing performed must be justified and detailed through accessible documentation. Deshearing and decompression can be performed using the mesh sculpting tools described in Case Study 2.

Disarticulated elements: Particularly with fossils, preserved elements of the specimen may often be broken or disarticulated. Individual elements can be exported separately

from the reconstructed data in the original 3D visualization software (e.g. Avizo, VGStudio) or in a mesh editing software (e.g., Meshlab). These isolated elements can then be positioned in a program for modeling or rigging (e.g., Autodesk Maya). The proper positioning can be inferred from modern analogues, anatomical inference, examination of other specimens, or a symmetrical element can be mirrored if a plane of symmetry exists in the organism. Rearticulation of elements is demonstrated in Case Study 3.

Missing features: If bilaterality can be assumed, missing elements can be created from a symmetrical element. The element can be isolated in a mesh editing software (e.g., Meshlab) and copied, reflected and positioned in a modeling/rigging program (e.g., Autodesk Maya). This method is outlined in Case Study 3.

Irregular surfaces: Many 3D meshes are extracted from 3D imaging software with uninformative internal structures and irregular surfaces, rendering the files large and poorly optimized for surface visualization. Additionally, the digitization process of certain specimens or anatomical features may inaccurately interpret and present a solid object as a series of floating, isolated faces or vertices (e.g., the left crab claw in Case Study 3). Individual faces and vertices can be manually removed in a mesh editing software (Case Study 3). They can also be softened by translating individual or groups of faces, edges, or vertices in a modeling program (Case Study 2). Prominent edges from lower-resolution polygon meshes can be smoothed in a mesh editing software using a mesh subdivision tool or a smoothing feature; however, both of these techniques have limitations. Mesh

subdivision tools increase file size, while smoothing tools can reduce the quality of features of interest. This is detailed in Case Study 1.

These issues can be improved through application of a shrinkwrap, which is described in all three case studies. Shrinkwrapping an object can solve these issues by creating a simplified 3D mesh of the object's surface shape. A shrinkwrap can be used to further combine multiple elements if desired. We demonstrate two methods by which to undertake shrinkwrapping: the first method utilizes Meshlab in Case Study 1 and 3, and the second utilizes Autodesk Fusion360 in Case Study 2.

Large file size: Exported meshes from 3D image processing software can often be large and computationally intensive to process. There are several methods to reduce total file size in Meshlab and Autodesk Fusion 360, such as applying tools that remove excess components or mesh simplification (described in Case Study 1 and 2). If only the surface structure is desired, another approach is to demarcate internal faces and vertices and select those for removal (described in Case Study 1). Many of these tools reduce the quality of the 3D model, so the approach and extent of file reduction should be appropriate for the desired image quality.

Data storage: Best practices for scientific investigations that generate 3D imaging data include making files openly accessible in perpetuity for other investigators. However, these datasets can be quite large (>10gb), making long-term file storage challenging. Several online data repositories are available for depositing and accessing 3D imaging data such as micro-CT datasets and 3D object files. These include MorphoSource

(morphosource.org), figshare (figshare.org), and Dryad (datadryad.org). 3D object files can be concurrently stored in an open 3D object repository such as Makerbot's Thingiverse, which has dedicated options for 3D printing (thingiverse.com), or Sketchfab, which has special options for creating virtual and augmented reality experiences (sketchfab.org). The unedited and edited 3D object files, as well as the original 3D imaging data, should be made accessible in perpetuity.

<u>Case studies in cleaning, repair, and reconstruction of digitized specimens:</u>

A variety of combined methods can be used to create a 3D digital model that is suitable for a diverse array of uses, ranging from 3D printing to biomechanical modeling. We present three case studies (one fossil plant, one vertebrate, and one arthropod), each with different post-processing needs, to demonstrate example workflows for creating 3D models of digitized specimens. The 3D object files used for model building were segmented and exported from micro-CT data using commercially available visualization and processing software (e.g. VG studio, Avizo) or obtained via a digital repository (e.g. Morphosource.org). These exported 3D object files of digitized specimens all required post-processing prior to use in downstream analyses. While segmentation software is often available at a cost, with some exceptions (e.g., SPIERS, 3D Slicer, Dragonfly), many of the post-processing software packages are freely available, particularly for student or educational use. These case studies highlight and address common problems that occur with 3D reconstructions of extant and fossil specimens. The amount and types of post-processing an individual 3D model requires is highly dependent on the specimen and intended use.

Results

Plant case study: *Petriellaea triangulata* (Fig 2)

Many fossil and extant plants possess unique anatomical features with distinctive shapes or unusual planes of symmetry. This can create challenges for generating 3D reconstructions, as certain processing tools are optimized for elongate structures (i.e., bones) or objects with bilateral symmetry. Here, we present a broadly applicable workflow for creating 3D digital reconstructions through a case-study using *Petriellea triangulata* (Shi et al. 2021). This specimen is a cupule of a fossil plant from the Early Cretaceous of Inner Mongolia ~~(Shi et al. 2021)~~. This specimen was segmented using a digital thresholding tool and extracted as a 3D object file from Avizo. Due to taphonomic affects and the segmenting process, the 3D object file representing the cupule contains preservational artifacts including ridges, rough surfaces, and holes. These issues were rectified by performing a protocol to shrinkwrap the specimen. Shrinkwrapping entails using the vertices that comprise the surface of the mesh to create a new mesh layer that simplifies the shape of the original 3D object. This fills holes and smooths uneven surfaces while minimizing the loss of anatomical fidelity. This process involves removing internal and external faces and internal vertices in Meshlab and applying a surface reconstruction tool to build a watertight mesh averaging the position of the remaining vertices.

Fig 2. 3D reconstruction of *Petriellaea triangulata* (Shi et al. 2021), before (a) and after (b) post-processing. A. 3D mesh (Shi et al. 2021) exported directly from Avizo version

9.2.0 (Thermo Fisher Scientific, Massachusetts, USA). Object file exhibits many of the common issues described for fossil taxa, including holes and irregular surfaces. B. 3D mesh file post-processed in Meshlab. Images generated using Autodesk Maya.

Vertebrate case study: *Sphenodon punctatus* (Fig 3)

Broken or missing elements are a common issue among specimens, particularly fossils. Even in well preserved specimens in which most elements are preserved *in situ* or are easily rearticulated, small holes exist. In this example, a mesh of a skull of *Sphenodon punctatus* (UF 11978, Florida Museum of Natural History, downloaded from Morphosource.org) was artificially damaged to replicate common breaks seen in extant and fossil specimens. A hole was created in the left frontal, and the right postorbital bar was removed to replicate common breaks seen in extant and fossil specimens.

Fig 3. 3D reconstruction of *Sphenodon punctatus* (UF 11978, Florida Museum of Natural History) before (a) and after (b) post-processing. 3D mesh was downloaded directly from Morphosource.org. (A) Mesh was artificially damaged to create a hole in the roof of the skull and a missing lower temporal bar (indicated by arrows) B. 3D mesh file post-processed in Autodesk Maya and Autodesk Fusion 360 with patched hole and reconstructed temporal bar. Images generated using Meshlab.

To patch the smaller hole in the frontal, a mesh cube was created in Autodesk Maya and then scaled to the approximate size of the hole. The edges of the cube were buried within the margins of the mesh of the skull, and the planar surface of the cube was

sculpted using translational and rotational tools. Once the cube was sculpted to match the surrounding areas of the skull, the two meshes were combined. To repair the right postorbital bar, a mesh cylinder was scaled and shaped to match the postorbital bar on the left side of the skull and was combined with the skull mesh. Depending on the use of the model, no further steps may be required. However, some subsequent analyses require a fully-flushed digital model, opposed to the combined meshes that are present in the skull of *Sphenodon punctatus* at this state. A shrinkwrap was applied in Autodesk Fusion 360 at this stage to create a subsequent surface model of the skull and the two patches. Further simplification of the model was also performed by reducing the face count in order to reduce the file size.

Invertebrate case study: *Cretapsara athanata* (Fig 4).

3D digital imaging of fossils can capture portions of rock matrix (i.e., debris) along with the fossilized organism. This material can often be easily removed in 3D visualization software during segmentation prior to 3D mesh creation. However, extraneous material may be of a similar density to the specimen itself or directly affixed to the digitized organism, and some additional processing may be necessary. A digitized fossil crab of *Cretapsara athanata* preserved in amber (Luque et al. 2021) represents the most complete fossil crab ever discovered to date. However, it still exhibits many common preservational issues, including the presence of external debris, disarticulated and missing elements, holes, and rough edges and irregular surfaces. After the crab was manually segmented and exported as a 3D mesh from VG Studio, post-processing was conducted in Meshlab and Autodesk Maya. The removal of small external debris was

automated in Meshlab because the 3D mesh of the crab has a much larger number of connected faces than the smaller, isolated elements desired for removal. Larger pieces of external debris and debris affixed to the specimen were removed manually. The debris removal process creates large gaps in the mesh that were further repaired in Meshlab. To do this, a copy of the mesh was shrinkwrapped by removing the internal faces and vertices, converting the mesh to a point cloud, and applying a surface reconstruction tool (Kazhdan and Hoppe 2013). Combining the shrinkwrapped mesh with a copy of the original mesh was performed to fill holes and smooth rough surfaces.

Fig 4. 3D reconstruction of *Cretapsara athanata* (Luque et al. 2021) before (a) and after (b) post-processing. (A) 3D mesh (Luque et al. 2021) exported directly from VG Studio MAX version 3.0. Object file exhibits many of the common issues described for fossil taxa, including the presence of external debris, disarticulated and missing elements, holes, and rough edges and irregular surfaces. (B) 3D mesh file post-processed in Meshlab and Autodesk Maya. Images generated using Autodesk Maya.

The eye as well as the dactyl and propodus (i.e., the claw) were preserved and extracted as a series of floating, isolated faces and vertices. These features were each isolated and shrinkwrapped separately due to relatively lower density of connected faces in these areas compared to the rest of the crab mesh. The left fifth leg was reattached by isolating the leg from the rest of the body in Meshlab, exporting both as separate object files, and repositioning the leg on the body in Maya. The left claw and the left eye were copied, reflected, and attached to the appropriate location on the right side.

Discussion

Different kinds of models are used across the natural sciences to illustrate components of complex phenomena, such as physical robots created to examine animal locomotion (e.g., Kano et al. 2017), fluid dynamic simulations to illustrate marine animal physiology (e.g., Gibson et al. 2021), atmospheric models designed to illuminate climate dynamics (e.g., Thomas and Federov 2019), or material deformation models to analyze plate tectonics (e.g., Mulyukova and Bercovici 2019). These models are all designed with the intended goal of reducing the complexity of the original system and to aid in analysis and subsequent interpretation. Similarly, simplified 3D models of organisms are designed to focus on specific anatomical features to illuminate their structure or function. By providing a compilation of techniques that address common issues in working with digitized biological and paleontological specimens, we address the first steps in undertaking more complex analyses that aid in understanding functional morphology. By removing extraneous noise, and adding detail that better represents true morphology, these refined models are capable of detecting morphological signals that, when subjected to downstream analyses, inform our understanding of these organisms and their physiology. As such, simplified models have become more commonplace in studies of functional morphology, homology, and paleoecology. Despite the simplified nature of these models, the interpretations made from them are crucial for understanding evolutionary phenomena over deep time.

However, the choice of techniques and the degree to which these approaches are put into effect determine the resulting 3D model and can impact the results of the

downstream analyses. Thus, aspects of the original specimen to include, simplify, or infer during the creation of a 3D model must be suitable for the type of analysis in question. For example, simplification of a digitized fossil is typically carried out prior to undertaking computational fluid dynamics simulations to reduce noise and facilitate computation (e.g., Rahman et al. 2015; Darroch et al. 2017; Gibson et al. 2021). Similarly, retrodeformation, translation, and the repair of broken elements may be appropriate to simulate *in vivo* anatomy for biomechanical analyses. Sculpting a missing appendage into a 3D model of a fossil organism based on its nearest ancestor would likely not be appropriate for geometric morphometrics, for instance, but may be appropriate for building a virtual reality experience for scientific outreach. It is crucial that the kind of model postprocessing used is appropriate for the downstream analysis in question.

Furthermore, steps used to create a 3D model must be transparent, justified, and accessibly detailed in all scientific publications in which they are used. The CT scan files, the unedited raw object files, and the edited 3D models should be made accessible in a repository for long-term availability, and data from multiple specimens should be used and provided where possible. This collection of software and techniques that span taxonomic groups provides a step towards the wider accessibility and collective exchange of post-processing strategies and technology in the study of extant and fossil organisms.

Conclusion

3D imaging tools and processing software are becoming more accessible for researchers. However, many issues can limit the usefulness of these 3D reconstructions for different analyses. Here, we provide the workflow used to repair 3D meshes of specimens in a

range of taxonomic groups extracted from micro-CT imaging data. These 3D models can be used for downstream analyses such as morphological descriptions, geometric morphometrics, and biomechanical modeling. These approaches have been applied to a wide range of taxa including and beyond the three case studies presented here. We hope that the presentation of our case studies and supplementary tutorials enables the broader integration of high-quality 3D reconstructions in biological and paleontological analyses.

Acknowledgements

We are grateful to the members of the Brodersen Lab, the Briggs Lab, the Gauthier Lab and the Bhullar Lab (Yale University), the Structure & Motion Laboratory (Royal Veterinary College) and the Almeida Lab (UC Berkeley) for assistance and valuable discussion.

References

Arbour, V. M., Currie, P. J. 2012. Analyzing taphonomic deformation of ankylosaur skulls using retrodeformation and finite element analysis. PloS ONE 7: e39323.

Bicknell, R. D. C., Klinkhamer, A. J., Flavel, R. J., Wroe, S, Paterson, J. R. 2018. A 3D anatomical atlas of appendage musculature in the chelicerate arthropod Limulus polyphemus. PLoS ONE 13(2): e0191400.

Brainerd, E. L., Baier, D. B., Gatesy, S. M., Hedrick, T. L., Metzger, K. A., Gilbert, S. L., Crisco, J. J. 2010. X-ray reconstruction of moving morphology (XROMM): precision, accuracy, and applications in comparative biomechanics research. Journal of Experimental Zoology 313A: 262-279.

Brodersen, C. R., Lee, E. F., Choat, B., Jansen, S., Phillips, R. J., Shackel, K. A., McElrone, A. J., Matthews, M. A. 2011. Automated analysis of three-dimensional xylemnetworks using high-resolution computed tomography. New Phytologist 191: 1168-1179.

Brodersen, C. R. 2013. Visualizing water transport in roots: advanced imaging tools for an expanding field. Plant Soil 366: 29–32.

Chambi-Trowell, S. A. V., Whiteside, D. I., Benton, M. J., Rayfield, E. J. 2020. Biomechanical properties of the jaws of two species of *Clevosaurus* and a reanalysis of rhynchocephalian dentary morphospaces. Palaeontology 63: 919-939.

Cignoni, P., Callieri, M., Corsini, M., Dellepiane, M., Ganovelli, F., Ranzuglia, G. 2008. MeshLab: an Open-Source Mesh Processing Tool. Sixth Eurographics Italian Chapter Conference, page 129-136, 2008

Clark, E.G., Hutchinson, J.R., Darroch, S.A.F., Mongiardino Koch, N., Brady, T.R., Smith, S.A., Briggs, D.E.G. 2018. Integrating morphology and *in vivo* skeletal mobility with digital models to infer function in brittle star arms. Journal of Anatomy 233: 696-714.

Clark, E. G., Hutchinson, J. R., Bishop, P., Briggs, D. E. G. 2020a. Arm waving in stylophoran echinoderms: three-dimensional mobility analysis illuminates cornute locomotion. Royal Society Open Science 7: 1-8.

Clark, E. G., Hutchinson, J. R., Briggs, D. E. G. 2020b. Three-dimensional visualization as a tool for interpreting locomotion strategies in ophiuroids from the Devonian Hunsrück Slate. Royal Society Open Science 7: 1-11.

Cunningham, J.A., Rahman, I.R., Lautenschlager, S., Rayfield, E.J., Donoghue, P.C.J. 2014. A virtual world of paleontology. Trends in Ecology & Evolution 29: 347-357.

Darroch, S .A. F., Rahman, I. A., Gibson, B., Racicot, R. A., Laflamme, M. 2017. Inference of facultative mobility in the enigmatic Ediacaran organism *Parvancorina*. Biology Letters 13: 1-5.

Degrange, F. J., Tambussi, C. P., Moreno, K., Witmer, L. M., Wroe, S. 2010. Mechanical analysis of feeding behavior in the extinct “Terror Bird” *Andalgalornis steulleti* (Gruiformes: Phorusrhacidae). PloS ONE 5: e11856.

DeVries, R. P., Sereno, P. C., Vidal, D., Baumgart, S. L. 2022. Reproducible digital restoration of fóssil using *Blender*. Frontiers in Earth Science 10: 1-13.

Gibson, B. M., Darroch, S. A. F., Maloney, K. M., Laflamme, M. 2021. The importance of size and location within gregarious populations of *Ernietta plateauensis*. Frontiers in Earth Science 9: 1-11.

Grine, F. E., Gunz, P., Betti-Nash, L., Neubauer, S., Morris, A. G. 2010. Reconstruction of the late Pleistocene human skull from Hofmeyr, South Africa. Journal of Human Evolution 59: 1-15.

Gunz, P., Mitteroecker, P., Neubauer, S., Weber, G. W., Booksteinm F. L. 2009. Principles for the virtual reconstruction of hominin crania. Journal of Human Evolution 57: 48-62.

Hopkins, M. J., Pearson J. K. 2016. Non-linear ontogenetic shape change in *Cryptolithus tesselatus* (Trilobita) using three-dimensional geometric morphometrics. Palaeontologica Electronica 19.3.42A: 1-54.

Kano, T., Sato, E., Ono, T., Aonuma, H., Matsuzaka, Y., Ishiguro, A. 2017. A brittle star-like robot capable of immediately adapting to unexpected physical damage. Royal Society Open Science 4: 171200.

Kazhdan, M. and Hoppe, H. 2013. Screened Poisson surface reconstruction. ACM Trans. Graphics, 32(3).

Lautenschlager, S. 2016. Reconstructing the past: methods and techniques for the digital restoration of fossils. Royal Society Open 3: 160342.

Liu et al. 2021 Liu, Y., Edgecombe, G. D., Schmidt, M., Bond, A. D., Melzer, R. R., Zhai, D., Mai, H., Zhang, M., Hou, X. 2021. Exites in Cambrian arthropods and homology of arthropod limb branches. Nature Communications 12: 1-7.

Luque, J., Xing, L., Briggs, D. E. G., Clark, E. G., Duque, A., Hui, J., Mai, H., McKellar, R. C. 2021. Crabs in amber reveal an early colonization of freshwater during the Cretaceous. Science Advances 7: 1-12.

Molnar, J. L., Pierce, S. E., Clack, J. A., Hutchinson, J. R. 2012. Idealized landmark-based geometric reconstructions of poorly preserved fossil material: a case study of an early tetrapod vertebra. Palaeontologia Electronica 15: 1-18.

Molnar, J. L., Hutchinson, J. R., Diogo, R., Clack, J. A., Pierce, S. E. 2021. Evolution of forelimb musculoskeletal function across the fish-to-tetrapod transition. Science Advances 7: 1-11.

Morón-Alfonso, D. A., Peterman, D. J., Cichowolski, M., Hoffman, R., Lemanis, R. E. 2020. Virtual 3D modeling of the ammonoid conch to study its hydrostatic properties. Acta Palaeontologica Polonica 65: 467-480.

Mulyukova, E. and Bercovici, D. 2019. The generation of plate tectonics from grains to global scales: a brief review. Tectonics 38: 4058-4076.

Ogihara, N., Nakatsukasa, M., Nakano, Y., Ishida, H. 2006. Computerized restoration of nonhomogeneous deformation of a fossil cranium based on bilateral symmetry. American Journal of Physical Anthropology 130: 1-9.

Pintore, R., Delapre, A., Lefebvre, R., Botton-Divet, L., Houssaye, A., Cornette, R. The potential and limits of *Thin-Plate Spline* retrodeformation on asymmetrical objects: simulation of taphonomic deformations and application on a fossil sample of limb long bones. Comptes Rendus Palevol 21: 181-205.

Rahman, I. A., Darroch, S. A. F., Racicot, R. A., Laflamme, M. A. 2015. Suspension feeding in the enigmatic Ediacaran organism Tribrachidium demonstrates complexity of Neoproterozoic ecosystems. Scientific Advances 1: 1-8.

Rahman, I. A., Lautenschlager, S. 2017. Applications of three-dimensional box modeling to paleontological functional analyses. The Paleontological Society Papers 22:119-132.

Rahman, I. A., O'Shea, J., Lautenschlager, S., Zamora, S. 2020. Potential evolutionary trade-off between feeding and stability in Cambrian cinctan echinoderms. Palaeontology 62: 689-701.

Rayfield, E. J. 2007. Finite element analysis and understanding the biomechanics and evolution of living and fossil organisms. Annu. Rev. Earth Planet. Sci. 35: 541-576.

Shi, G., Herrera, F., Herendeen, P. S., Clark, E. G., Crane, P. R. 2021. Mesozoic cupules and the origin of the angiosperm second integument. *Nature* 594, 223-226.

Sutton, M., Rahman, I., Garwood, R. 2017. Virtual paleontology—an overview. The Paleontological Society Papers 22: 1-20.

Thomas, M. D., Fedorov, A. V. 2019. Mechanisms and impacts of a partial AMOC recovery under enhanced freshwater forcing. Geophysical Research Letters 46: 3308-3316.

Wason, J. W., Brodersen, C. R., and Huggett, B. A. 2019. The functional implications of tracheary connections across growth rings in four northern hardwood trees. Annals of Botany 124: 297-306.

Young, M. T., Rayfield, E. J., Holliday, C. M., Witmer, L. M., Button, D. J., Upchurch, P., Barrett, P. M. 2012. Cranial biomechanics of *Diplodocus* (Dinosauria, Sauropodomorpha): testing hypotheses of feeding behaviour in an extinct megaherbivore. Naturwissenschaften 99: 637-643.

Zollikofer, C. P. E. 2002. A computational approach to paleoanthropology. Hominid Evolution 11: 64-67.

Zollikofer, C. P. E., Ponce de León, M. S. 2005. Virtual reconstruction: a primer in computer-assisted paleontology and biomedicine. Wiley-Intersicence: New Jersey.

Supplemental Materials

S1 is a compressed (.zip) file that includes:

- Beginner tutorial: Fossil plant cupule (*Petriellaea triangulata* (Shi et al. 2021))
  - *Included files:* Cupule Tutorial (.docx), 3D object files (Cupule before.stl, Cupule before.obj)
  - *Necessary software*: Meshlab
  - *Skills developed*: Shrinkwrapping, simplification
- Intermediate tutorial: Reptile (*Sphenodon punctatus* [downloaded from Morphosource.org])
  - *Included files*: Sphenodon Tutorial (.docx), 3D object files (Sphenodon with holes.stl, Sphenodon with holes.obj)
  - *Necessary software*: Meshlab, Autodesk Maya, Autodesk Fusion 360
  - *Skills developed*: Rearticulation, repair of broken fossils, basic sculpting, shrinkwrapping.
- Advanced tutorial: Fossil crab (*Cretapsara athanata* [Luque et al. 2021)]
  - *Included files:* Tutorial (.docx), 3D object files (Crab before.stl, Crab before.obj)
  - *Necessary software*: Meshlab, Autodesk Maya
  - *Skills developed*: Debris removal, structural repair, shrinkwrapping, rearticulation, smoothing and simplification

Figure 1

Click here to access/download;Figure;Fig1.tiff

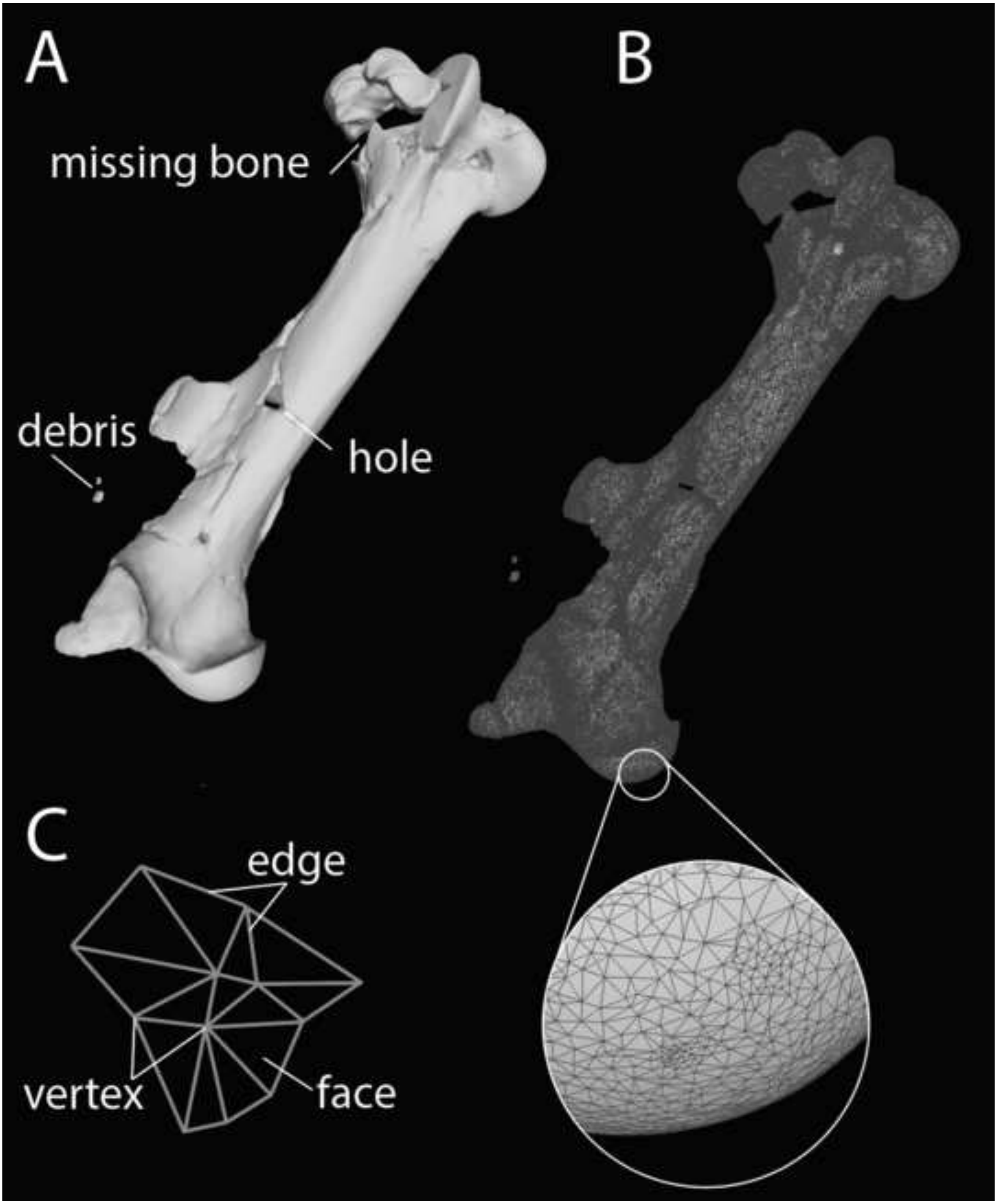

Figure 2

Click here to access/download;Figure;Fig2.tiff

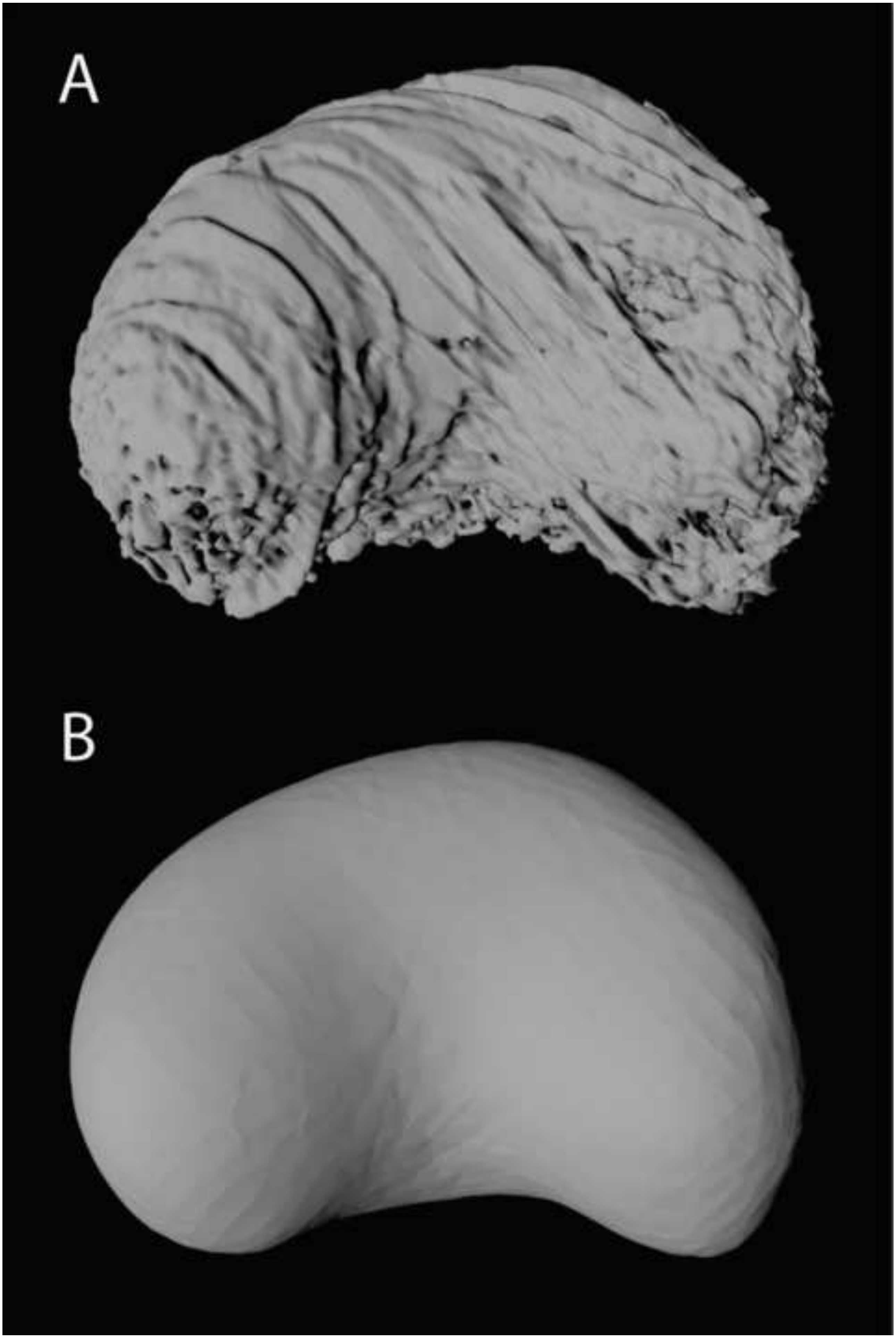

Figure 3

Click here to access/download;Figure;Fig3.tiff

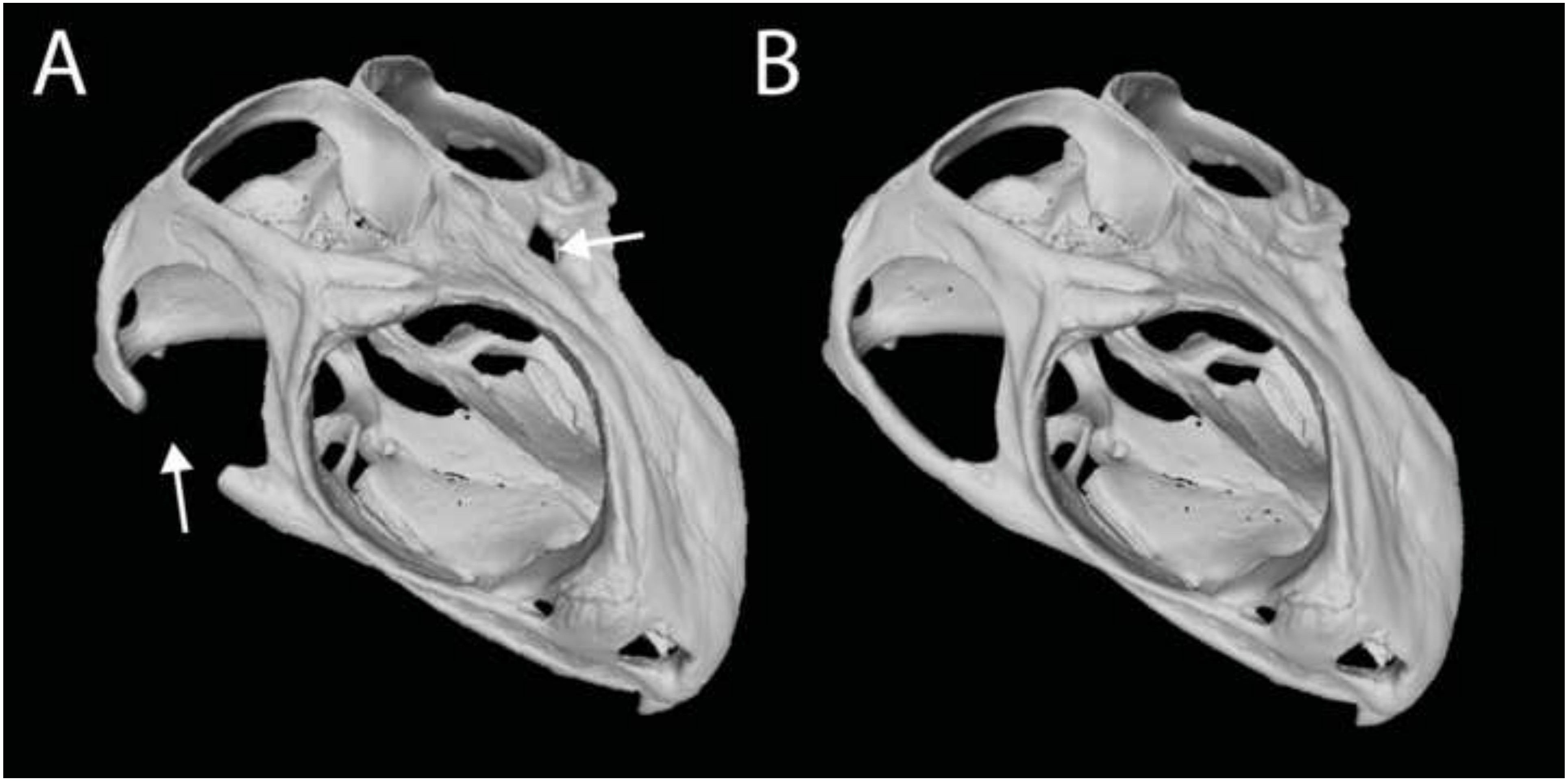

Figure 4

Click here to access/download;Figure;Fig4.tiff

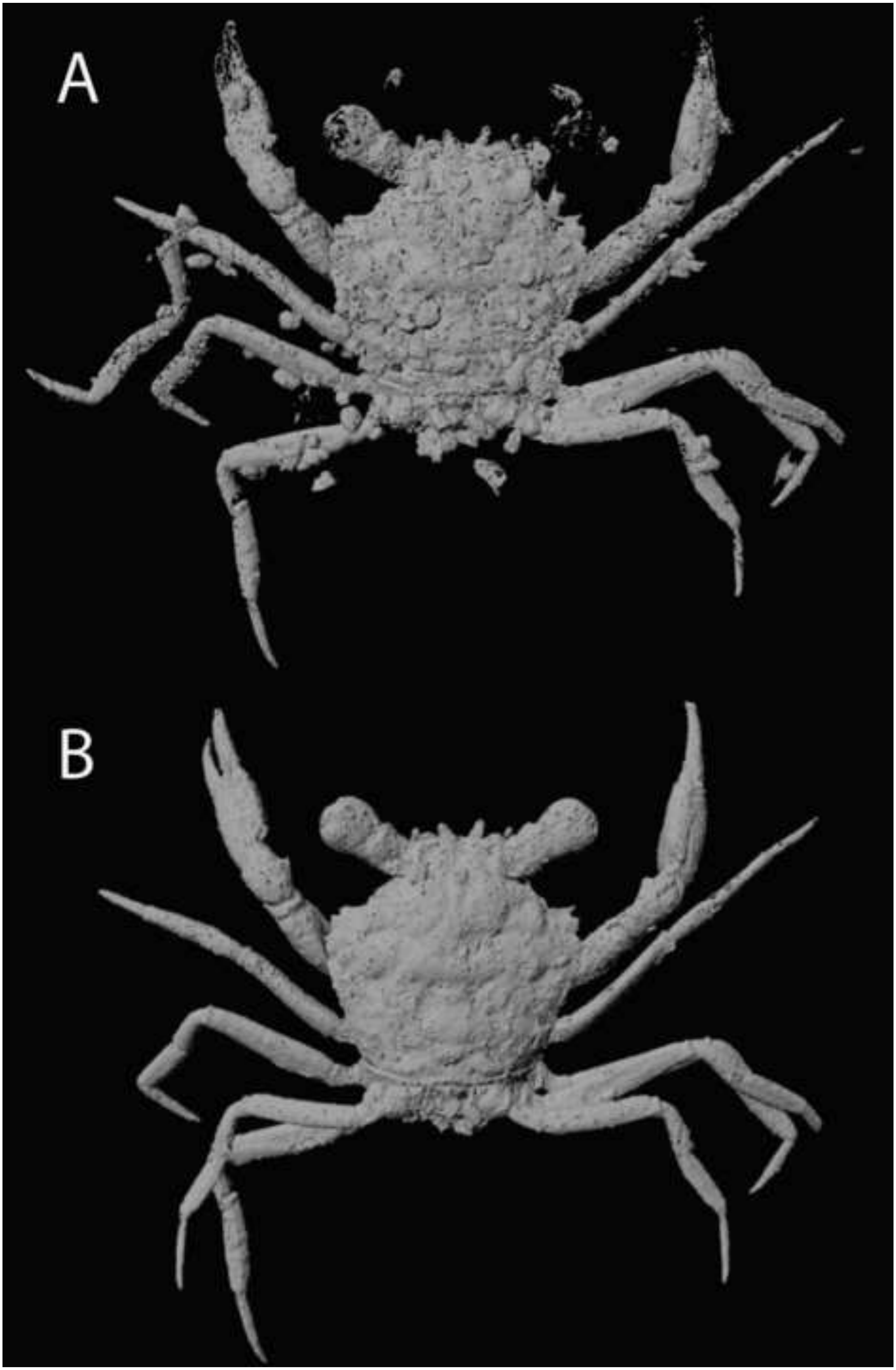

S1 File

Click here to access/download
Supporting Information
S1_file.docx
